# Electrolyte additive engineering to construct stable interphases with high ionic conductivity for high-temperature and high-voltage lithium metal batteries

**DOI:** 10.1039/d6sc04807b

**Published:** 2026-07-17

**Authors:** Yanan Li, Wenzhe Zhang, Xiaosha Wu, Rui Yu, Kun Wang, Kai Xi, Shujiang Ding, Guoxin Gao

**Affiliations:** a School of Chemistry, Engineering Research Center of Energy Storage Materials and Devices, Ministry of Education, National Innovation Platform (Center) for Industry-Education Integration of Energy Storage Technology, Xi'an Jiaotong University Xi'an 710049 P. R. China kx210.cam@xjtu.edu.cn dingsj@mail.xjtu.edu.cn gaoguoxin@mail.xjtu.edu.cn

## Abstract

Integrating high-voltage nickel-rich cathodes with lithium metal anodes while elevating charging cut-off voltage is a practical strategy for constructing high-energy-density battery systems. Nevertheless, severe structural degradation of cathodes and aggressive electrode/electrolyte interfacial reactions hinder their application. Worse still, these issues are exacerbated under high-voltage and high-temperature operating conditions. Herein, we propose a bifunctional electrolyte with 4-trifluoromethylbenzeneboronic acid neopentyl glycol ester (TFMB) as an additive to tailor robust interphases, which alleviates cathode structural damage and suppresses lithium dendrite growth. The electron-deficient boron in TFMB coordinates with anions, thereby enhancing the thermal stability of electrolytes and facilitating the involvement of anions in the formation of interphases. Meanwhile, fluorinated functional groups contribute to the formation of stable, inorganic-rich interphases and further boost the high-voltage resistance of batteries. Therefore, Li‖Ni_0.8_Co_0.1_Mn_0.1_O_2_ (NCM811) cells maintain a superior reversible capacity retention of 75.25% after 300 cycles at 4.5 V and achieve outstanding cycling stability with 80% capacity retention over 120 cycles at 4.7 V. Impressively, Li‖NCM811 cells demonstrate favorable capacity retention even at 50 °C, validating their application prospects. Furthermore, its good compatibility with LiFePO_4_ cathodes meets the demands of different energy scales. This work provides meaningful insights for designing high-voltage and high-temperature electrolytes for lithium metal batteries.

## Abstract 2

The electrolyte with 4-trifluoromethylbenzeneboronic acid neopentyl glycol ester as an additive can form ion-conductive and robust interphases to improve the long-term performance of lithium metal batteries at high temperature and high voltage.

## Graphical abstract

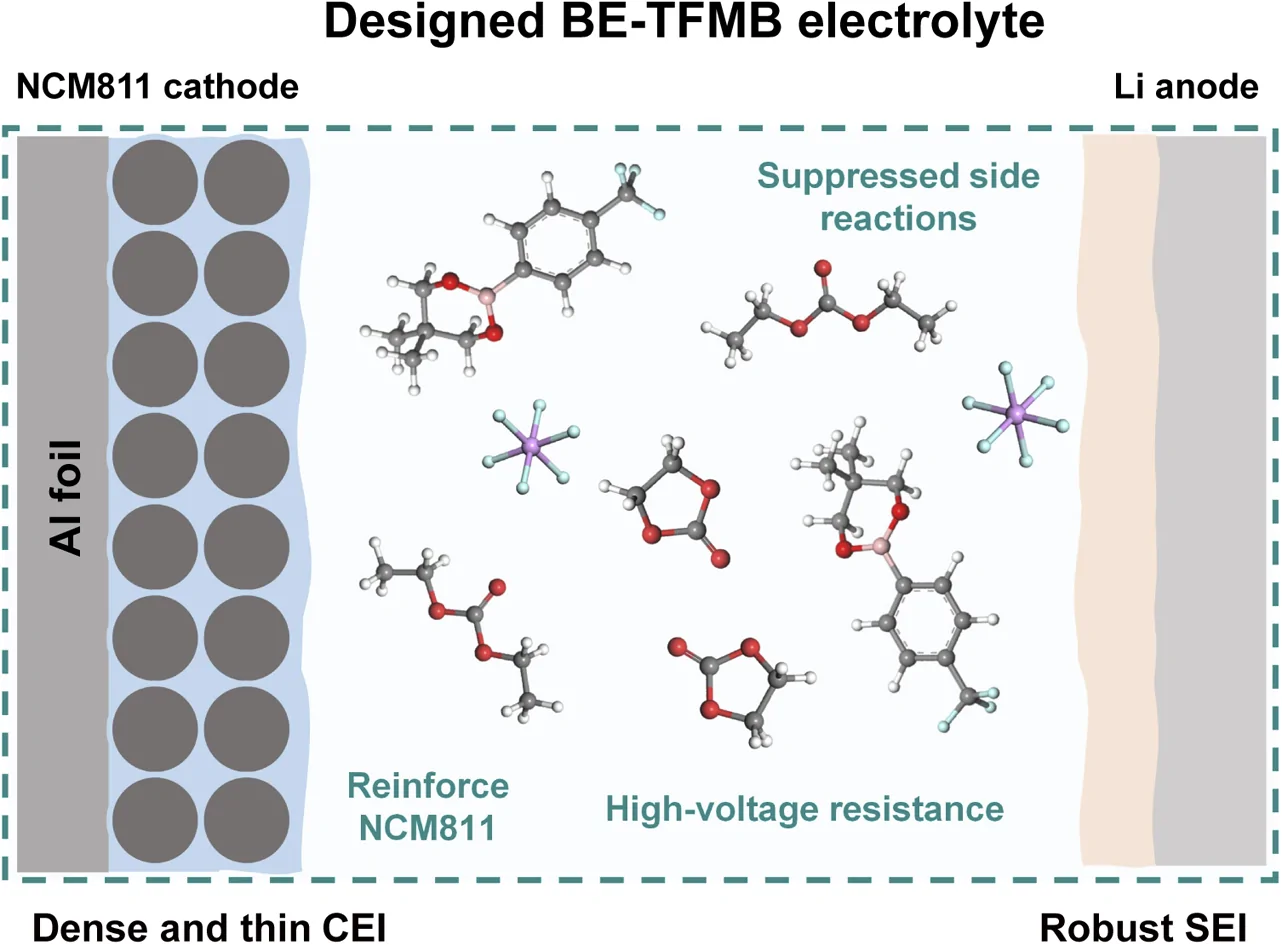

## Introduction

The advancement of electric vehicles and energy storage systems has imposed greater demands on safe, sustainable and high-energy-density batteries, driving research into pairing high-capacity cathodes with lithium metal anodes (LMAs). Since energy density is determined by discharge capacity and average discharge voltage, the development of cathode materials with both high capacity and enhanced working voltage is a pressing priority. Despite being widely used, conventional LiCoO_2_ (LCO, Li/Li^+^ of 3.7 V, 140 mAh g^−1^) and LiFePO_4_ (LFP, Li/Li^+^ of 3.2 V, 170 mAh g^−1^) cathodes fail to deliver satisfactory energy densities.^1,2^ In contrast, nickel-rich layered oxides (LiNi_*x*_Co_*y*_Mn_1−*x*−*y*_O_2_, *x* ≥ 0.8, NCM) stand out as promising candidates due to their superior theoretical capacity (>200 mAh g^−1^) and elevated output voltage (3.8 V). Moreover, further increasing the upper cutoff voltage of these cathodes is considered an effective strategy to improve battery energy density.^3^ However, high-nickel cathodes are prone to irreversible phase transitions, the dissolution of transition metal ions (TMIs), and severe parasitic electrolyte side reactions. Notably, during long-term cycling, the high reactivity of LMAs leads to persistent side reactions and the continuous thickening of the solid electrolyte interphase (SEI), which increase the resistance of the SEI and finally cause rapid battery failure.^4,5^ Meanwhile, uneven Li deposition and uncontrolled dendritic growth bring about severe safety hazards.^6^ Worse still, these issues are further exacerbated under extreme conditions, hindering the development of high-energy-density lithium metal batteries (LMBs).

Generally, high temperatures aggravate the intrinsic instability of electrolytes and induce violent side reactions, posing a serious threat to the stability of interphases and the safety of batteries. Simultaneously, the deep delithiation at high temperatures impairs the structural reversibility of cathodes and compromises the integrity of the cathode electrolyte interphase (CEI).^7^ In addition, HF generated by the hydrolysis of Li salts readily causes severe corrosion and cracking of the electrodes and current collectors.^8^ Importantly, elevated cut-off voltage worsens the collapse of the cathode structure, while the accompanying gas evolution from lattice oxygen leads to battery swelling.^9^ Furthermore, accelerated kinetics at high temperatures and polarization under high voltage exacerbate Li dendrite growth. The frequent rupture and reconstruction of the SEI constantly deplete electrolyte and active Li^+^, further inducing safety issues such as thermal runaway. Currently developed cathode modification strategies including coating, doping and single-crystallization can achieve certain improvements in stability, yet they are limited by complex processes, high costs and insufficient regulation accuracy.^10,11^ Similarly, existing designs such as artificial SEI and alloy modification still urgently require enhancement in the mechanical stability and long-term cycling performance of LMAs.^12^ Notably, the composition and structure of electrode-protective interphases predominantly govern the long-term performance of batteries, which are determined by electrolytes in contact with both the cathodes and anodes. Therefore, developing an advanced electrolyte system capable of synergistically stabilizing interphases and electrodes is crucial.

The traditional LiPF_6_-based carbonate electrolytes feature high ionic conductivity and low cost. However, the inevitable hydrolysis of LiPF_6_ generates harmful substances (such as HF), which can corrode electrode materials and deteriorate battery performance. Furthermore, the poor thermodynamic stability and high reactivity of carbonate solvents also pose critical challenges.^13^ To address these drawbacks, high-concentration and localized high-concentration electrolytes have been developed to facilitate the formation of anion-enriched solvation structures. Such methods not only promote Li^+^ desolvation, but also aid in the formation of robust, inorganic-rich interphases, thereby endowing LMBs with good cycling stability and excellent reversibility. Regrettably, these high-concentration systems exhibit inferior wettability, low ionic conductivity and unsatisfactory economic efficiency.^14^ In contrast, additive engineering is a simple and cost-effective means of electrolyte regulation. Additives can react preferentially and participate in the formation of interphases, which inhibits side reactions and prolongs the cycling life of batteries. Moreover, the composition and structure of interphases can be regulated based on the molecular structures, functional groups, and reaction mechanisms of additives.^15^ Specifically, B-containing additives show Lewis acidity, which can bind with anions to promote their participation in interphase formation and improve the thermal stability of the electrolyte.^16^ In addition, F-containing groups aid in the formation of a stable LiF-rich interphase, which contributes to improving the high-voltage resistance of batteries.^17^ Consequently, it is highly necessary to develop B- and F-containing additives for high-temperature and high-voltage tolerant electrolytes applicable to high-nickel based LMBs.

**Fig. 1:**
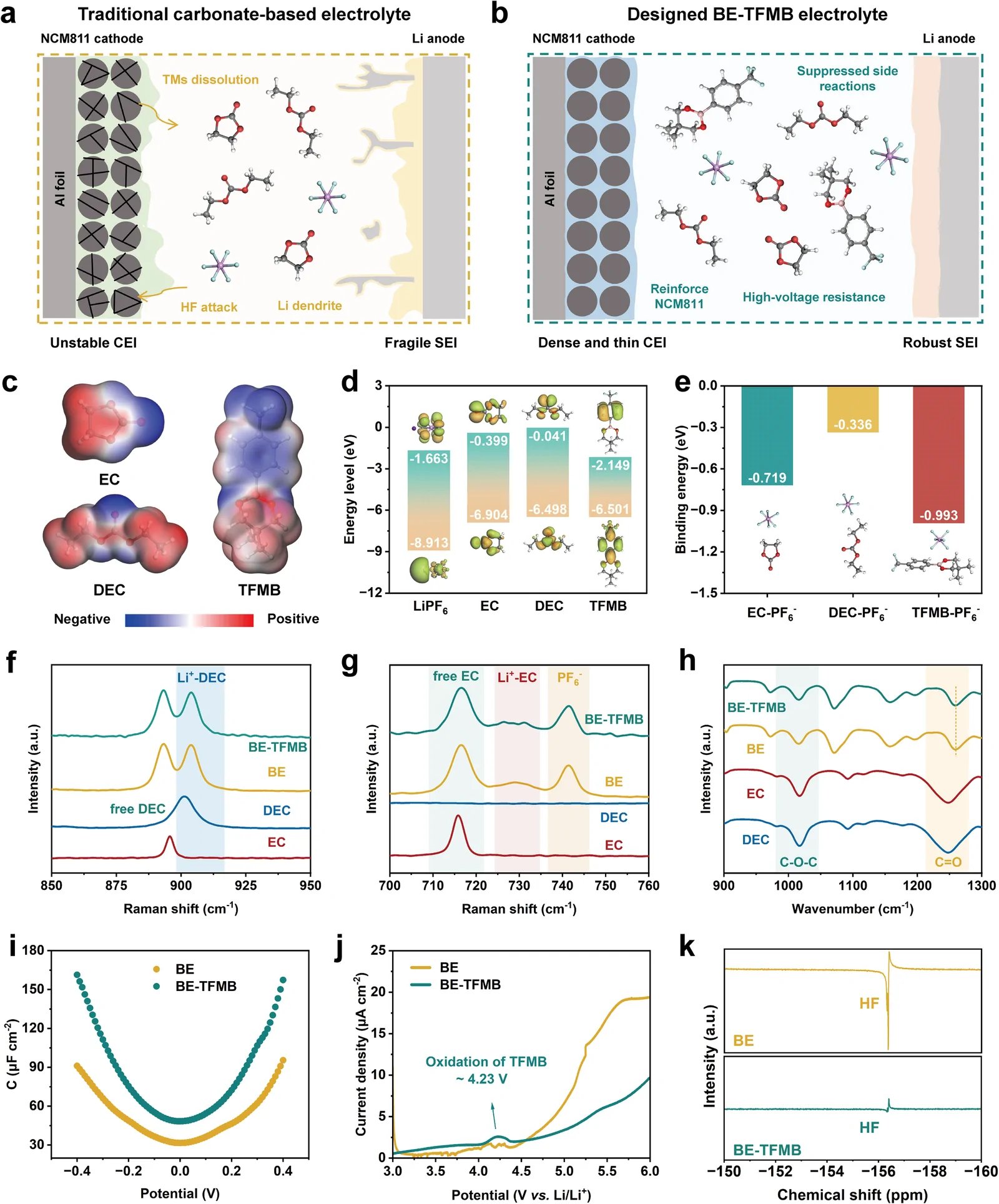
Schematic diagrams of (a) traditional carbonate-based and (b) designed BE-TFMB electrolytes. (c) ESP maps of EC, DEC and TFMB. (d) The HOMO and LUMO energies of EC, DEC, TFMB and LiPF_6_. (e) Binding energies of EC-PF_6_^−^, DEC-PF_6_^−^ and TFMB-PF_6_^−^. Raman spectra of EC, DEC, BE and BE-TFMB at (f) 850–950 cm^−1^ and (g) 700–760 cm^−1^. (h) FT-IR spectra of EC, DEC, BE and BE-TFMB at 900–1300 cm^−1^. (i) Differential capacitance curves of the two electrolytes. (j) LSV curves of BE and BE-TFMB. (k) ^19^F-NMR spectra of BE and BE-TFMB stored for 30 days.

Herein, we propose an additive-assisted bifunctional electrolyte for stable operation of LMBs under extreme conditions. Specifically, 1 wt% 4-trifluoromethylbenzeneboronic acid neopentyl glycol ester (TFMB) is added to a base electrolyte consisting of 1 M LiPF_6_ dissolved in ethylene carbonate and diethyl carbonate (EC : DEC = 1 : 1, v/v). TFMB features electron-deficient B atoms, which can bind to PF_6_^−^ anions, alleviate LiPF_6_ hydrolysis-induced corrosion of electrodes, and lower the oxidation potential of anions to form a robust CEI. Furthermore, the highly electronegative –CF_3_ groups contribute to forming an inorganic-rich interphase. As anticipated, the optimized electrolyte shows high ionic conductivity, good high-voltage tolerance and excellent inherent chemical stability. Combined results indicate that TFMB induces the formation of a stable, inorganic-rich CEI, as well as a SEI with an outer organic layer and inner inorganic layer. These interphases inhibit excessive electrolyte penetration and severe side reactions, thereby mitigating structural degradation of cathodes and Li dendrite growth (Fig. 1a and b). Accordingly, the modified electrolyte demonstrates good compatibility with LiNi_0.8_Co_0.1_Mn_0.1_O_2_ (NCM811) cathodes and LMAs, enabling Li‖NCM811 cells to maintain 75.25% of the initial capacity after 300 cycles at 4.5 V. When the charging cut-off voltage is further increased to 4.7 V, the assembled Li‖NCM811 cells still achieve 80% capacity retention over 120 cycles at 1C. Benefiting from the TFMB-derived interphases, Li‖NCM811 cells also exhibit a steady capacity output of 212.27 mAh g^−1^ at 50 °C and 4.5 V with a retention of 80.14% after 100 cycles. More importantly, this additive modification strategy is feasible for Li‖LFP cells, providing possibilities for various energy supply requirements. Overall, this work clarifies the connection between additive design and interphase structure, offering an effective paradigm of electrolyte design for high-temperature and high-voltage LMBs.

## Results and discussion

First, the physicochemical properties of the electrolytes were explored. The base electrolyte (BE) containing 1 wt% TFMB is denoted as BE-TFMB in subsequent discussions. Electrostatic potential (ESP) calculations reveal that TFMB contains B atoms with positive electrostatic characteristics. Its electron-deficient feature favors the coordination with PF_6_^−^ and reduces the oxidation potential of anions, ultimately facilitating the formation of an inorganic-rich CEI.^18^ Moreover, the highly electronegative –CF_3_ groups exhibit negative ESP distributions, and their high electron affinity contributes to the preferential formation of a LiF-rich SEI (Fig. 1c). Consistent conclusions are also deduced from the highest occupied molecular orbital (HOMO) and lowest unoccupied molecular orbital (LUMO) levels, which are adopted to assess the redox properties of additives (Fig. 1d). Compared with solvent molecules, TFMB exhibits a lower LUMO energy and a moderate HOMO energy, enabling it to preferentially regulate the LiF- and B–F-rich interphases. XPS data in Fig. S1 show that the electrodes after initial cycling in BE-TFMB demonstrate a distinct B signal. The corresponding high B–F content also serves as evidence that TFMB is preferentially reduced. Moreover, the SEM images and corresponding mapping images show a uniform distribution of B signals of BE-TFMB-derived electrodes (Fig. S2 and S3). Besides, the competitive binding energy of BE-TFMB toward PF_6_^−^ can facilitate the involvement of anions in the formation of interphases (Fig. 1e and S4).^19^ As shown in Fig. S5, the ^7^Li NMR spectra in BE-TFMB exhibit an upfield shift, indicating an enhanced shielding effect of Li^+^.^20^ Furthermore, the corresponding ^19^F NMR spectra show a downfield shift in the peak of PF_6_^−^, which is attributed to its increased coordination with Li^+^.^21^ Small-angle X-ray scattering (SAXS) measurements show that the peak gradually shifts toward lower *q* values (14.47 nm^−1^), indicating a decrease in the average distance between PF_6_^−^ anions (Fig. S6).^22^ Additionally, Fig. S2 and S3 show that the surfaces of the BE-TFMB-derived electrodes exhibit stronger F signals, collectively confirming the regulatory role of TFMB in the formation of interphases. Upon TFMB incorporation into the BE, the resultant BE-TFMB displays lower capacitance values. This phenomenon originates from the adsorption of TFMB on the electrodes, which increases the effective thickness of the electrical double layer (Fig. 1i).^23^

To investigate the compatibility of BE-TFMB with the high-voltage NCM811 cathode, the electrochemical oxidation stability of the two electrolytes was evaluated *via* linear sweep voltammetry (LSV) tests. Compared with BE, BE-TFMB demonstrates excellent high-voltage stability. A distinct oxidation peak located at 4.23 V is assigned to the electrochemical oxidation of TFMB. Notably, the rapid increase in current of BE-TFMB is suppressed upon voltage elevation, indicating suppressed side reactions (Fig. 1j). The same result is observed in the reductive LSV curves, where the reduction currents of BE-TFMB are lower than those of BE (Fig. S7). LiPF_6_-based carbonate ester electrolytes easily undergo severe hydrolysis during long-term storage, and the resulting products can severely damage the electrodes and electrode/electrolyte interphases. Hence, the intrinsic chemical stability is crucial for practical electrolytes. As shown in Fig. S8 and S9, the addition of TFMB did not cause any changes in the chemical composition of BE-TFMB, proving that no chemical reactions occurred. In addition, the positions and intensities of Li^+^-solvated EC and DEC peaks at 728.69 cm^−1^ and 903.79 cm^−1^ remain virtually unchanged (Fig. 1f and g). Consistent evidence is also found in Fourier transform infrared (FT-IR) spectra (Fig. 1h).^24^ Nuclear magnetic resonance spectroscopy (NMR) was further applied to monitor the long-term storage stability of electrolytes. ^19^F NMR spectra in Fig. 1k show that even after 30 days of storage, BE-TFMB presents a weaker HF signal than BE, indicating its excellent stability. Furthermore, the time-dependent ^19^F spectra at 60 °C show that the concentration of HF in BE rises rapidly with prolonged heating, stirring, and storage. In contrast, the contents of HF in BE-TFMB are continuously lower than those of BE (Fig. S10 and S11 and Table S1).^25^ The temperature-dependent Raman spectra also show that the peak of PF_6_^−^ at 742 cm^−1^ in BE continues to weaken over time at 60 °C, indicating that high temperature accelerated the hydrolysis of LiPF_6_. Clearly, the signal of PF_6_^−^ in BE-TFMB remains distinct, verifying that the hydrolysis process of LiPF_6_ is significantly restrained (Fig. S12). As a result, owing to the interaction between TFMB and PF_6_^−^, the intrinsic stability of BE-TFMB is improved.

The impacts of TFMB on the transport properties of BE-TFMB were further investigated. As shown in Fig. S13, the contact angles of the two electrolytes on Celgard 2325 separators and NCM811 cathodes were measured. The smaller contact angles reveal superior wettability of BE-TFMB towards the separator and cathode, thereby favoring fast Li^+^ transport. Notably, B atoms in TFMB act as anion receptors to coordinate with PF_6_^−^ and promote the dissociation of Li salts, and thus, BE-TFMB consistently exhibits higher ionic conductivity than BE over a wide temperature range from −10 °C to 60 °C (Fig. S14 and Table S2). This conclusion is also validated by Li^+^ transference number (*t*_Li^+^_) measurements. BE-TFMB shows a higher *t*_Li^+^_ value (0.52) than BE (0.41) (Fig. S15). Consequently, BE-TFMB possesses outstanding stability and ion transport properties, making it promising for tailoring robust interphases for high-performance LMBs.

**Fig. 2:**
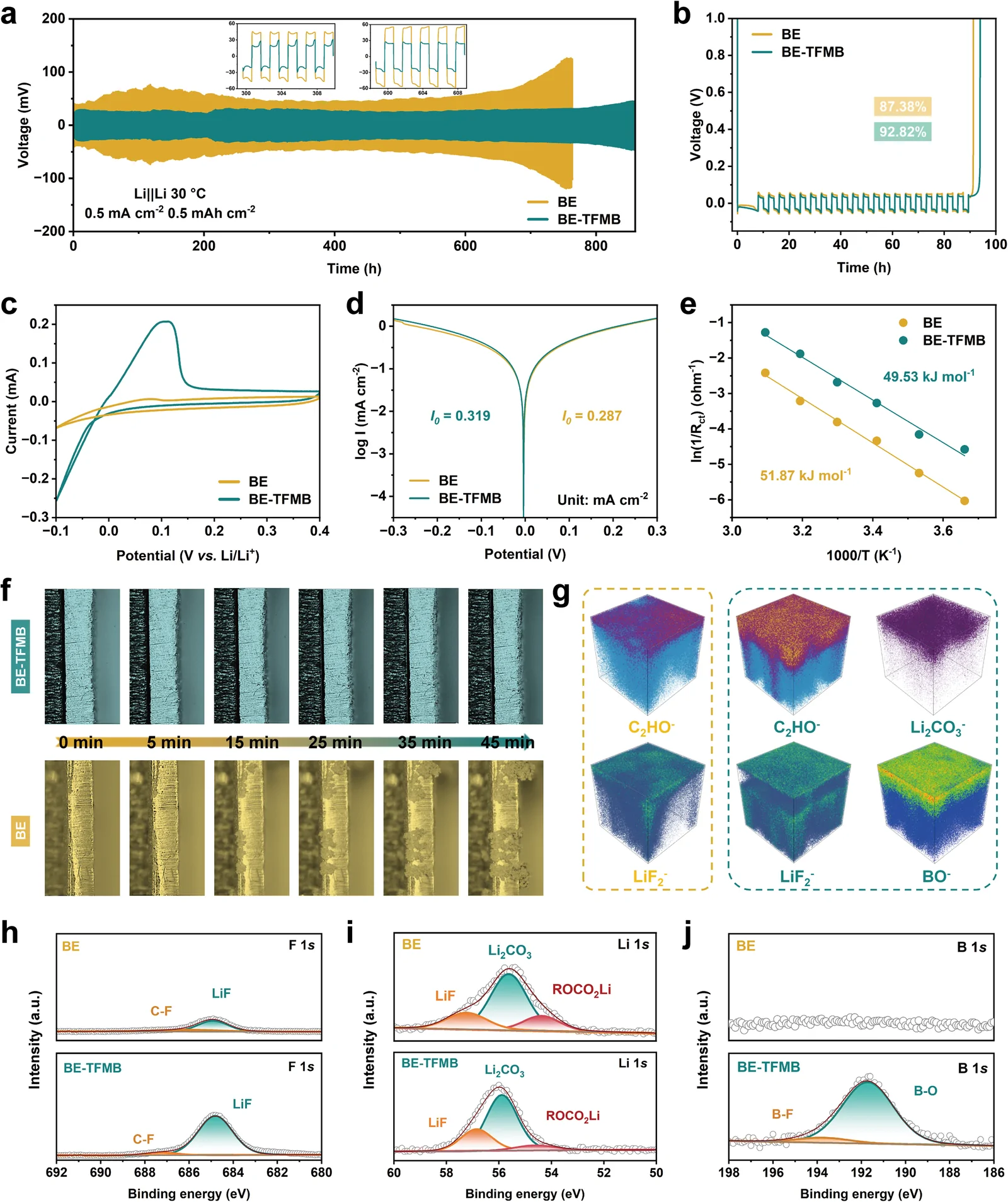
(a) Cycling stability of symmetric Li‖Li cells in two electrolytes. (b) Li plating/stripping CEs of Li‖Cu cells evaluated *via* Aurbach's measurement. (c) CV curves of Li‖Cu cells. (d) Tafel curves of Li‖Li cells containing two electrolytes. (e) Activation energies of Li^+^ desolvation of BE and BE-TFMB. (f) *In situ* optical images of Li deposition in BE and BE-TFMB. (g) 3D rendering images of LiF_2_^−^, C_2_HO^−^, Li_2_CO_3_^−^ and BO^−^ in the BE-derived SEI and BE-TFMB-derived SEI. XPS spectra of (h) F 1s, (i) Li 1s, and (j) B 1s for cycled Li anodes in the two electrolytes.

To verify the modulation effect of TFMB on Li anode behavior, symmetric Li‖Li cells were assembled to evaluate the cycling performance (Fig. 2a). Li‖Li cells containing BE-TFMB exhibit excellent cycling stability over 860 h with slow overpotential growth, while cells using BE show a high overpotential after only 600 h. The sharply increasing overpotential reflects severe polarization in the BE-based cells. Furthermore, Li‖Cu cells were employed to characterize the coulombic efficiency (CE) during Li plating/stripping in different electrolytes. As shown in Fig. 2b, severe side reactions and unstable interphases in BE lead to a low CE (87.38%). Conversely, cells containing BE-TFMB demonstrate a higher CE of 92.82% and good reversibility of Li plating/stripping. Notably, the excellent reproducibility of this result was confirmed in multiple cells (Fig. S16). Subsequently, the kinetic features of the two electrolytes were further explored. Li‖Cu cells with BE-TFMB also deliver an enhanced current response, confirming the faster interfacial reaction kinetics of BE-TFMB (Fig. 2c). In addition, the lower initial deposition potential indicates a lower nucleation barrier and faster ion transport (Fig. S17).^26^ As illustrated in Fig. 2d, BE-TFMB shows a higher exchange current density of 0.319 mA cm^−2^ than 0.287 mA cm^−2^ for the BE system, revealing better interfacial contact and more rapid interfacial kinetics. Subsequently, analysis using the simplified Butler–Volmer equation supports this conclusion. The remarkably higher *i*_0_ value indicates enhanced Li^+^ transport in BE-TFMB (Fig. S18).^27^ Notably, the *E*_ct_ acquired from the Arrhenius equation demonstrates that BE-TFMB shows fast desolvation over a wide temperature range. Compared with the higher *E*_ct_ (51.87 kJ mol^−1^) of BE, BE-TFMB exhibits a superior desolvation ability (49.53 kJ mol^−1^) (Fig. 2e and S19). In summary, the robust SEI induced by TFMB helps suppress parasitic electrolyte reactions and facilitate Li^+^ transport, playing a crucial role in the rate performance of the cells.

Comprehensive analysis of the SEI elucidates the mechanism by which TFMB improves its compatibility with Li metal and Li^+^ transport efficiency. Top-view scanning electron microscopy (SEM) images show that the cycled Li anode in BE-TFMB exhibits a smooth and dense surface without Li dendrites. By contrast, Li deposits in BE are accompanied by severe dendrite growth and abundant pores (Fig. S20). The unstable SEI struggles to accommodate the electrode volume changes upon cycling, which leads to continuous electrolyte penetration and side reactions and eventually results in battery failure. In addition, the sluggish ion transport kinetics and fragmented SEI in BE lead to a loose and uneven Li deposition layer on the Cu foil, as observed in the cross-sectional SEM images (Fig. S21). However, the deposition layer in BE-TFMB is uniform and compact, further verifying its favorable compatibility with Li metal. *In situ* optical microscopy reveals the dynamic evolution of Li deposition behavior. As shown in Fig. 2f, random dendrite growth emerges in BE within only 15 minutes at 2.5 mA cm^−2^. Impressively, the BE-TFMB system maintains a homogeneous Li deposition morphology over time.

Fig. S22 and S23 illustrate the differences in the SEI composition derived from the two electrolytes. Due to excessive decomposition of the solvents, the BE-derived SEI exhibits a higher organic content, which cannot effectively resist electrolyte penetration and severe side reactions. The depletion of active Li^+^ and the steadily thickened SEI greatly impair the cycling stability of batteries. Conversely, the SEI in BE-TFMB shows a stable LiF signal (Fig. 2h and i), and the elevated LiF content in the inner SEI not only corresponds to the preferential reduction of TFMB but also reflects TFMB's role in promoting the involvement of PF_6_^−^ during SEI formation. Furthermore, this conclusion is corroborated by Li 1s spectra. The BE-induced SEI contains abundant Li_2_CO_3_ and ROCO_2_Li species, which significantly increase interfacial impedance and hinder Li^+^ transport.^28^ Notably, the synergistic effect of B–F, LiF and Li_2_O strengthens the mechanical stability of the interphases, and abundant inorganic grain boundaries provide numerous pathways for Li^+^ transport (Fig. 2j).^29^ As shown in Fig. 2g, time-of-flight secondary ion mass spectrometry (TOF-SIMS) images distinctly present an inorganic-rich inner layer and an organic-rich outer layer in the BE-TFMB-derived SEI. High P–O/P=O contents in the outer layer and remarkable B–O species help enhance the flexibility of the SEI, allowing adaptive accommodation of volume changes and stress accumulation during the cycling process. Unfortunately, the BE-generated SEI exhibits excessive organic aggregation on its surface. The weak and uneven signals of inner inorganic species fail to inhibit uncontrolled Li dendrite growth and parasitic electrolyte reactions (Fig. S24 and S25).

**Fig. 3:**
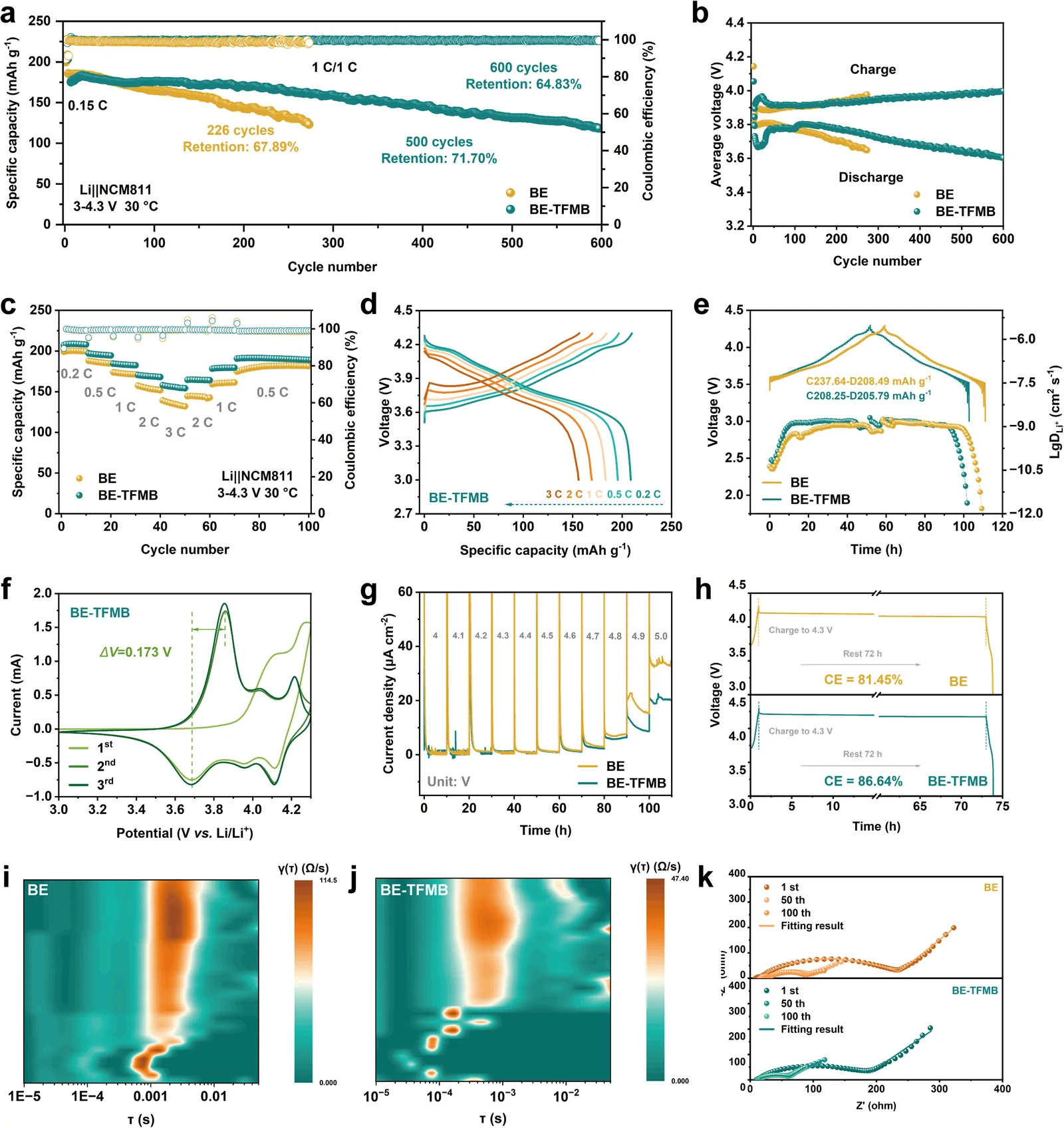
(a) Cycling stability of Li‖NCM811 cells. (b) Average charge/discharge voltage curves. (c) Rate capability of Li‖NCM811 cells using BE and BE-TFMB. (d) Corresponding charge/discharge curves of cells containing BE-TFMB. (e) GITT measurements and corresponding *D*_Li^+^_. (f) CV curves of Li‖NCM811 cells with BE-TFMB. (g) Leaking current curves of Li‖NCM811 cells from 4.0 V to 5.0 V with a time interval of 10 h. (h) Self-discharge tests of Li‖NCM811 cells using two electrolytes. Voltage-dependent DRT analysis of cells with (i) BE and (j) BE-TFMB. (k) Electrochemical impedance spectra (EIS) curves of Li‖NCM811 cells at different cycles.

To fully evaluate the feasibility of the electrolytes, the electrochemical performance of Li‖NCM811 cells was measured. The optimal content of TFMB was explored first. As shown in Fig. S26, compared with cells using electrolytes with different TFMB concentrations (0.5, 1.5, and 2 wt%), the cells using BE with 1 wt% TFMB exhibit the highest discharge capacity and capacity retention. This phenomenon may be attributed to the fact that excessive decomposition of TFMB leads to high interfacial impedance, whereas inadequate additive content fails to participate in stable interphase formation and inhibit excessive solvent decomposition. Moreover, high initial CE confirms that BE-TFMB enables the formation of robust and highly ion-conductive interphases (Fig. S27). Therefore, subsequent analysis focuses on the performance of BE-TFMB. As shown in Fig. 3a, Li‖NCM811 cells using BE struggle to maintain stable cycling and degrade after only 226 cycles. Benefiting from the robust, highly ion-conductive interphases formed by BE-TFMB, cells using BE-TFMB maintain 71.70% capacity retention after 500 cycles, demonstrating good adaptability to high-nickel NCM811 cathodes. Correspondingly, the average charge and discharge voltages of BE-TFMB-based cells show a minor increase during the entire cycling. In contrast, due to increased electrochemical polarization, the average charge and discharge voltages of cells containing BE increase by 0.09 V and 0.15 V after only 273 cycles, respectively (Fig. 3b). Notably, the significant fluctuations during the initial cycling stage may be caused by compositional and structural reorganization of interphases induced by the decomposition of TFMB (Table S3). In addition, the irreversible Li^+^ loss from the formation of interphases and electrochemical reactions of additives and solvents is another important factor.^30^ In addition, the feasibility of the designed electrolyte is further assessed using Li‖NCM811 cells with a high cathode loading (10 mg cm^−2^), Li anode (50 µm) and lean electrolyte (5 g Ah^−1^). As shown in Fig. S28, compared to the cells using BE, the cells with BE-TFMB can provide an initial discharge capacity of 226.25 mAh g^−1^ and cycle stably for 50 cycles with a capacity retention of 96.35%, which verifies the broader applicability of BE-TFMB.

Galvanostatic intermittent titration technique (GITT) measurements were employed to evaluate Li^+^ diffusion kinetics within NCM811 cathodes (Fig. 3e). Compared to Li‖NCM811 cells with BE, cells using BE-TFMB maintain higher diffusion coefficients and lower overpotentials throughout relaxation periods. Rapid ion diffusion kinetics effectively improve the cycling performance of cells at high current densities.^31^ As demonstrated in Fig. 3c, Li‖NCM811 cells with BE are unable to form stable interphases. Continuous electrolyte side reactions hinder BE-based cells from delivering a high and steady capacity under high-rate cycling. Consequently, BE-based cells can only output low specific capacities at 3C, accompanied by rapid capacity fading. Their capacities fail to recover reversibly even when the current densities are reduced. However, BE-TFMB-based cells provide a higher discharge specific capacity (156.36 mAh g^−1^) *versus* 131.85 mAh g^−1^ of BE-based counterparts at 3C. Relevant charge/discharge curves confirm that the cells with BE-TFMB show low overpotentials and stable capacity output (Fig. 3d and S29). Notably, cyclic voltammetry (CV) results in Fig. 3f demonstrate that Li‖NCM811 cells using BE-TFMB possess a smaller difference in redox peak potentials (0.173 V), which is lower than the value of 0.253 V for BE-based cells and reflects rapid Li insertion/extraction kinetics. Additionally, BE-based cells exhibit a distinct oxidation current at 3.6 V, whereas a current rise starts at 3.9 V in BE-TFMB-based cells, which is consistent with LSV data and suggests the high-voltage tolerance of BE-TFMB. The good overlap of the second and third CV curves confirms the structural reversibility of the NCM811 cathode in BE-TFMB, favoring long-term cycling stability (Fig. S30).

While the bifunctional TFMB can alleviate the degradation of NCM811 cathodes, the stability of the resulting CEI still needs further verification. Potentiostatic polarization measurements were employed to evaluate the stability of the CEI at high voltages. The leaking currents of both electrolytes increase with rising voltage, but cells containing BE-TFMB always reach a stable state quickly and show lower current values than those using BE, proving the high-voltage stability of the BE-TFMB-derived CEI (Fig. 3g). Moreover, self-discharge tests in Fig. 3h and S31 demonstrate inferior high-voltage durability of interphases in BE. After being charged to 4.3 V and stored for 72 hours, the open-circuit voltage of cells using BE decreases to 4.14 V, discharging only 81.45% of the initial charge capacity. In comparison, cells using BE-TFMB not only maintain a high voltage (4.17 V) but also deliver a capacity retention of 86.64%. Distribution of relaxation times (DRT) analysis was adopted to track the dynamic evolution of impedance during the initial interphase formation process (Fig. S32). Li‖NCM811 cells with BE-TFMB exhibit shorter relaxation times and reduced impedance throughout the cycling process.^32^ Unfortunately, the unstable BE-derived interphases show high impedance of interphases and charge transfer, which is detrimental to cycling performance at high rates (Fig. 3i and j). The long-term impedance monitoring highlights that BE-TFMB facilitates the formation of thin and highly ion-conductive interphases. As shown in Fig. 3k and Table S3, Li‖NCM811 cells containing BE-TFMB consistently exhibit lower *R*_i_ and *R*_ct_ values compared with cells using BE, which is beneficial for ion transport and charge transfer during the electrochemical process (Fig. S33).

**Fig. 4:**
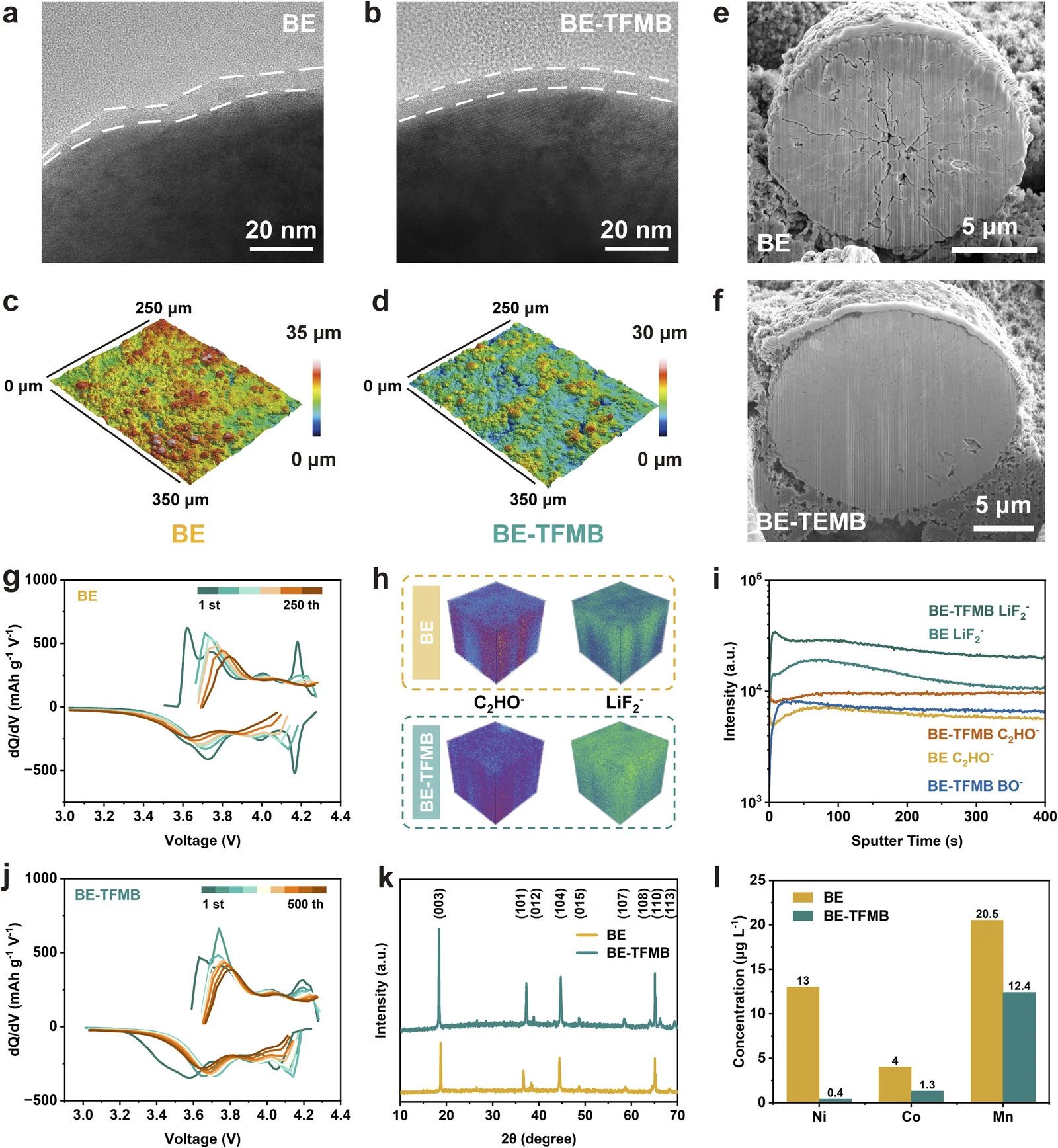
(a and b) TEM, (c and d) CLSM and (e and f) FIB images of cycled NCM811 cathodes in (a, c and e) BE and (b, d and f) BE-TFMB. d*Q*/d*V* curves of Li‖NCM811 cells containing (g) BE and (j) BE-TFMB. (h) 3D rendering images for secondary fragments of C_2_HO^−^, LiF_2_^−^ and BO^−^ in the CEI constructed using BE and BE-TFMB. (i) Corresponding intensity sputter profiles of these fragments. (k) XRD patterns of cycled cathodes from the two electrolytes. (l) The concentrations of TMIs of cycled Li anodes using BE and BE-TFMB.

To explain the reasons for the good compatibility of BE-TFMB with NCM811 cathodes, the regulatory effect of TFMB on the structure and composition of the CEI was examined. SEM images show that the surface of cycled NCM811 cathodes in BE is covered with loose deposits and exhibits obvious cracks (Fig. S34), whereas the cathodes cycled in BE-TFMB show smooth morphology with their primary particles tightly aggregated. Corresponding element distribution mapping analysis detects uniform F and B signals on the cathode after cycling in BE-TFMB, demonstrating that TFMB successfully participates in CEI formation and preserves the structural integrity of cathodes (Fig. S35). Focused ion beam (FIB) analysis was conducted to characterize the internal structure of cycled NCM811 cathodes. BE is unable to form a protective CEI and triggers electrolyte penetration and side reactions, so the distinct cracks on the cathode result from continuous stress accumulation and structural collapse during cycling (Fig. 4e). Remarkably, the stable and high-voltage-resistant CEI induced by BE-TFMB efficiently protects the structure of cathodes, with no cracks appearing after prolonged cycling (Fig. 4f). Furthermore, confocal laser scanning microscope (CLSM) images in Fig. 4c and d reveal that cycled cathodes display more pronounced surface undulations, confirming severe structural fragmentation in BE. However, the cathodes after cycling in BE-TFMB present a relatively smooth and uniform surface. Even over a larger detection area, cathodes obtained in BE-TFMB still demonstrate low surface roughness, further validating that TFMB can form an intact and uniform CEI and maintain the structure of cathodes (Fig. S36). Transmission electron microscopy (TEM) characterization in Fig. 4b reveals detailed CEI morphology in the two electrolytes. The CEI derived from BE-TFMB is only ∼6 nm thick and is distributed continuously and uniformly on the cathode surface. In contrast, uncontrolled electrolyte decomposition and sustained cathode structural damage in BE lead to a fractured CEI, while the excessively thick CEI extends the Li^+^ transport path and raises interphase impedance simultaneously (Fig. 4a).

The chemical composition of the CEI was elucidated using X-ray photoelectron spectroscopy (XPS). Fig. S37 presents strong M–O signals on the surface of the CEI in BE, indicating continuous structural degradation of NCM811 cathodes and severe dissolution of TMIs. By comparison, weaker M–O signals are detected on the surface of the CEI in BE-TFMB, while inner-layer signals gradually increase as the etching depth increases. This result suggests that the BE-TFMB-derived CEI is thin, which is consistent with the TEM conclusions. Furthermore, as an anion receptor, TFMB facilitates the formation of a highly ion-conductive CEI enriched with B, F, and P species. Therefore, the CEI derived from BE-TFMB contains a high content of LiF and B–F components. Such inorganic-dominated interphases can mitigate harmful reactions at high voltage, thus preventing cathode corrosion and enhancing the cycling stability of cells (Fig. S38).^33^ Notably, cycled cathodes collected from BE show a more pronounced Ni^2+^ peak, which demonstrates a high Li/Ni mixing degree. Consequently, the unstable CEI cannot ensure the integrity of cathodes and the cathodes in BE suffer drastic structural damage. Subsequent TOF-SIMS measurements further demonstrate the inorganic-rich characteristic of the CEI in BE-TFMB. As depicted in Fig. 4h and i, the CEI in BE-TFMB displays high intensity and homogeneous distribution of LiF_2_^−^, whereas the BE-derived CEI shows intense C_2_HO^−^ signals and disordered distribution of both organic and inorganic species. The high LiF_2_^−^ content in the BE-TFMB-based CEI originates not only from the PF_6_^−^ anions but also from the involvement of –CF_3_ groups in TFMB (Fig. S39). Importantly, the depth intensity profiles reveal stable B–O signals throughout the BE-TFMB-derived CEI, confirming the key role of TFMB in constructing robust and highly ion-conductive interphases (Fig. S40 and S41).^34^

The electron-deficient B atoms of TFMB can coordinate with electron-rich groups to eliminate residual alkali substances on the cathode surface, thereby enhancing the stability of the CEI and NCM811 cathodes.^35^ The d*Q*/d*V* curves in Fig. 4g suggest that the structural degradation of the NCM811 cathode is alleviated in BE-TFMB. However, the curves of the cycled NCM811 cathode in BE show poor overlap, indicating inferior structural stability and irreversible phase transitions (Fig. 4j). Additionally, X-ray diffraction (XRD) patterns demonstrate strong diffraction peak intensities for the cycled cathode in BE-TFMB. Its higher *I*_(003)_/*I*_(104)_ value of 1.42 indicates a lower degree of Li/Ni mixing relative to the cathode in BE (1.38), consistent with Ni 2p XPS results (Fig. 4k).^36^ Inductively coupled plasma mass spectrometry (ICP-MS) tests were performed to assess the dissolution and deposition of TMIs on Li anodes after cycling. The lower content of Ni, Co, and Mn on the anode surface in BE-TFMB verifies the protective effect of the TFMB-regulated CEI on cathodes (Fig. 4l). Overall, TFMB participates in the formation of a robust and highly ion-conductive CEI, thereby inhibiting irreversible structural transformations of NCM811 cathodes, dissolution of TMIs, and electrolyte side reactions during long-term cycling.

**Fig. 5:**
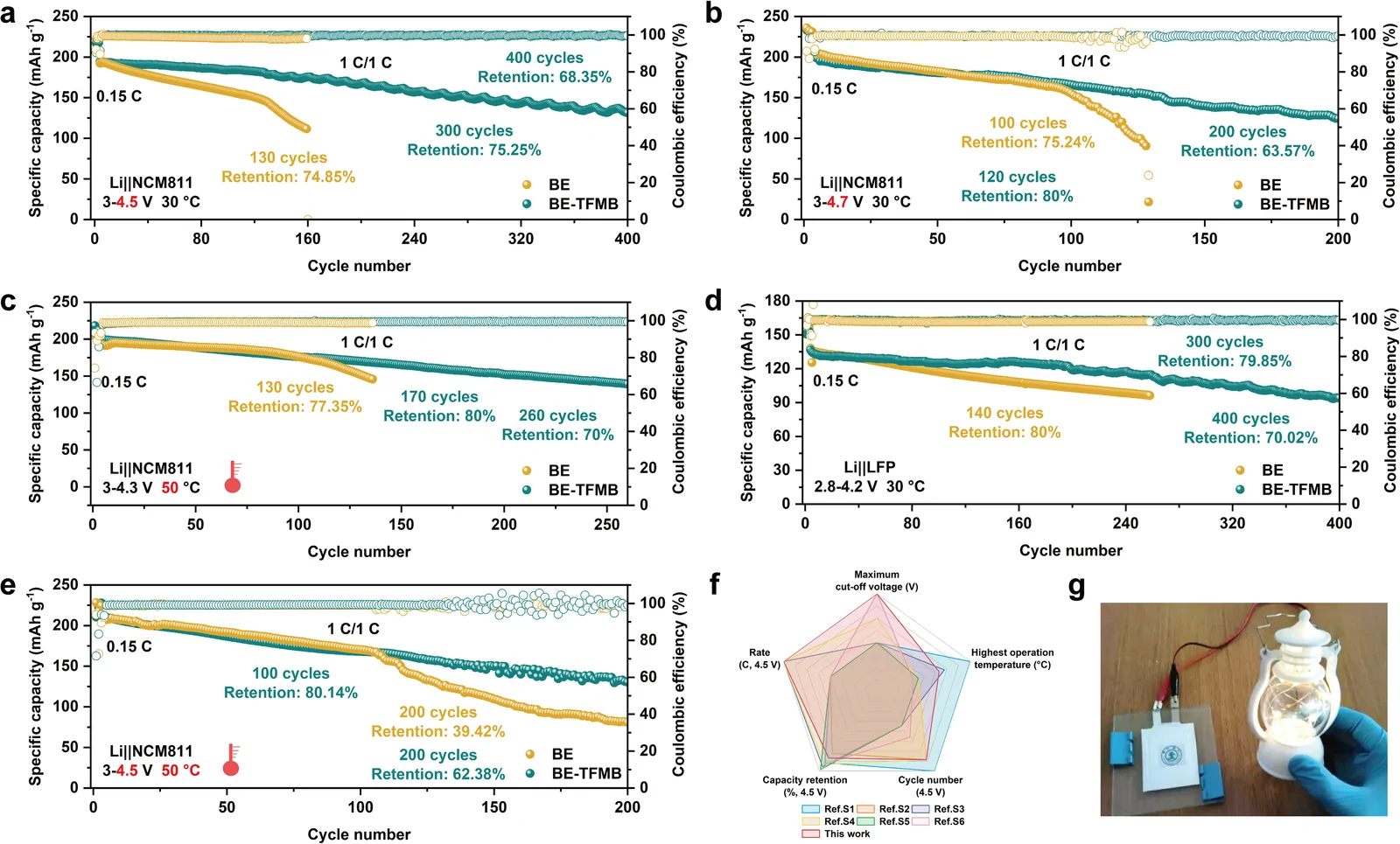
Cycling performance of Li‖NCM811 cells at a charge/discharge rate of 1C between (a) 3 and 4.5 V and (b) 3 and 4.7 V. Cycling performance of Li‖NCM811 cells under 50 °C at 1C within voltage ranges of (c) 3–4.3 V and (e) 3–4.5 V. (d) Cycling performance of Li‖LFP cells at 1C within a voltage range of 2.8–4.2 V. (f) Radar plots of BE-TFMB and reported typical electrolytes in terms of cutoff voltage, rate, cycle number, capacity retention, and temperature. (g) Optical images of LEDs powered by the Li‖NCM811 pouch cell using BE-TFMB.

Given that TFMB facilitates the formation of the protective and highly ion-conductive interphases to prevent sustained electrolyte decomposition and ensure the structural integrity of the electrodes, the high-voltage tolerance of Li‖NCM811 cells using BE-TFMB was further investigated. As displayed in Fig. 5a, Li‖NCM811 cells using BE-TFMB realize stable cycling over 400 cycles and maintain 68.35% of their initial capacity at a high voltage of 4.5 V and 1C current density. However, the violent side reactions in BE induce rapid cell failure within 130 cycles (Fig. S42). Surprisingly, even at an ultra-high voltage of 4.7 V, BE-TFMB enables Li‖NCM811 cells to achieve stable cycling for 200 cycles, accompanied by a high specific capacity of 200.15 mAh g^−1^ and a capacity retention of 63.57% (Fig. 5b). The charge/discharge curves verify the alleviated electrochemical polarization and stable discharge plateau in the BE-TFMB system (Fig. S43). As expected, BE-containing cells can only complete 100 cycles and exhibit obvious capacity fading. High temperatures accelerate the electrochemical degradation of conventional carbonate electrolytes, phase transitions of the NCM811 cathode, and dissolution of TMIs. Thus, Li‖NCM811 cells using BE struggle to maintain stable operation and suffer rapid performance degradation at 50 °C (Fig. 5c). The dual protection functions of TFMB on both the cathode and anode enable BE-TFMB-based cells to deliver stable capacity output at high temperatures. Furthermore, even at 50 °C and an elevated charging voltage of 4.5 V, BE-TFMB still allows the cells to operate normally, achieving excellent stability with 80.14% of the initial capacity retained after 100 cycles (Fig. 5e).

Investigating the failure mechanisms of BE-based cells under high-temperature and high-voltage environments helps clarify how TFMB enhances performance. First, the unstable interphases induce continuous electrolyte penetration and side reactions under harsh conditions and struggle to maintain electrode integrity, ultimately inducing severe fragmentation of the NCM811 cathode and dissolution of TMIs (Fig. S44–S47). Meanwhile, Fig. S48–S53 show that the growth of Li dendrites raises safety concerns for the cells. On the other hand, the intrinsic LiPF_6_ hydrolysis in BE is further exacerbated under extreme operating conditions, and the resulting products corrode the electrodes and the interphases. Notably, the interaction between TFMB and PF_6_^−^ not only facilitates the involvement of anions in the formation of the interphases but also suppresses the hydrolysis of LiPF_6_ at high temperatures. Thus, the stable and inorganic-rich interphases in BE-TFMB enable the reversible cycling of Li‖NCM811 cells under harsh high-temperature and high-voltage conditions.

Notably, the broad cathode compatibility of BE-TFMB is validated in Li‖LFP cells. As shown in Fig. 5d, Li‖LFP cells with BE-TFMB achieve a high reversible capacity over 400 cycles, confirming the universality of TFMB's synergistic protective effects on electrodes. Although BE ensures stable cycling of Li‖LFP cells within 280 cycles, uncontrollable side reactions and increased impedance severely limit its long-term application in LFP-based cells (Fig. S54). Additionally, a Li‖NCM811 pouch cell assembled with BE-TFMB can successfully power LED lights, validating the potential practicality of the optimized electrolyte (Fig. 5g). As summarized and compared in Fig. 5f and Table S4, a comprehensive evaluation covers cut-off voltage, cycle life, capacity retention, operating temperature, and charge/discharge rate. It is evident that BE-TFMB achieves balanced overall performance and demonstrates great application prospects for high-temperature and high-voltage LMBs.^9,36–41^

## Conclusion

In this work, a TFMB-regulated electrolyte is designed to form stable interphases and mitigate the structural deterioration of electrodes, thereby achieving stable operation of Li‖NCM811 cells under extremely high temperature and high voltage conditions. Specifically, TFMB reacts prior to solvent molecules and preferentially participates in the formation of compact interphases. Furthermore, the electron-deficient B sites in TFMB can coordinate with PF_6_^−^ anions, thereby effectively improving the ionic conductivity of BE-TFMB and restraining the hydrolysis of LiPF_6_. Crucially, B- and F-rich interphases can mitigate harmful side reactions under harsh conditions, which suppresses irreversible phase transitions of the cathode, the deposition of TMIs and the uncontrolled growth of Li dendrites on the anode surface. As a result, Li‖NCM811 cells assembled with BE-TFMB exhibit excellent cycling stability at 4.5 V, maintaining 75.25% of their initial capacity after 300 cycles. Furthermore, the cells can complete 120 cycles at 1C even under an ultra-high cut-off voltage of 4.7 V with a capacity retention of 80%. Notably, the excellent cycling lifespan with 80.14% capacity retention over 100 cycles at 50 °C and 4.5 V further validates the promising application prospects of BE-TFMB. Its excellent compatibility with LFP cathodes also verifies the broad adaptability toward various cathode materials. In conclusion, this work establishes the relationship between additive design and the derived interphases, offering new guidance for the exploration of high-temperature and high-voltage electrolytes for LMBs.

## Author contributions

Yanan Li: writing – original draft, visualization, software, methodology, investigation, formal analysis, data curation, and conceptualization. Wenzhe Zhang: data curation. Xiaosha Wu: data curation. Rui Yu: formal analysis. Kun Wang: data curation. Kai Xi: supervision, conceptualization, and writing – review & editing. Shujiang Ding: funding acquisition, writing – review & editing, and visualization. Guoxin Gao: writing – review & editing and visualization.

## Conflicts of interest

The authors declare that they have no known competing financial interests or personal relationships that could have appeared to influence the work reported in this paper.

## Supplementary Material

SC-017-D6SC04807B-s001

## Data Availability

The data supporting this article have been included as part of the supplementary information (SI). Supplementary information is available. See DOI: https://doi.org/10.1039/d6sc04807b.
