# Supplementary material for: Electrolyte additive engineering to construct stable interphases with high ionic conductivity for high-temperature and high-voltage lithium metal batteries
Source: Chem Sci. 2026 Jul 17;17(35):16818–29. doi: 10.1039/d6sc04807b (PMC13392756; doi:10.1039/d6sc04807b)
Supplement: Supplementary file 1 [file SC-017-D6SC04807B-s001.pdf]

Supplementary Information (SI) for Chemical Science.
This journal is © The Royal Society of Chemistry 2026

Supporting Information

Electrolyte Additive Engineering to construct stable interphase with high ionic conductivity for high-temperature and high-voltage lithium metal batteries

Yanan Li†, Wenzhe Zhang†, Xiaosha Wu, Rui Yu, Kun Wang, Kai Xi*, Shujiang Ding*, Guoxin Gao*

School of Chemistry, Engineering Research Center of Energy Storage Materials and Devices, Ministry of Education, National Innovation Platform (Center) for Industry-Education Integration of Energy Storage Technology, Xi'an Jiaotong University, Xi'an 710049, P.R. China

† These authors contributed equally.

Corresponding authors:

kx210.cam@xjtu.edu.cn (K. Xi)

dingsj@mail.xjtu.edu.cn (S. Ding)

gaoguoxin@mail.xjtu.edu.cn (G. Gao)

Experimental Sections

Preparation of materials and electrolytes

4-trifluoromethylbenzeneboronic acid neopentyl glycol ester (TFMB, 98%) were obtained from Macklin Biochemical Technology Co., Ltd. The LB008 electrolyte (1.0 M $LiPF_6$ in EC/DEC, v/v = 1:1) was provided by Dodo Chem., Suzhou, China. $LiNi_{0.8}Co_{0.1}Mn_{0.1}O_2$ (NCM811) cathodes were provided by Li-FUN Technology (Hunan, China). $LiFePO_4$ (LFP), conductive carbon black (Super-P-Li) and poly(vinylidene fluoride) (PVDF, HSV900) were gained from Canrd Technology Co. Ltd. N-Methyl pyrrolidone (NMP, 99.5%) were purchased from Aladdin. Lithium foil with 600 μm in thickness (diameter: 16 mm and 12 mm) were purchased from Tianjin Nengli Co., Ltd. Deuterated reagents were purchased from Energy Chemical Co., Ltd. The separator used in this work is Celgard 2325.

For the convenience of comparison, the LB008 electrolyte was denoted as baseline electrolyte (BE). The BE electrolyte containing 0.5, 1.0, 1.5, 2.0 wt% of TFMB additive was functional electrolyte. Specifically, BE containing 1.0 wt% of TFMB is termed BE-TFMB for subsequent discussion. To clearly identify the source of LiF_2^- in BE-TFMB-derived SEI, the 1.0 M LiCl was dissolved in a mixture of EC and DEC (v/v = 1:1) to prepare a reference electrolyte, designated as BE-Cl. All electrolytes were stirred for 8 h at room temperature and then stored in an Ar-filled glove box (O_2 < 0.1 ppm, H_2O < 0.1 ppm).

Electrode and battery assembly

For the preparation of the cathode, 80 wt.% of active materials (NCM811, LFP), 10 wt.% PVDF and 10 wt.% Super P-Li were dispersed in NMP to form a homogeneous slurry. Then the slurry was coated on Al foil uniformly and dried in a vacuum oven at 120 °C overnight. Finally, the work electrodes with mass loading of 3 mg cm^{-2} were cut into wafers (diameter: 12 mm). Coin-type cells were assembled by using Li foil as anode (diameter: 14 mm, thickness: 600 μm) and NCM811 (or LFP) as cathode (diameter: 12 mm). Li foils (diameter: 12 mm, thickness: 600 μm) and Cu foils (diameter: 14 mm) were employed to assemble the symmetric Li||Li cells and asymmetric Li||Cu cells. The anode and cathode were separated by a Celgard 2325 membrane and injected electrolytes with 50 μL. The applicability of electrolytes was evaluated by assembled Li||NCM811 cells with increased cathode loading (10 mg cm^{-2}), thinner Li anode (50 μm), and lean electrolyte (5 g Ah^{-1}). All electrochemical tests were characterized by applying 2032-type coin-cells from Canrd Technology Co. Ltd.

Electrochemical measurements

The electrochemical performance of all cells was collected by using NEWARE Battery Test System (CT-4008T-5V50mA-164, Shenzhen, China). For all performance tests, the cells were first rested for 8 h to ensure complete wetting of the anode and cathode by electrolytes. The Li||NCM811 cells were cycled between 3-4.3, 3-4.5, and 3-4.7 V (vs. Li^+/Li) at 1 C (1 C = 200 mA g^{-1}). Li||LFP cells were cycled with the voltage window of 2.8-4.2 V (vs. Li^+/Li) at 1C (1 C = 170 mA g^{-1}). Before the measurement of electrochemical performance, 0.15 C was selected for the initial activation to induce stable interphase in cells. The activation process was carried out at 30 or 50 °C for 3 cycles and then cycled at selected rate in a temperature-controlled box at 30 or 50 °C.

The Li||Li cells were cycled at 0.5 mA cm^{-2} with a total capacity of 0.5 mAh cm^{-2} at 30 °C.

For the Aurbach's CE test, a protocol was designed as follows: (1) performed one initial formation cycle with Li metal deposition of 4 mAh cm^{-2} on Cu with 0.5 mA cm^{-2} and stripping Li to 1 V; (2) deposited 4 mAh cm^{-2} Li on Cu with 0.5 mA cm^{-2} as a Li reservoir; (3) repeatedly stripped/deposited Li of 1 mAh cm^{-2} with 0.5 mA cm^{-2} for 20 cycles; (4) stripped all Li to l V. Then the CE could be calculated via the following Eq (1).

$$CE = \frac{nQ_C + Q_s}{nQ_c + Q_T} \quad (1)$$

where, Q_T is a given amount of charge (depositing Li onto the Cu substrate first as a Li reservoir), Q_c is a smaller portion of this charge (cycling Li between working and counter electrodes for n cycles), and Q_S is the final stripping charge (the quantity of Li remaining after cycling).

The exchange current (i_0), is performing Li||Li cells with Galvanostatic charge/discharge at various currents from 40 to 140 µA to extract overpotential and calculate using the Butler-Volmer Equation (Eq(2)).

$$i \approx i_0 \frac{F\eta}{RT2} \quad (2)$$

where, η is the total overpotential, T is the absolute temperature, R is the gas constant, and F is the faraday constant.

The dQ/dV curves were obtained by differentiating the voltage (V) versus capacity (Q) curves of Li||NCM811 cells using different electrolytes.

Leaking currents of the Li||NCM811 cells were tested at different voltages after activation cycles.

For the galvanostatic intermittent titration technique (GITT) measurements, after 1 cycle at 0.1 C, the Li||NCM811 cells were cycled at a low rate of 0.1 C for 10 min followed by standing for 40 min. The Li^+ diffusion coefficients (D_{Li^+}) were calculated according to the GITT curves.

Self-discharge tests were conducted after the Li||NCM811 cells being charged to 4.3 V and rested for 72 h in

different electrolytes.

Following electrochemical data were drawn from CHI600E electrochemical workstation (Chenhua, Shanghai).

The ion conductivity (σ) of the electrolytes was obtained by constructing symmetric SS||SS cells based on the following Eq (3). Two stainless steel electrodes (diameter: 16 mm) were symmetrically positioned between a polytetrafluoroethylene disc.

$$\sigma = \frac{L}{RS} \qquad (3)$$

where, R is the Ohmic resistance, L is the thickness of the polytetrafluoroethylene disc, S is the area of polytetrafluoroethylene disc.

The Li^+ transference number ($t_{Li}{}^+$) was measured by Li||Li cells with a polarization potential of 10 mV according to the below Eq (4).

$$t_{Li}^{+} = \frac{I_S(\Delta V - I_0 R_0)}{I_0(\Delta V - I_S R_S)} \qquad (4)$$

where, ΔV is the applied potential, R_0 and R_s are initial-state and steady-state resistance. I_0 and I_s are the initial state and steady-state current, respectively.

Tafel plots were tested from −0.3 V to 0.3 V (vs. Li^+/Li) in symmetric Li||Li cells at a scan rate of 1 mV s^{-1} and then managed for linear fitting.

Linear sweep voltammetry (LSV) was operated by applying Li||SS cells at a scan rate of 1 mV s^{-1} from 2.5 V to 5.5 V and Li||Cu cells at a scan rate of 1 mV s^{-1} from 3.0 V to 0 V.

Cyclic voltammetry (CV) was performed by testing Li||NCM811 and Li||Cu cells at a scan rate of 0.2 and 1 mV s^{-1} from 3.0 to 4.3 V and −0.1 to 0.4 V.

Electrochemical impedance spectra (EIS) of the Li||NCM811 cells at different cycles were examined from 10^6 Hz to 10^{-2} Hz.

The in-situ EIS were obtained from the 0.1 C constant current charge/discharge process of the Li||NCM811 cells. Distribution of relaxation times (DRT) of these impedance data were generated using DRT tools to analyze the electrode kinetics at different time scales.

The activation energies of desolvation were obtained from the assembled symmetric Li||Li cells using different electrolytes at various temperatures. Based on the Arrhenius' equation, the E_{ct} of Li||Li cells can be calculated according to the following Eq (5).

$$\frac{1}{R} = Aexp(-\frac{E}{RT}) \qquad (5)$$

where, T is the absolute temperature, A is the pre-exponential constant, R is R_{ct}, E is the activation energy of E_{ct}, R is the standard gas constant.

Characterizations

Raman spectra of the electrolytes were obtained from laser Raman spectrometer (InVia Qontor) with an emission wavelength of 785 nm laser.

The temperature-dependent Raman spectra were collected: BE and BE-TFMB were sealed in separate capillary tube and heated from 20 °C to 60 °C with an interval of 5 °C, and then held at 60 °C for 1 h. At the same time, Raman spectra were continuously acquired at different temperatures and at various time points (5 min intervals) during the isothermal holding period to track the evolution of the characteristic peak of PF_6^- anions.

Fourier transform infrared (FT-IR) spectra of the electrolytes were gained from infrared spectrometer (VERTEX70).

The wettability of the electrolytes on the Celgard 2325 separator and NCM811 cathode was reflected by the optical contact angle meter (DSA100).

NMR spectra of the electrolytes were obtained from the Nuclear Magnetic Resonance Spectrometer (NMR, AVANCE NEO, 400M Bruker).

The time-dependent ^{19}F spectra at 60 °C were obtained: BE and BE-TFMB were sealed in glass bottles, respectively, and heated and stirred at a high temperature (60 °C) with gradient storage times (e.g., 4, 8, and 12 days). At each time point, equal volumes of electrolyte samples were collected, and the HF content in the electrolytes was quantitatively determined using ^{19}F NMR spectra (with trifluoroacetic acid (TFA) as the internal standard). The formula for calculating the HF content is as follows:

$$\frac{A_{TFA}}{N_{TFA}}:\frac{A_{HF}}{N_{HF}} = n_{TFA}:n_{HF} \qquad (6)$$

$$c_{HF} = \frac{n_{HF}}{V_e} \qquad (7)$$

where, A_{TFA} and A_{HF} represent the NMR integral areas of TFA and HF, respectively; N_{TFA} and N_{HF} represent the equivalent numbers of F atoms in TFA and HF, respectively; n_{TFA} and n_{HF} represent the moles of TFA and HF, respectively; V_e represents the volume of the tested electrolyte; and c_{HF} represents the concentration of HF.

The crystal structure of cycled electrode was obtained from X-ray diffraction meter with a Cu-Kα X-ray radiation source (XRD, Bruker D8 ADVANC).

In-situ optical microscope was performed by the Yuescope YM710R microscope under a charging current density of 2.5 mA cm^{-2}.

The morphology and surface roughness of cycled NCM811 cathode were determined by Leica TCS SP8 STED 3X confocal laser scanning microscope (CLSM).

Morphology of the cycled NCM811 cells were observed from field emission scanning electron microscopy (FESEM, Gemini SEM 500) and transmission electron microscope (TEM, Talos F200X).

Focused ion beam (FIB, Thermo Scientific Helios 5 UX) was used to examine the inner cross-section of the NCM811 particles.

The surface information and chemical composition of the electrodes at different depths were conducted via X-ray photoelectron spectra (XPS, Thermo Fisher Scientific, ESCALAB Xi+).

The Small Angle X-ray Scattering (SAXS) measurements were performed at Anton Paar SAXSpoint 2.0 SAXS experimental facility (Austria) equipped with a microfocus Cu X-ray source.

Three-dimensional (3D) tomographic images of the NCM811 cathode and Li anode surface after cycling in different electrolytes were obtained by time-of-flight secondary ion mass spectrometry (TOF-SIMS, M6).

The cycled Li anodes were dissolved by H_2O and then solution was diluted and analyzed using an Inductively Coupled Plasma Mass Spectrometer (ICP-MS, PerkinElmer NexION 350) to quantify the deteriorations of the cathodes.

Theoretical Calculation method

The binding energies between Li^+/PF_6^- and EC, DEC, TFMB, as well as highest occupied molecular orbital (HOMO), lowest unoccupied molecular orbital (LUMO), and electrostatic potentials (ESP) of EC, DEC, TFMB and $LiPF_6$ were respectively calculated by Dmol3 module in Materials Studio (MS) 2023. Firstly, the generalized gradient approximation (GGA) with Perdew–Burke–Ernzerhof (PBE) exchange-correlation functional was employed to fully relax EC, DEC, TFMB and $LiPF_6$. The double-numeric quality basis sets with polarization functions were used. The iterative tolerances for energy change, force and displacements were 1×10^{-5} Ha, 0.002 Ha $Å^{-1}$ and 0.005 Å, respectively. In the self-consistent field (SCF) procedure, 10^{-6} a.u. was used for the convergence standard electron density. After structure optimization, the Adsorption Locator Tools in MS were used to locate Li^+ at energy favorable site of EC, DEC and TFMB.[1] Then each interaction pair was freely optimized by Dmol3 module. Finally,

single point energy calculation was executed, meanwhile, the ESP was output. The binding energies E_b were calculated according to the following Eq (8).[2-3]:

$$E_b = E_{total} - E_{Li^+} - E_{molecule} \qquad (8)$$

Where E_{total} is the total energy of the optimized complex structure, E_{Li+} is the energy of Li^+ ion, $E_{molecule}$ is the energy of EC, DEC and TFMB molecule.

Figures and tables

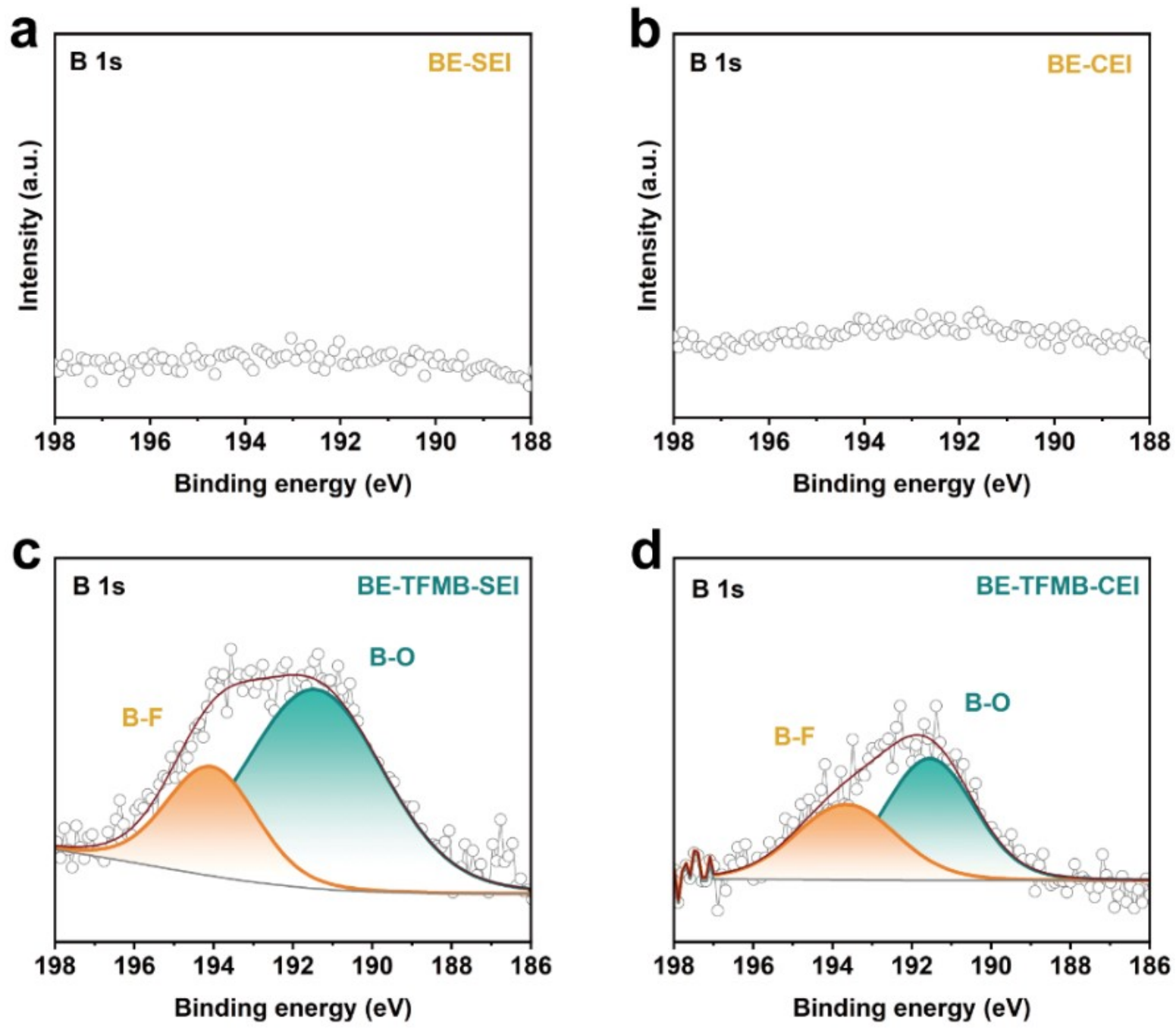

Figure S1. XPS spectra of B *1s* for the (a, c) SEI and (b, d) CEI formed in (a, b) BE and (c, d) BE-TFMB after the initial cycling.

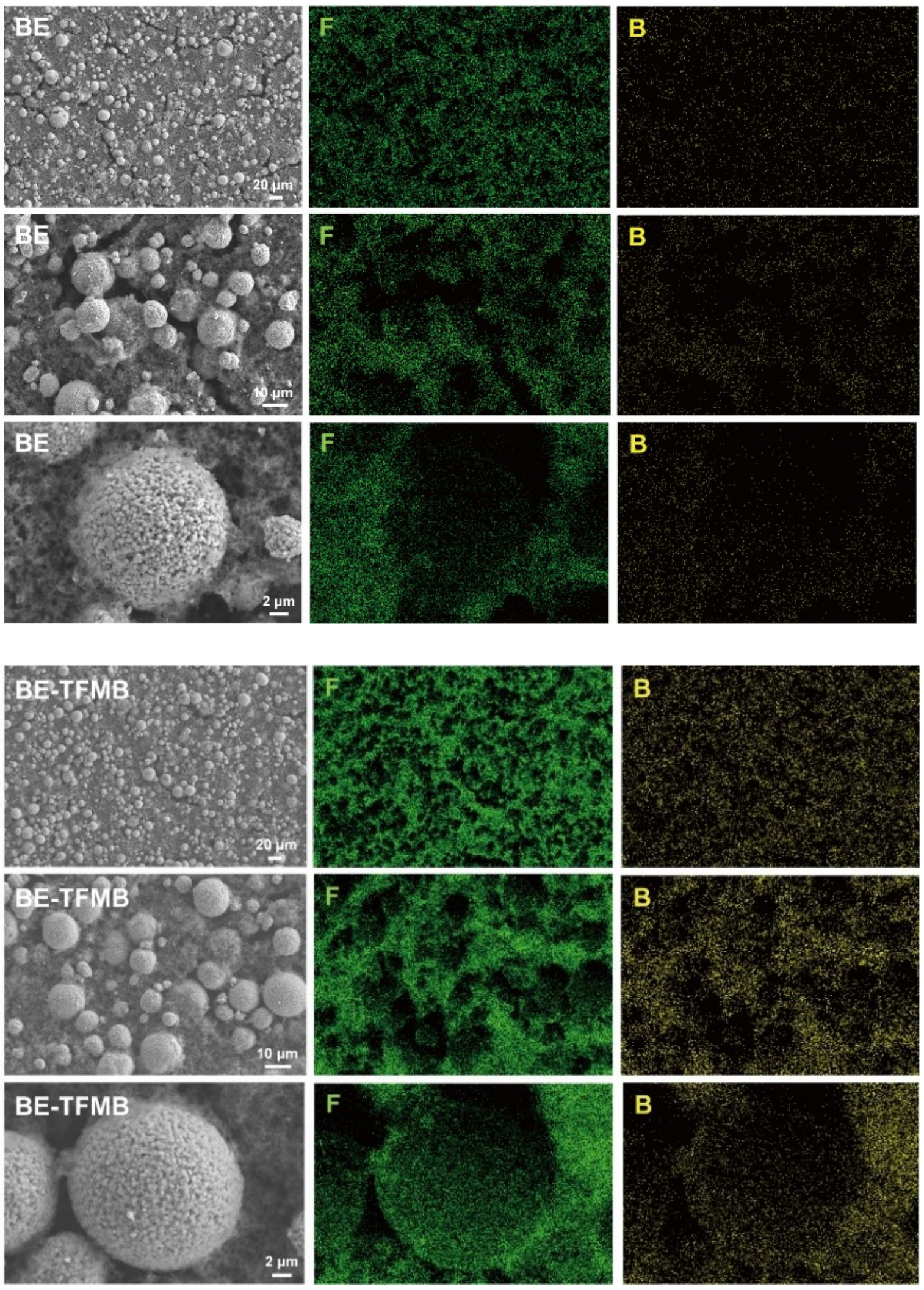

Figure S2. SEM images and corresponding EDS mapping of the cycled NCM811 cathodes in BE and BE-TFMB.

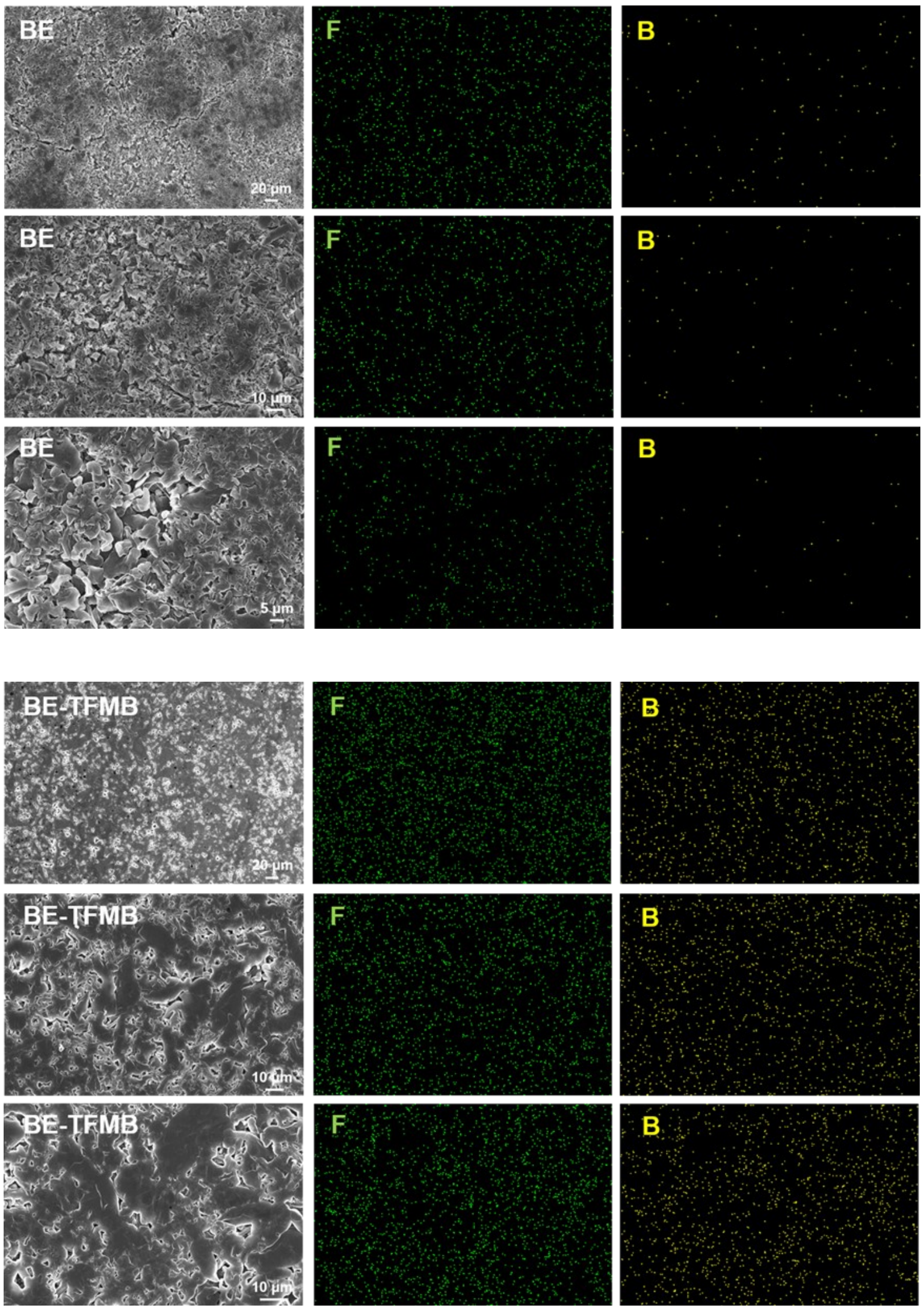

Figure S3. SEM images and corresponding EDS mapping of the cycled Li anodes in BE and BE-TFMB.

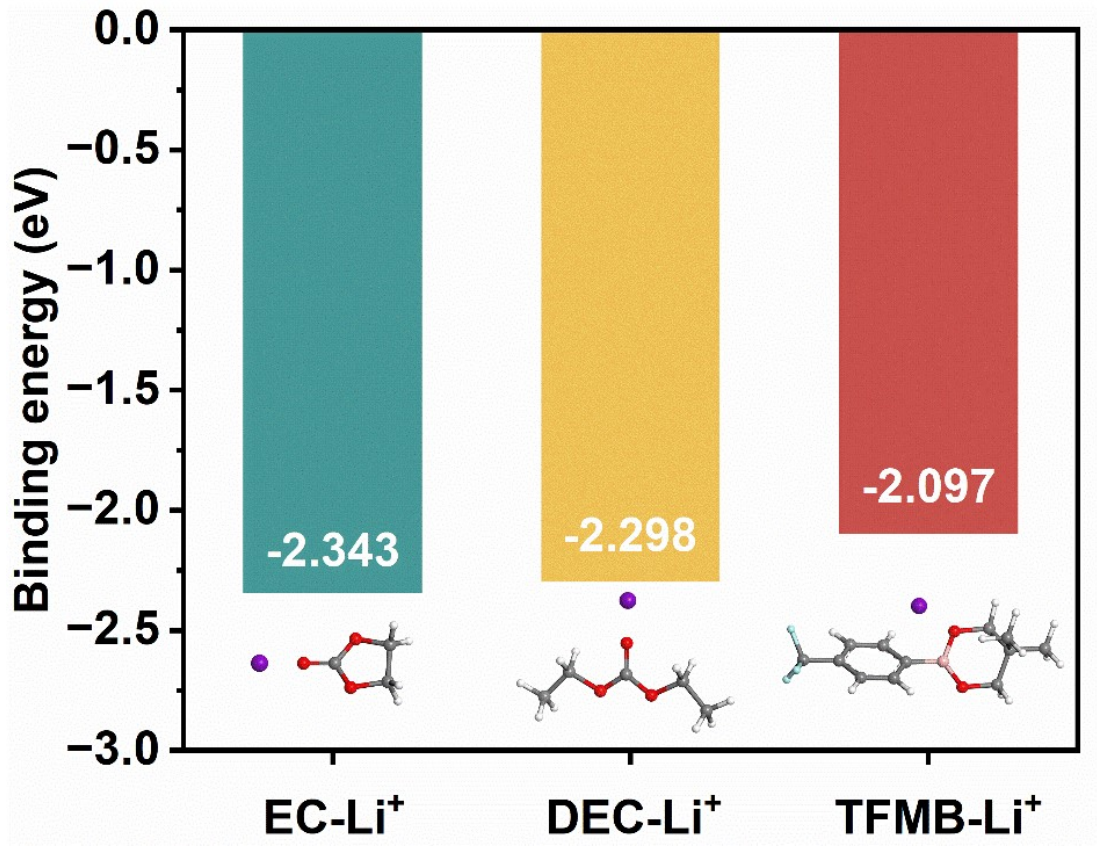

Figure S4. Binding energies of EC-Li^+, DEC-$Li^+$and TFMB-Li^+.

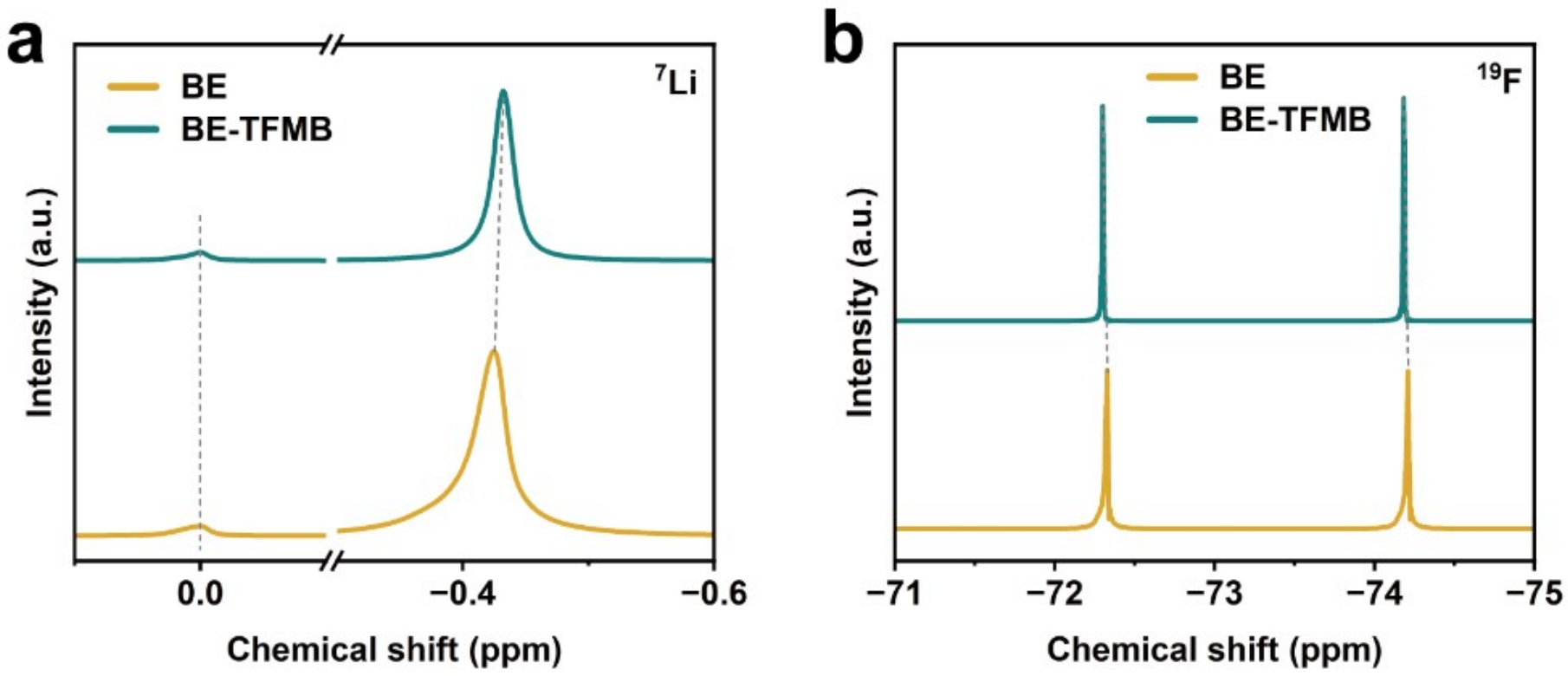

Figure S5. NMR spectra of (a) ^{7}Li and (b) ^{19}F in BE and BE-TFMB.

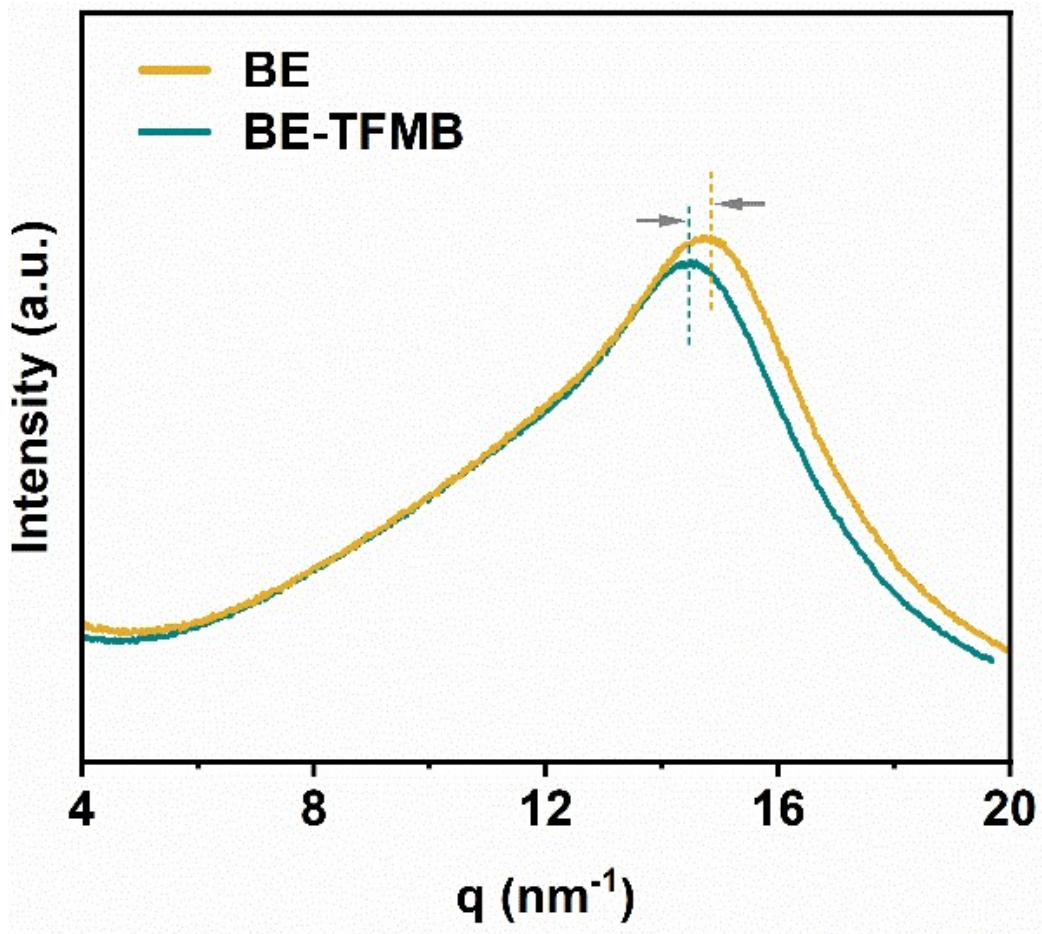

Figure S6. The SAXS data of BE and BE-TFMB.

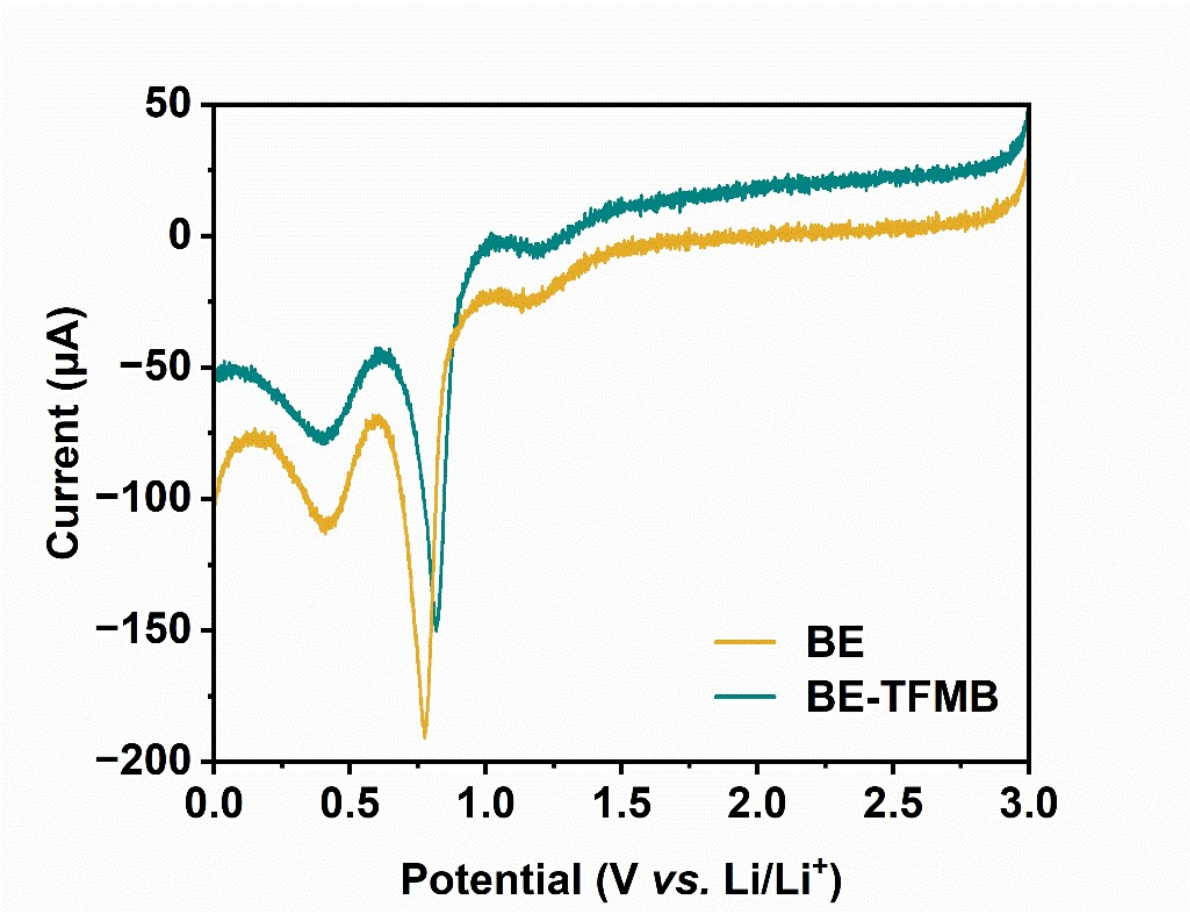

Figure S7. LSV curves of BE and BE-TFMB from 3 V to 0 V.

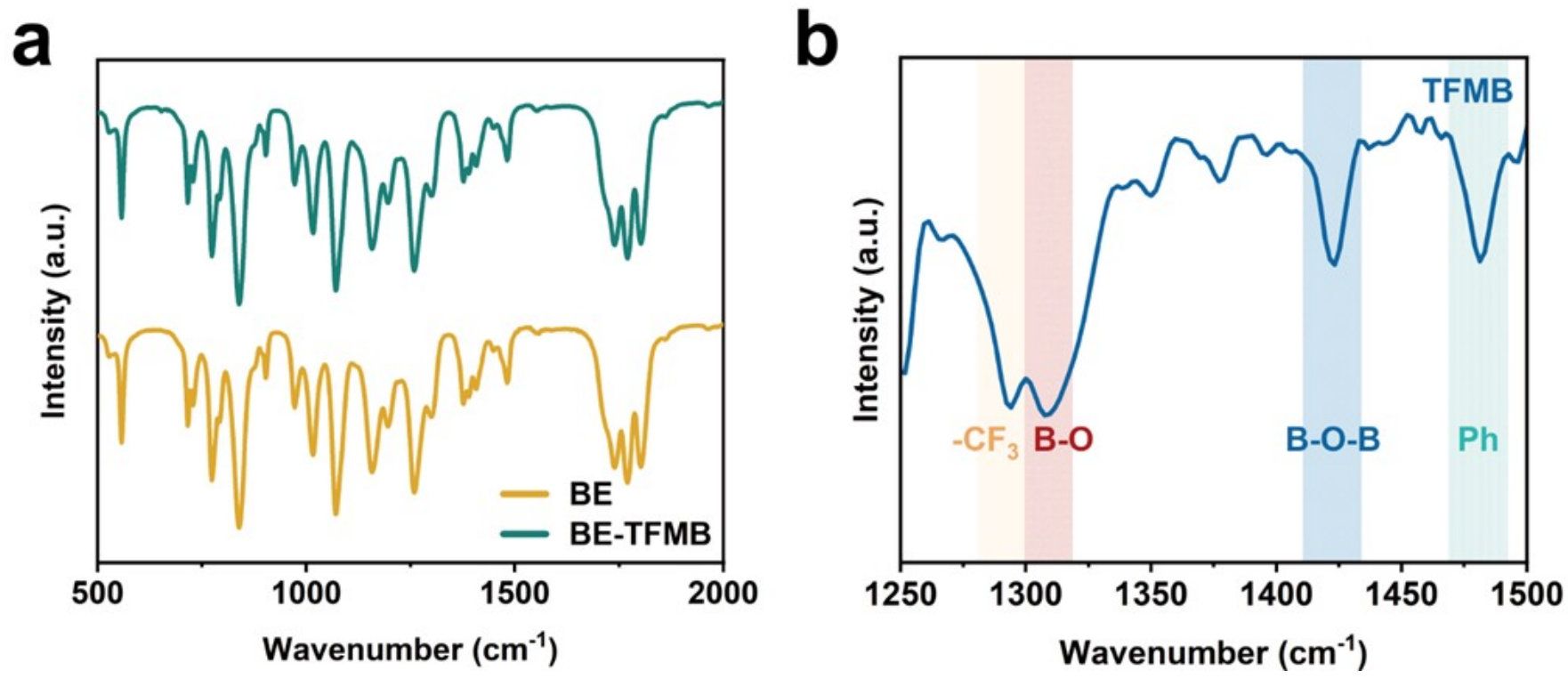

Figure S8. The FT-IR spectra of (a) BE and BE-TFMB at 500-2000 cm^{-1} and (b) TFMB at 1250-1500 cm^{-1}.

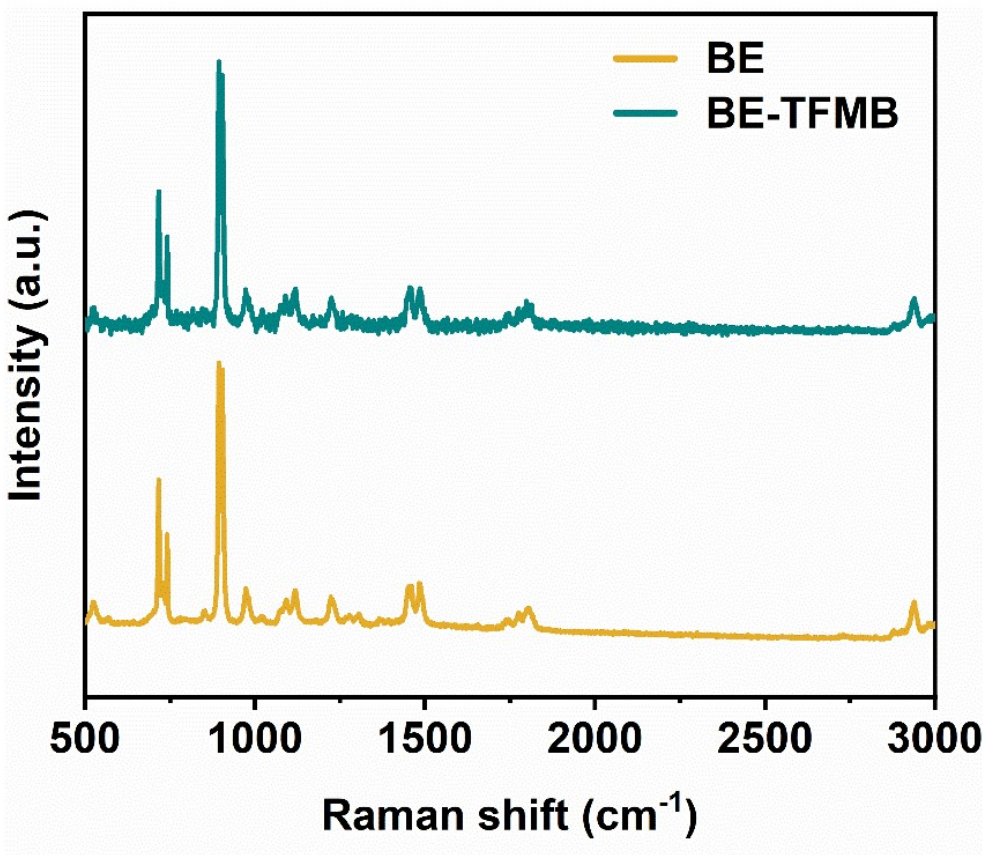

Figure S9. The Raman spectra of BE and BE-TFMB at 500-3000 cm^{-1}.

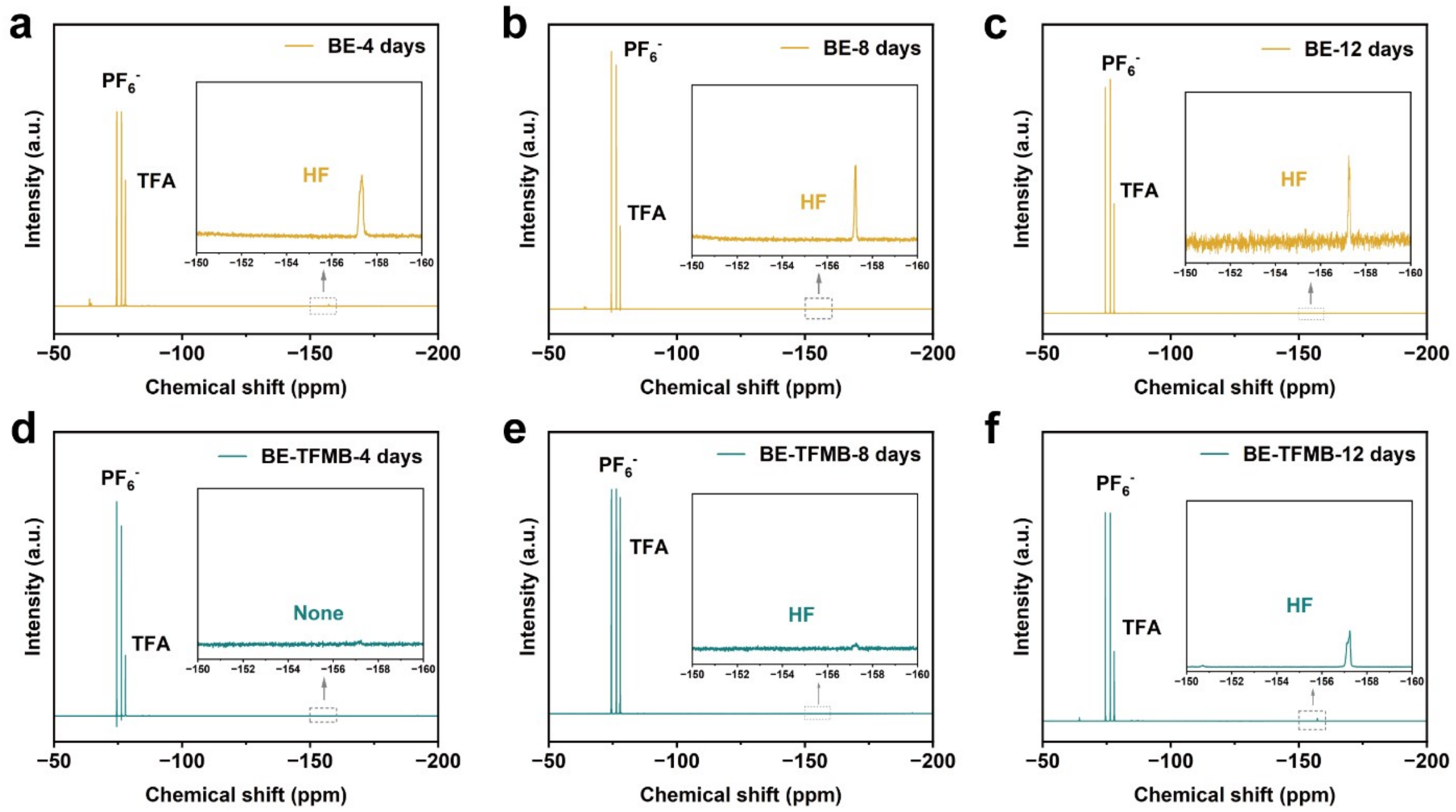

Figure S10. NMR spectra of ^{19}F in (a, b, c) BE and (d, e, f) BE-TFMB after (a, d) 4, (b, e) 8, and (c, f) 12 days of storage at 60 °C.

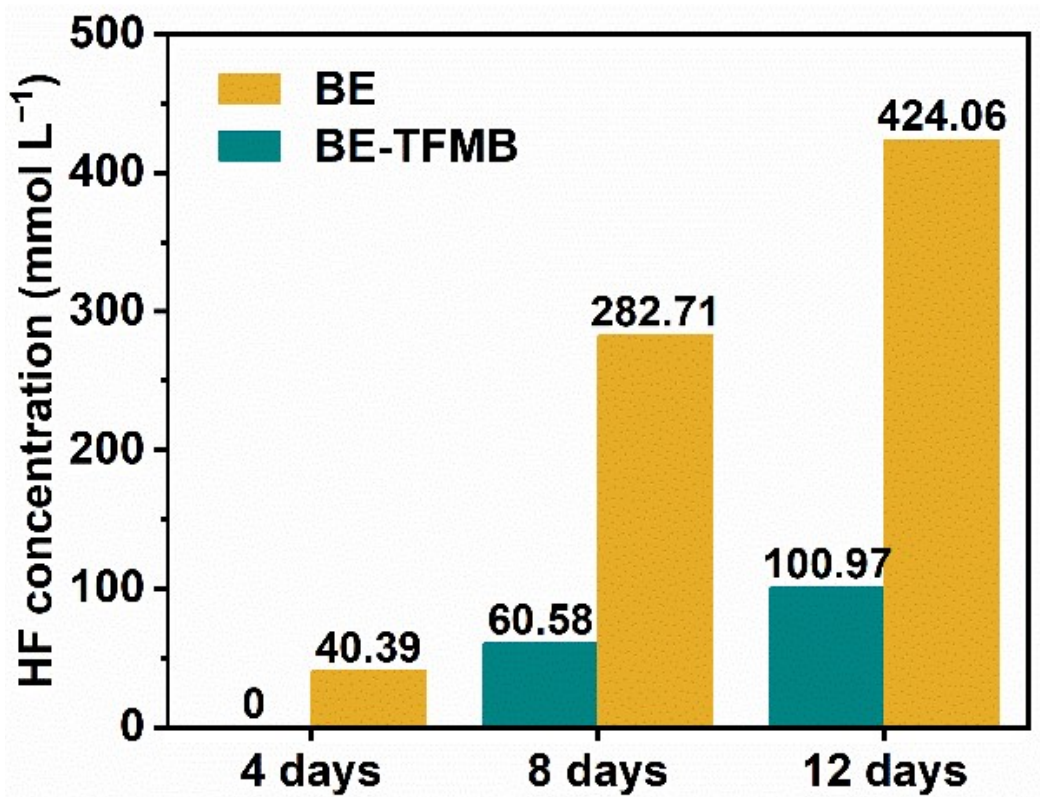

Figure S11. HF content in two electrolytes at different storage times (60 °C).

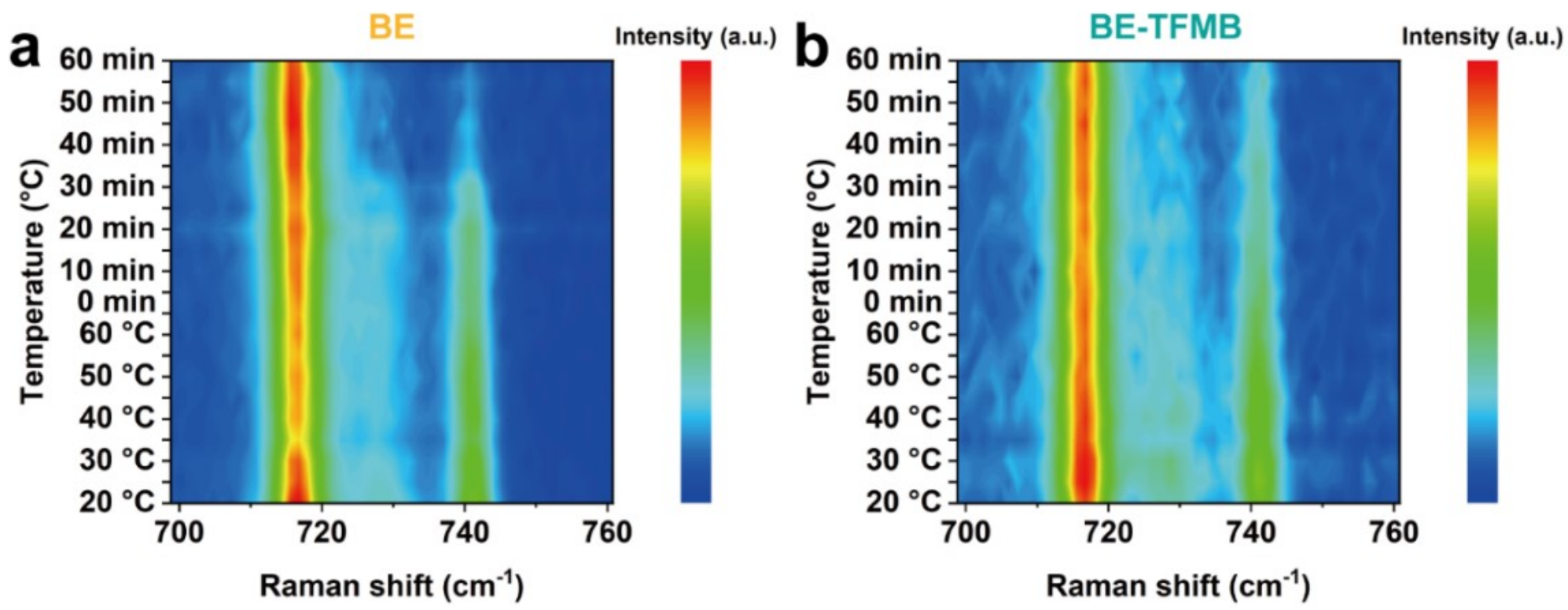

Figure S12. In-situ Raman spectra of two electrolytes at different temperature and storage times.

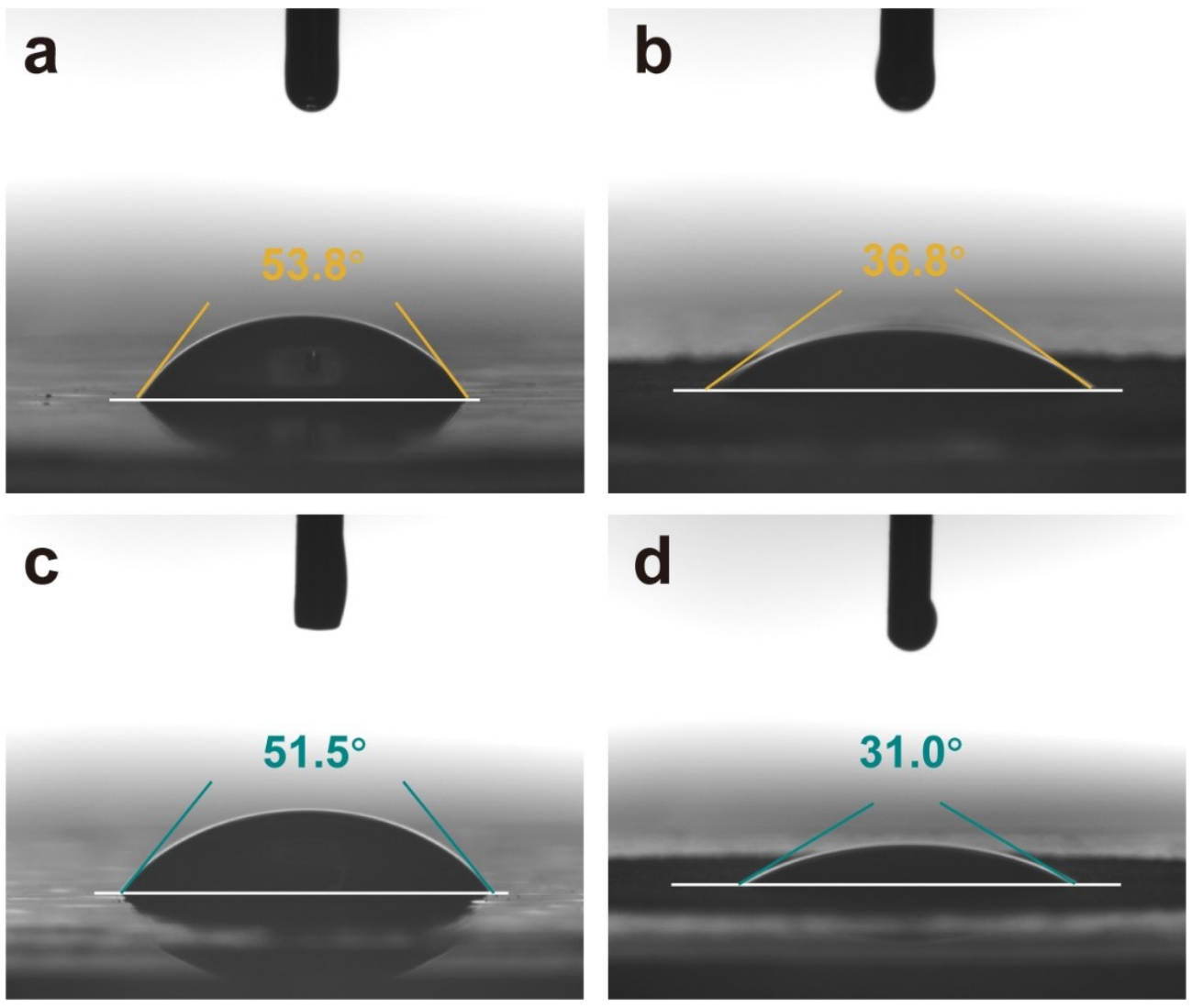

Figure S13. Contact angle of (a, b) BE and (c, d) BE-TFMB to the (a, c) Celgard 2325 and the (b, d) NCM811 cathode.

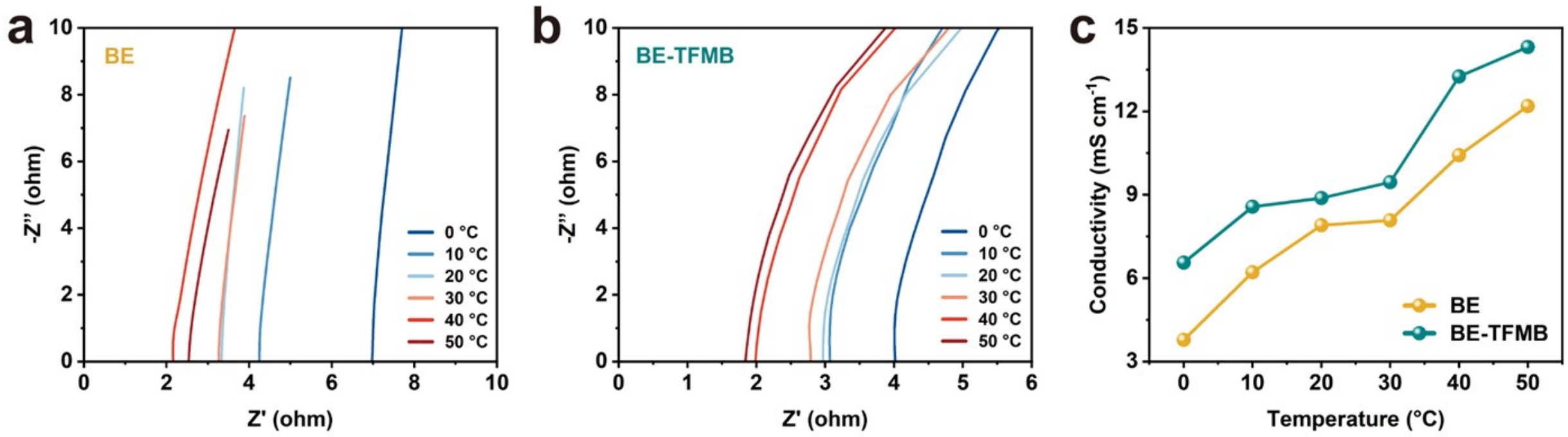

Figure S14. The EIS curves of SS||SS cells using (a) BE and (b) BE-TFMB and (c) the corresponding Li^+ conductivity of two electrolytes over a wide temperature range.

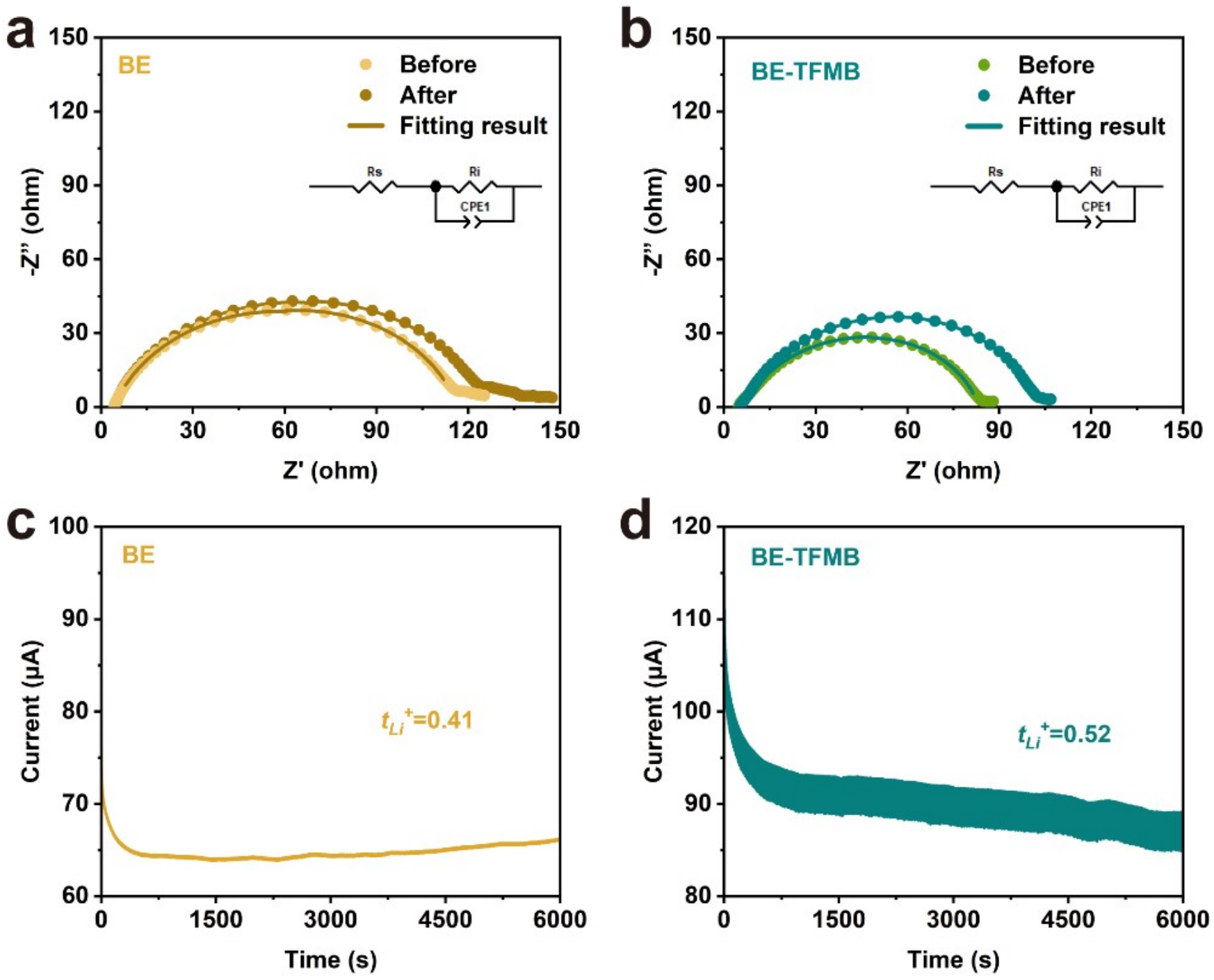

Figure S15. (a, b) EIS and (c, d) corresponding chronoamperometry curves of (a, c) BE and (b, d) BE-TFMB to calculate the Li^{+} transference numbers.

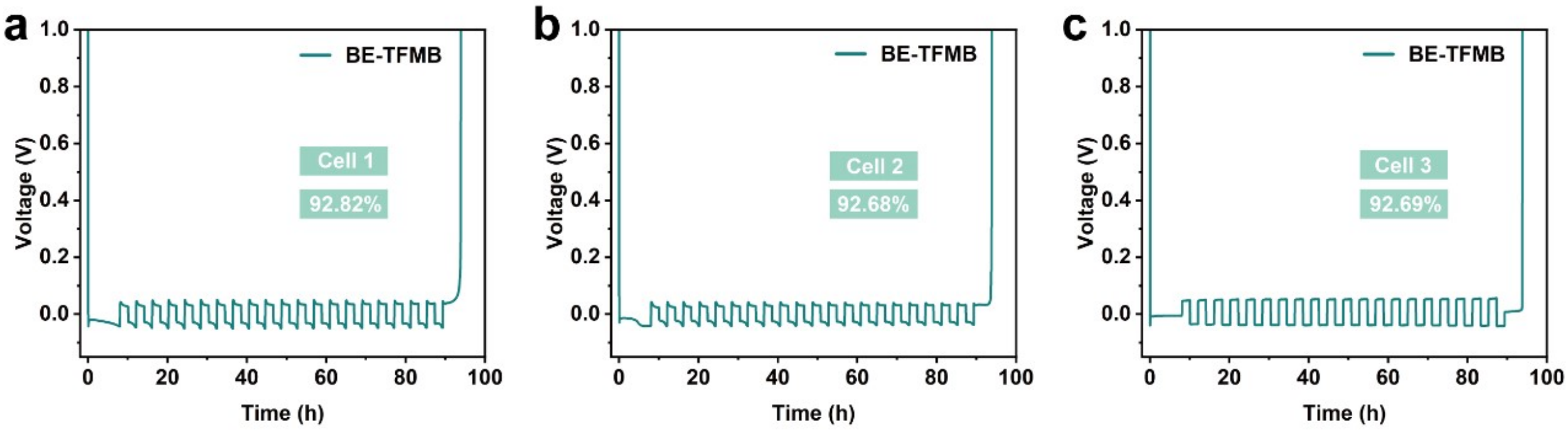

Figure S16. Li plating/stripping CEs of Li||Cu cells using BE-TFMB evaluated via Aurbach's measurement.

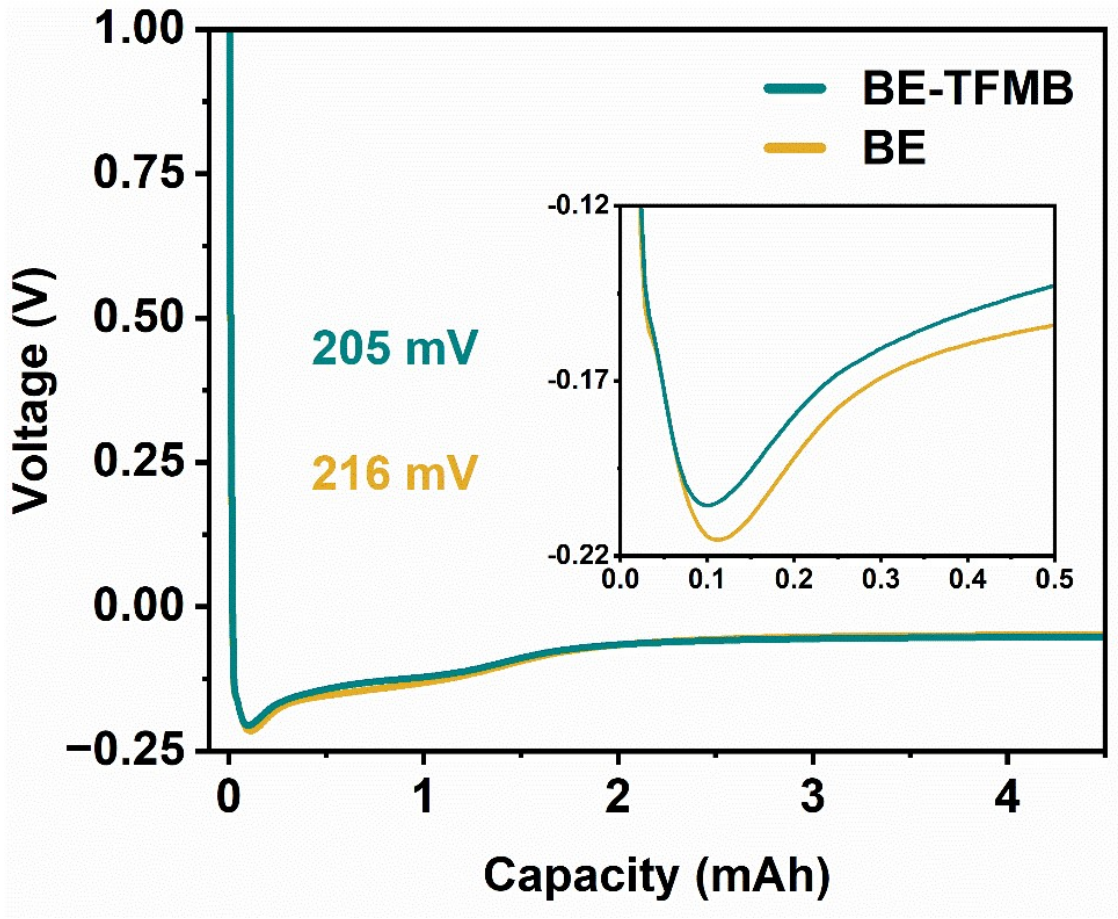

Figure S17. Voltage curves of Li deposition on Cu substrate in the two electrolytes.

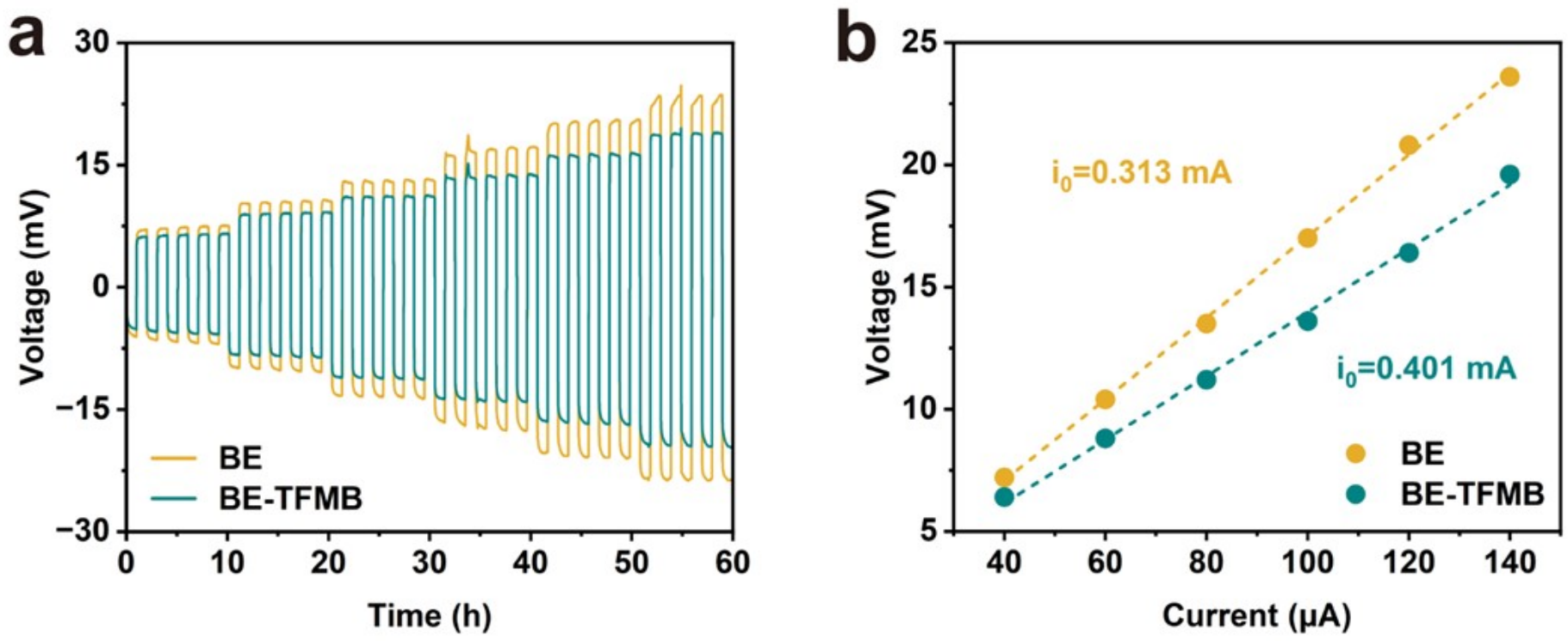

Figure S18. (a) Voltage-time profiles of Li||Li cells at various microcurrents from 40 to 140 μA with an interval of and 20 μA and (b) the corresponding i_0 for Li plating/stripping with two electrolytes.

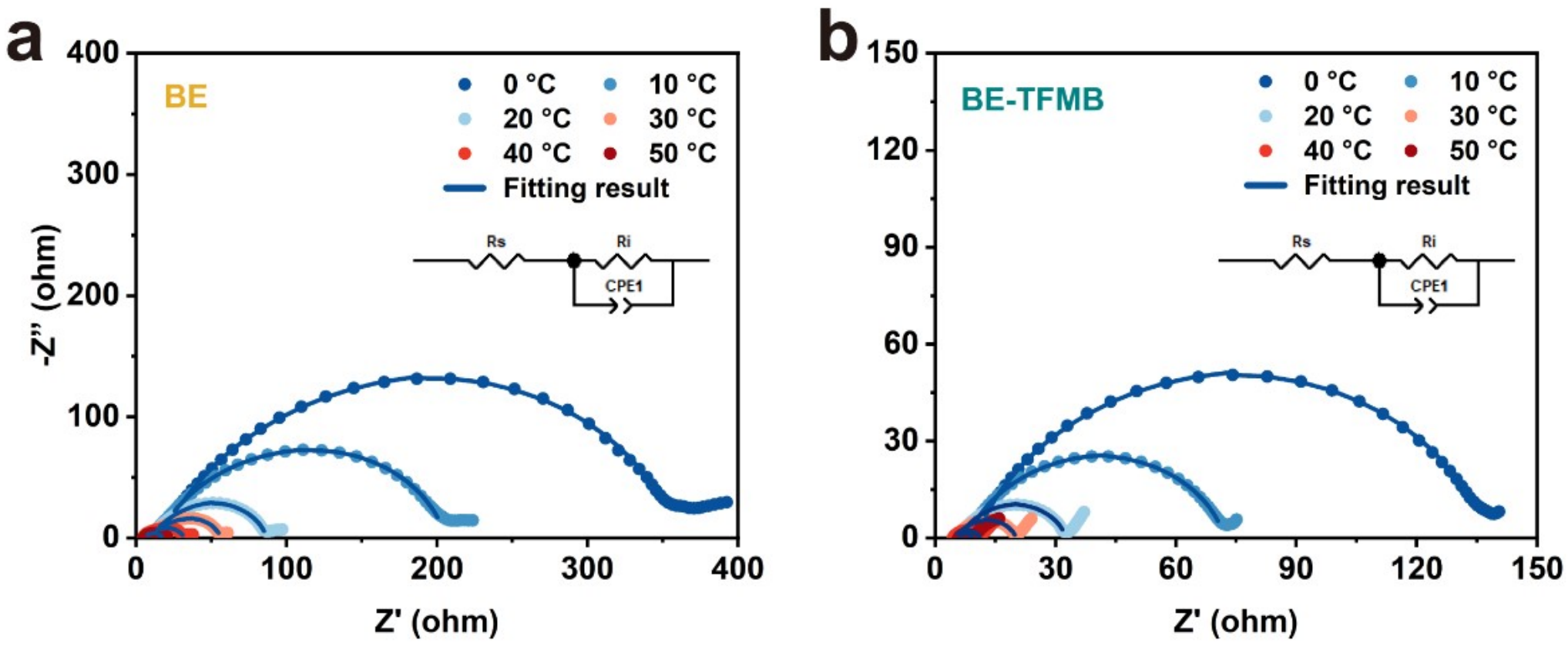

Figure S19. Temperature-dependent EIS curves of symmetric Li||Li cells using (a) BE and (b) BE-TFMB.

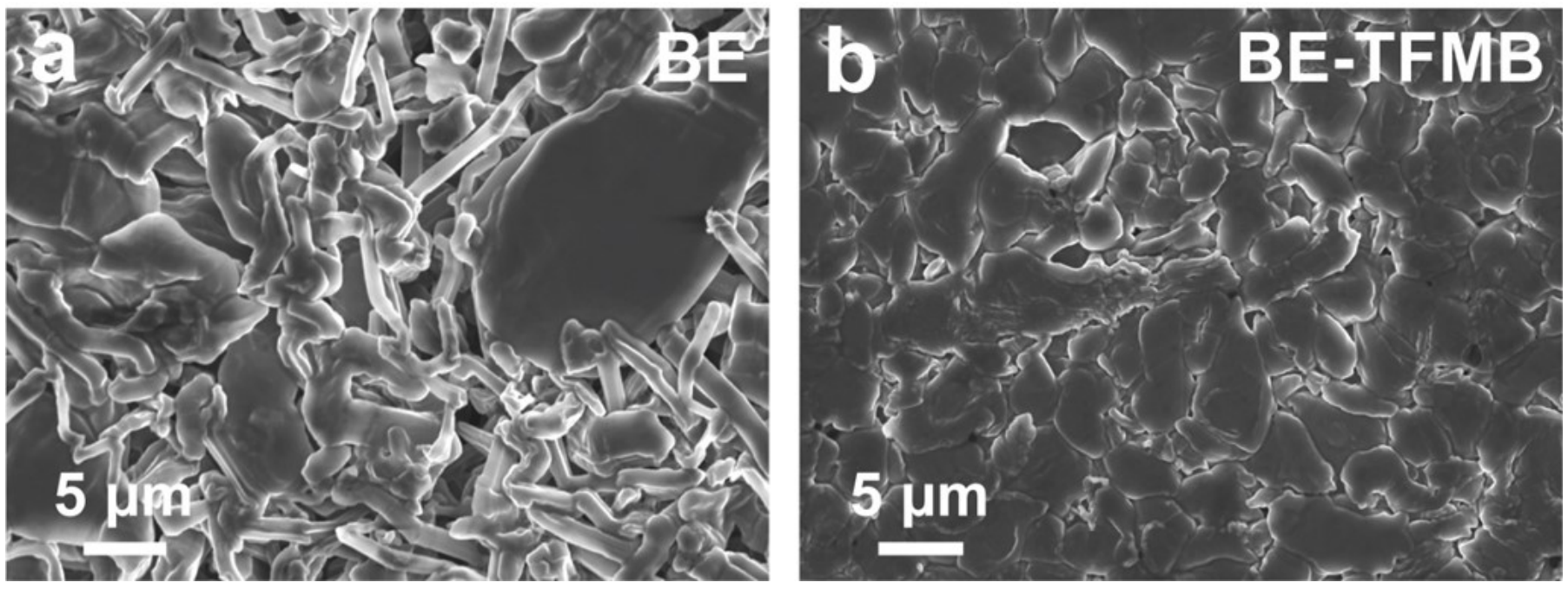

Figure S20. Top-view SEM images of cycled Li anode in Li||Li cells containing (a) BE and (b) BE-TFMB.

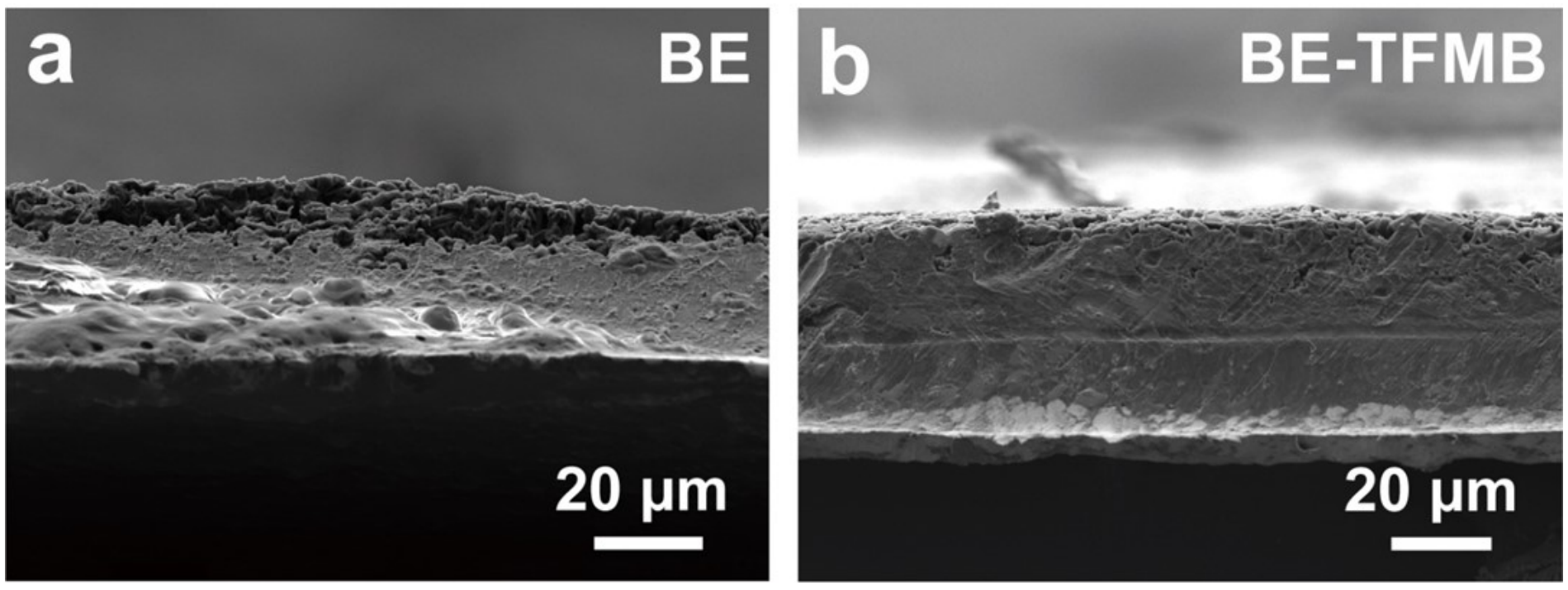

Figure S21. Cross-sectional view SEM images of cycled Li anode in Li||Li cells containing (a) BE and (b) BE-TFMB.

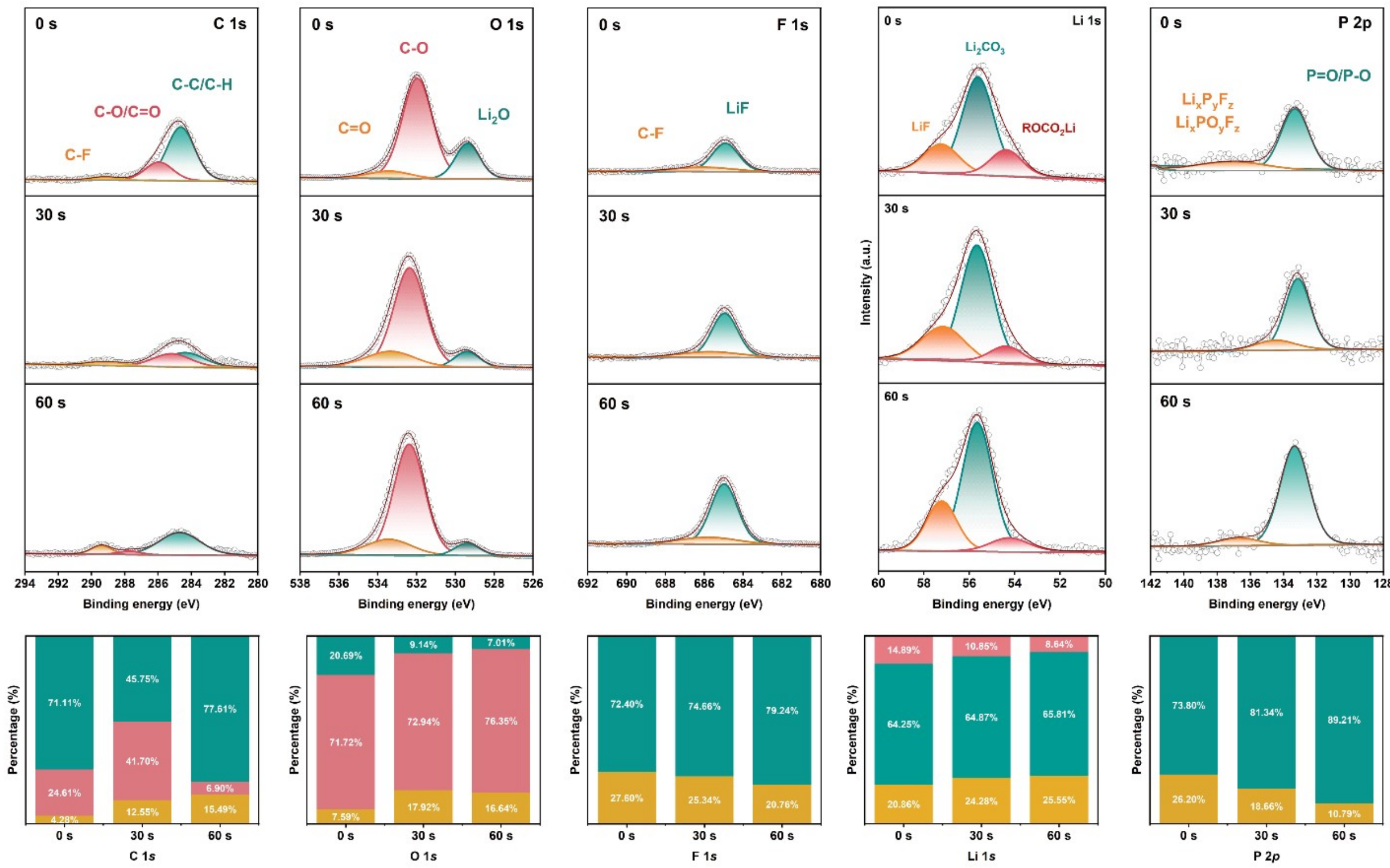

Figure S22. High-resolution XPS spectra of C 1*s*, O 1*s*, F 1*s*, Li 1*s*, P 2*p* and their corresponding component proportion (same color) for the SEI formed in BE.

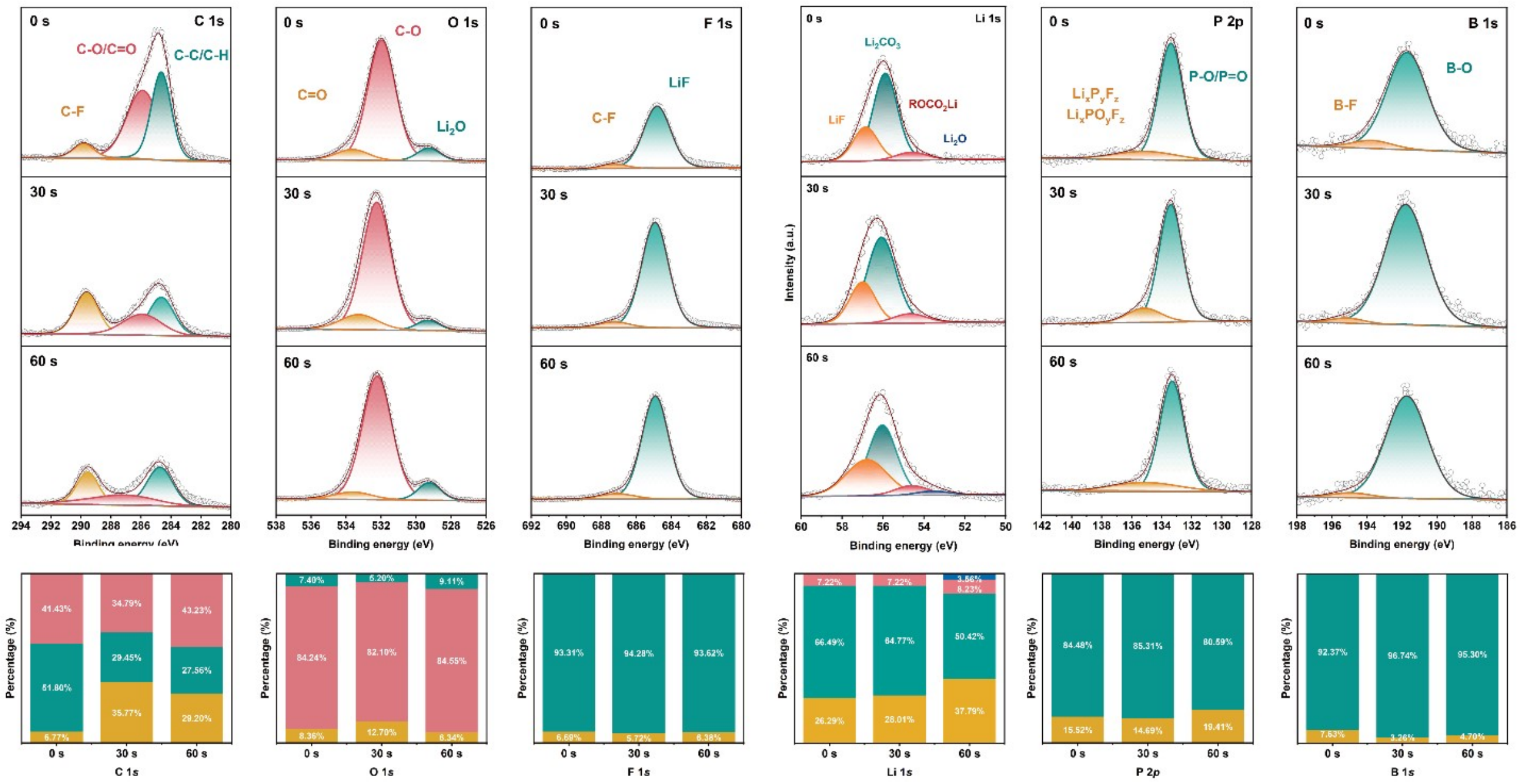

Figure S23. High-resolution XPS spectra of C 1*s*, O 1*s*, F 1*s*, Li 1*s*, P 2*p*, B 1*s* and their corresponding component proportion (same color) for the SEI formed in BE-TFMB.

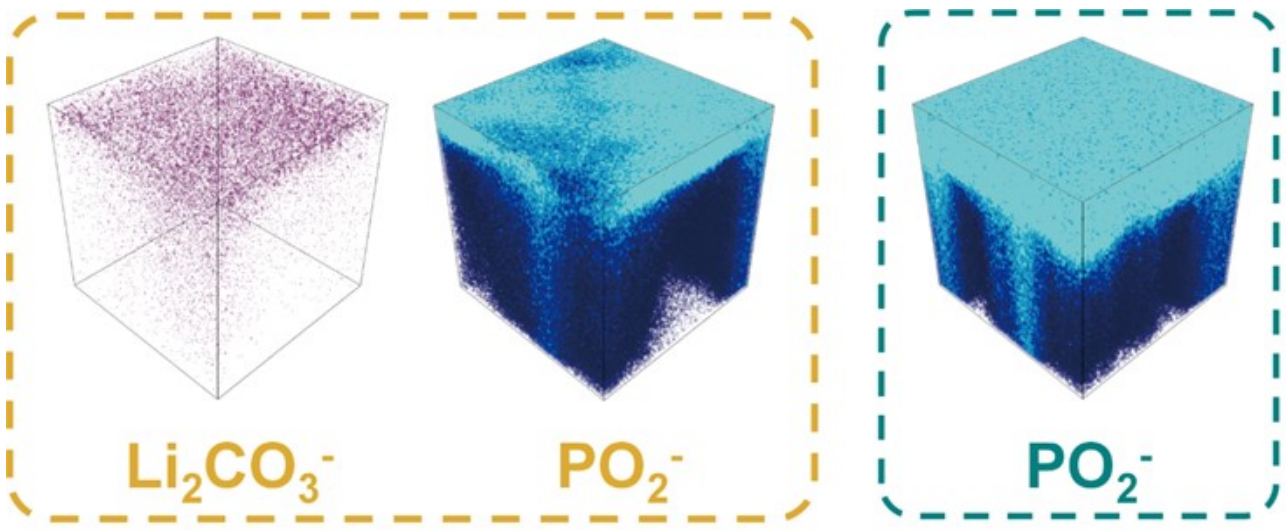

Figure S24. 3D TOF-SIMS mappings of the $Li_2CO_3^-$ and PO_2^- species of the SEI in BE and PO_2^- species of the SEI in BE-TFMB.

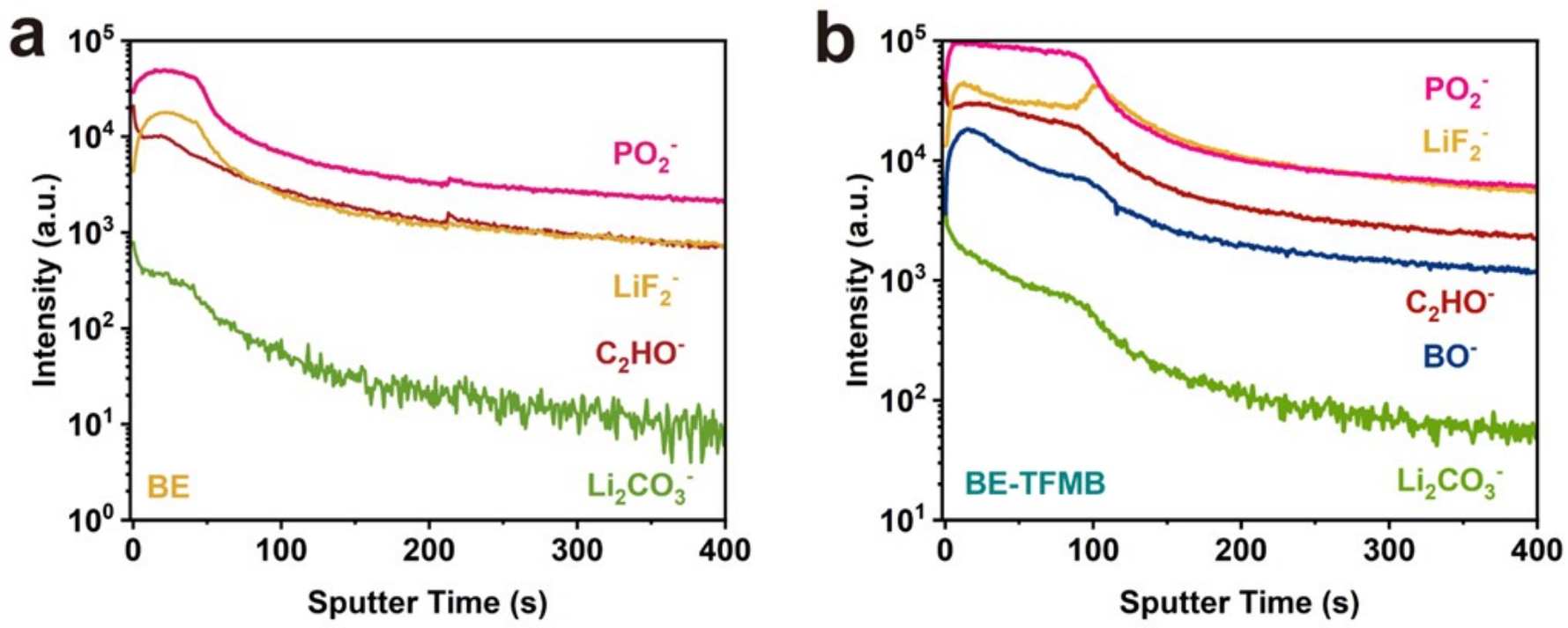

Figure S25. The corresponding intensity sputter profiles of SEI formed in (a) BE and (b) BE-TFMB.

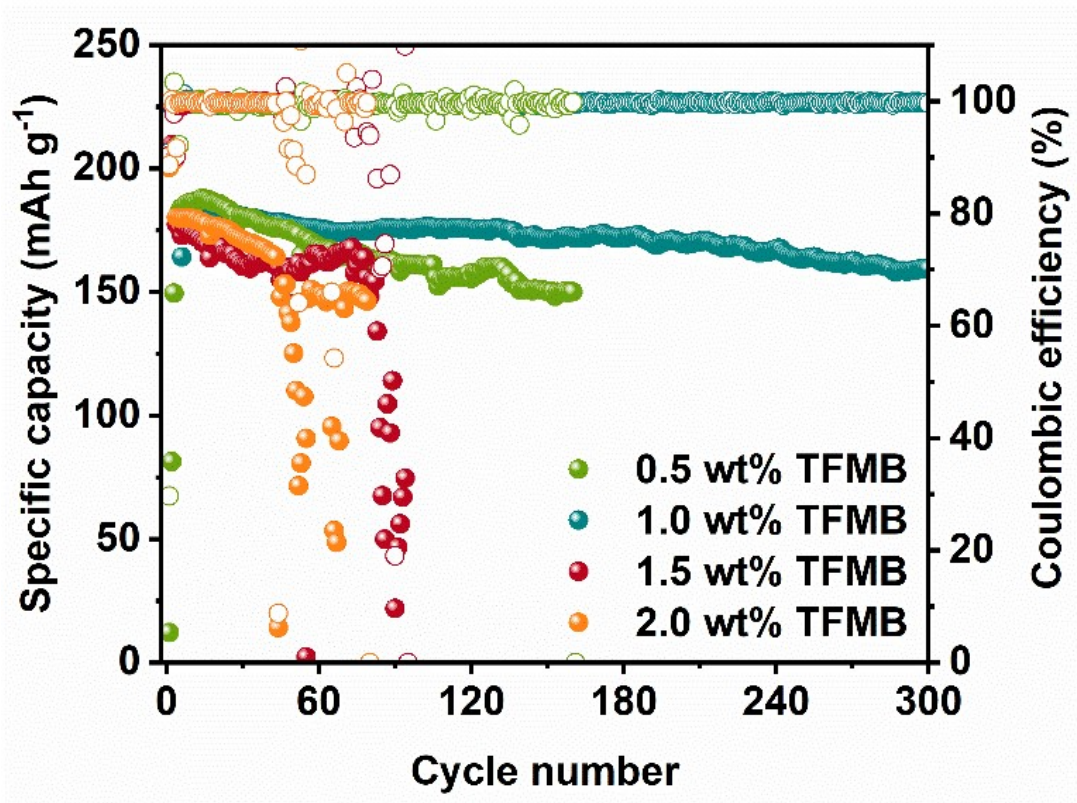

Figure S26. Cycling performance of Li||NCM811 cells using LB008 with different TFMB contents at 30 °C with a voltage range of 3-4.3 V.

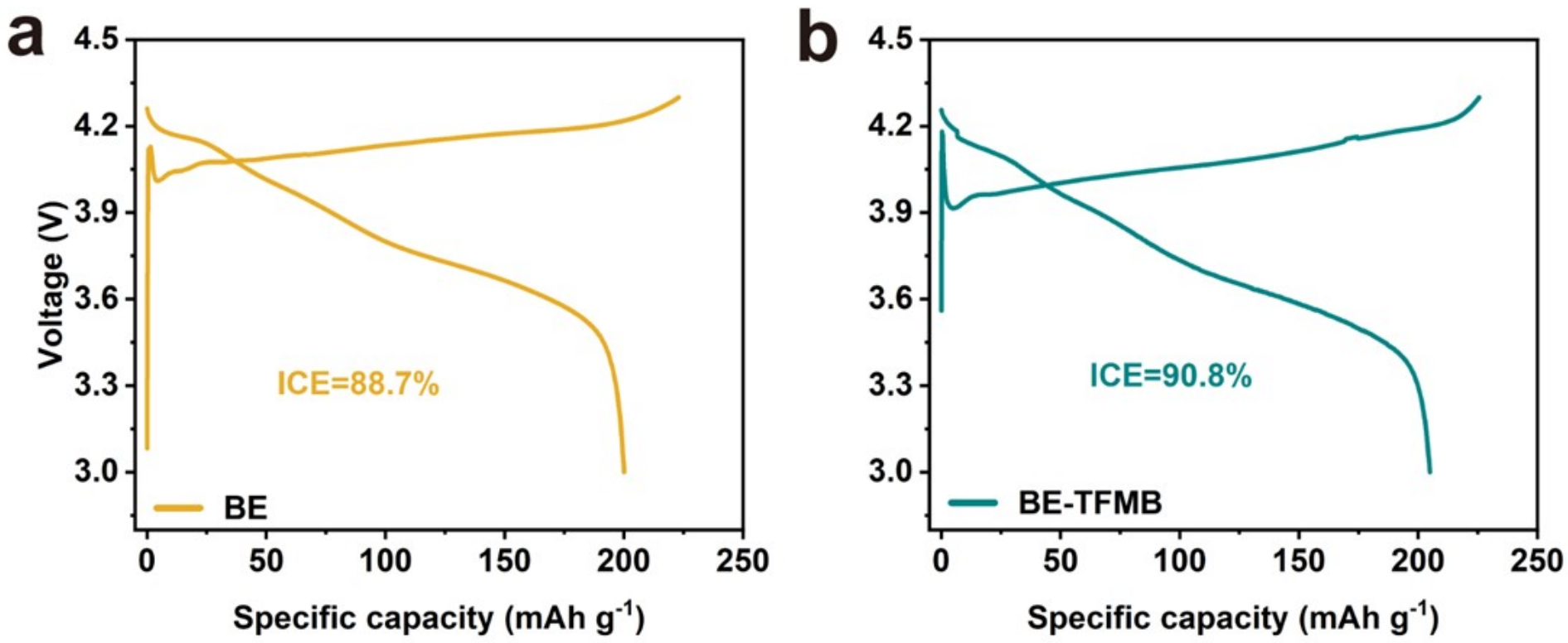

Figure S27. The charge/discharge curves and CEs of Li||NCM811 cells at the first cycle.

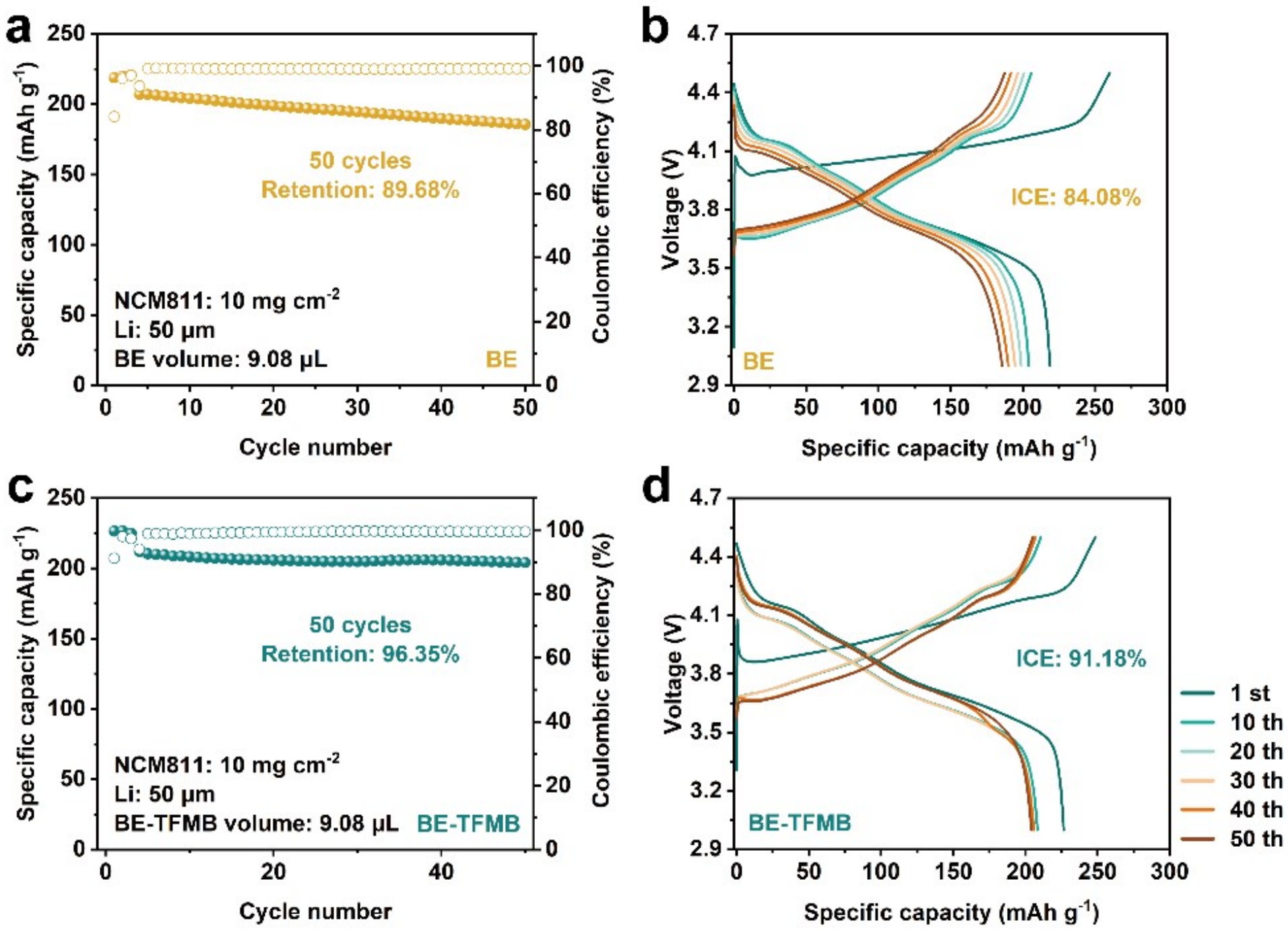

Figure S28. (a, c) Cycling performance and (b, d) corresponding charge/discharge curves of Li||NCM811 cells using (a, b) BE and (c, d) BE-TFMB at 0.5 C.

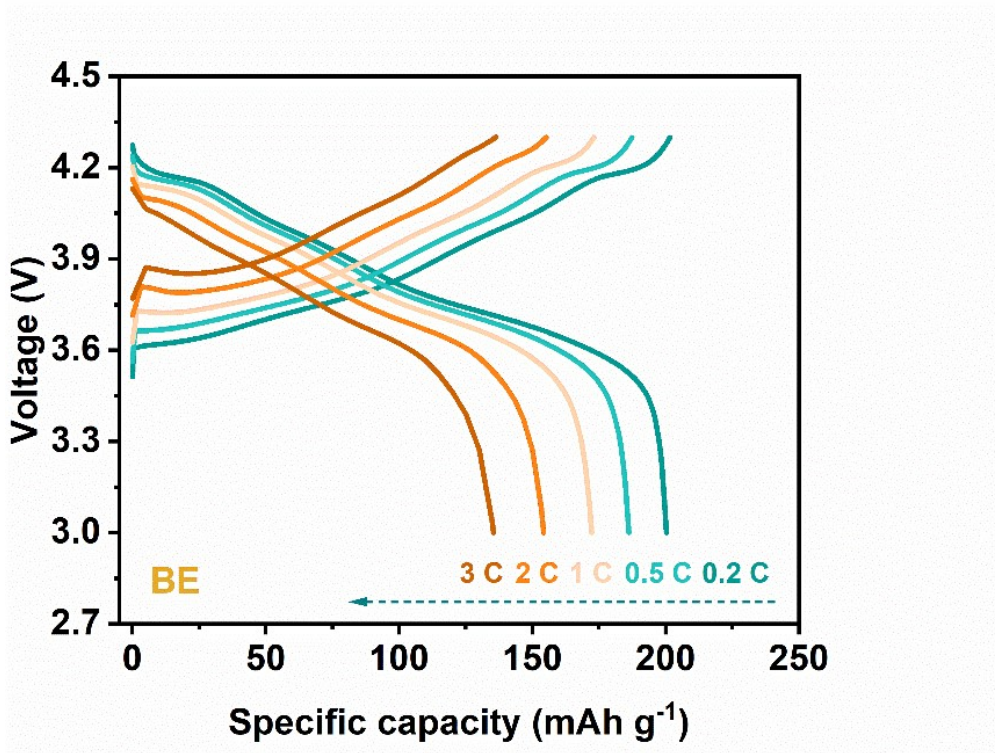

Figure S29. Charge/discharge curves of Li||NCM811 cells using BE at different current densities.

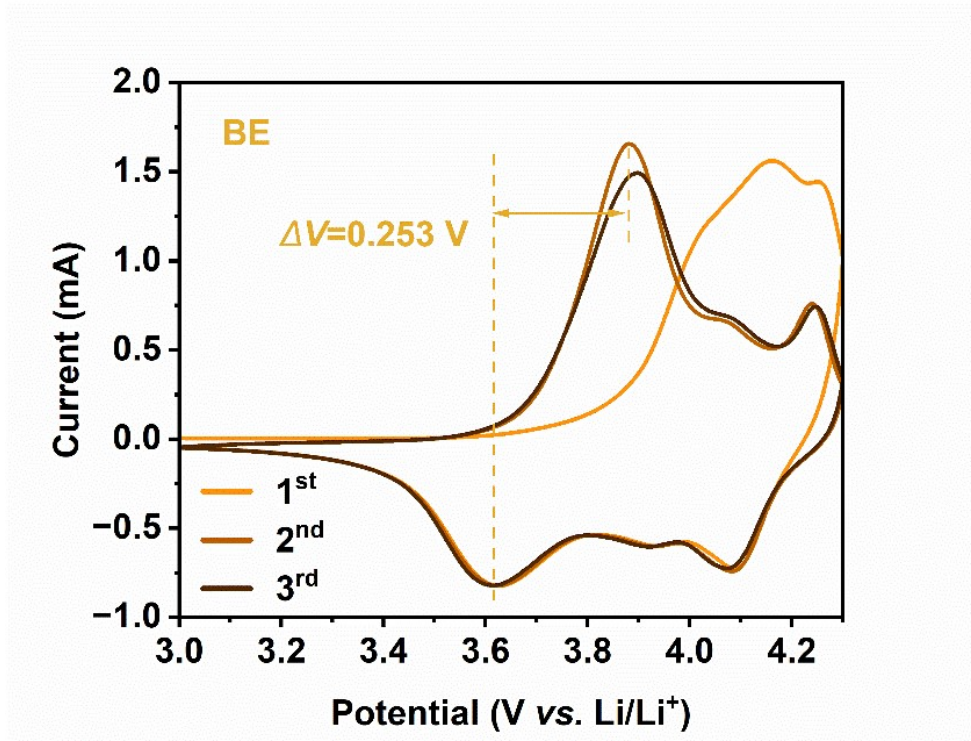

Figure S30. CV curves of Li||NCM811 cells using BE.

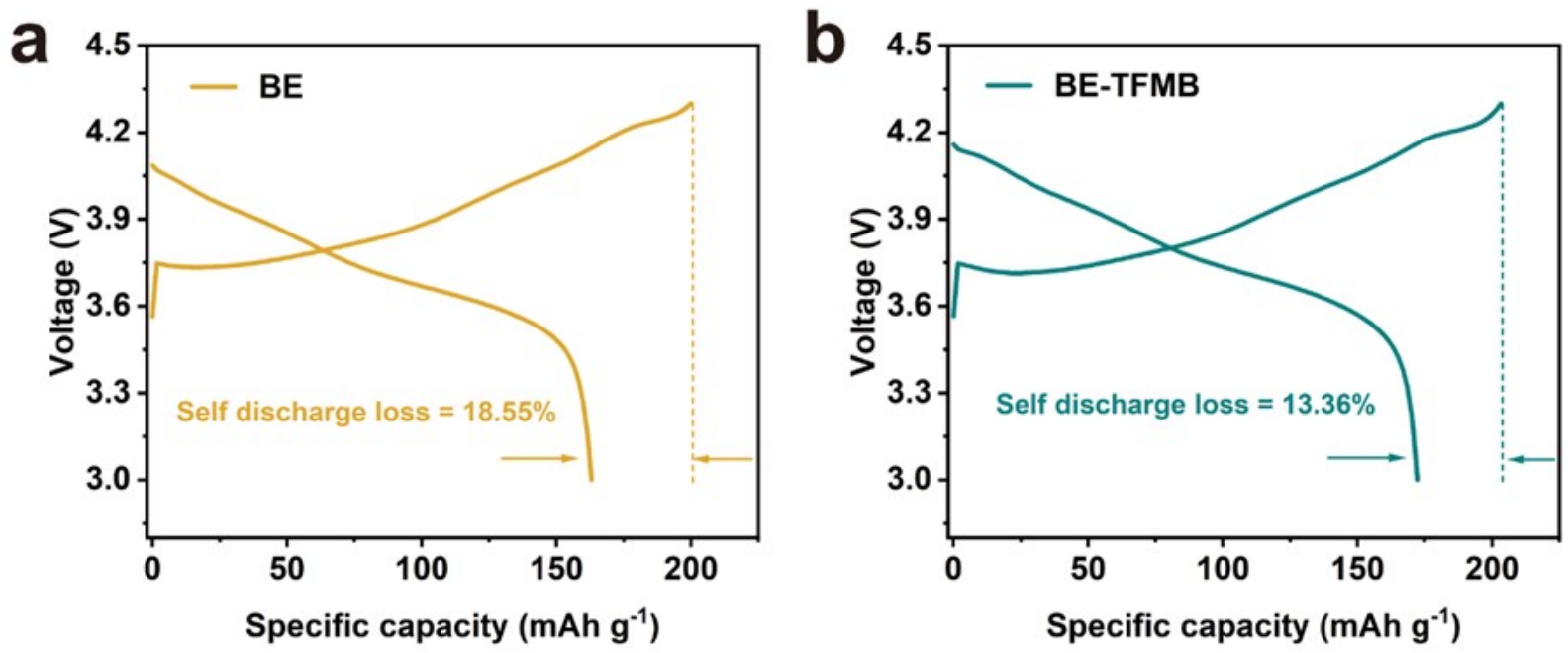

Figure S31. Self-discharge tests and the corresponding voltage-capacity profiles of Li||NCM811 cells using (a) BE and (b) BE-TFMB.

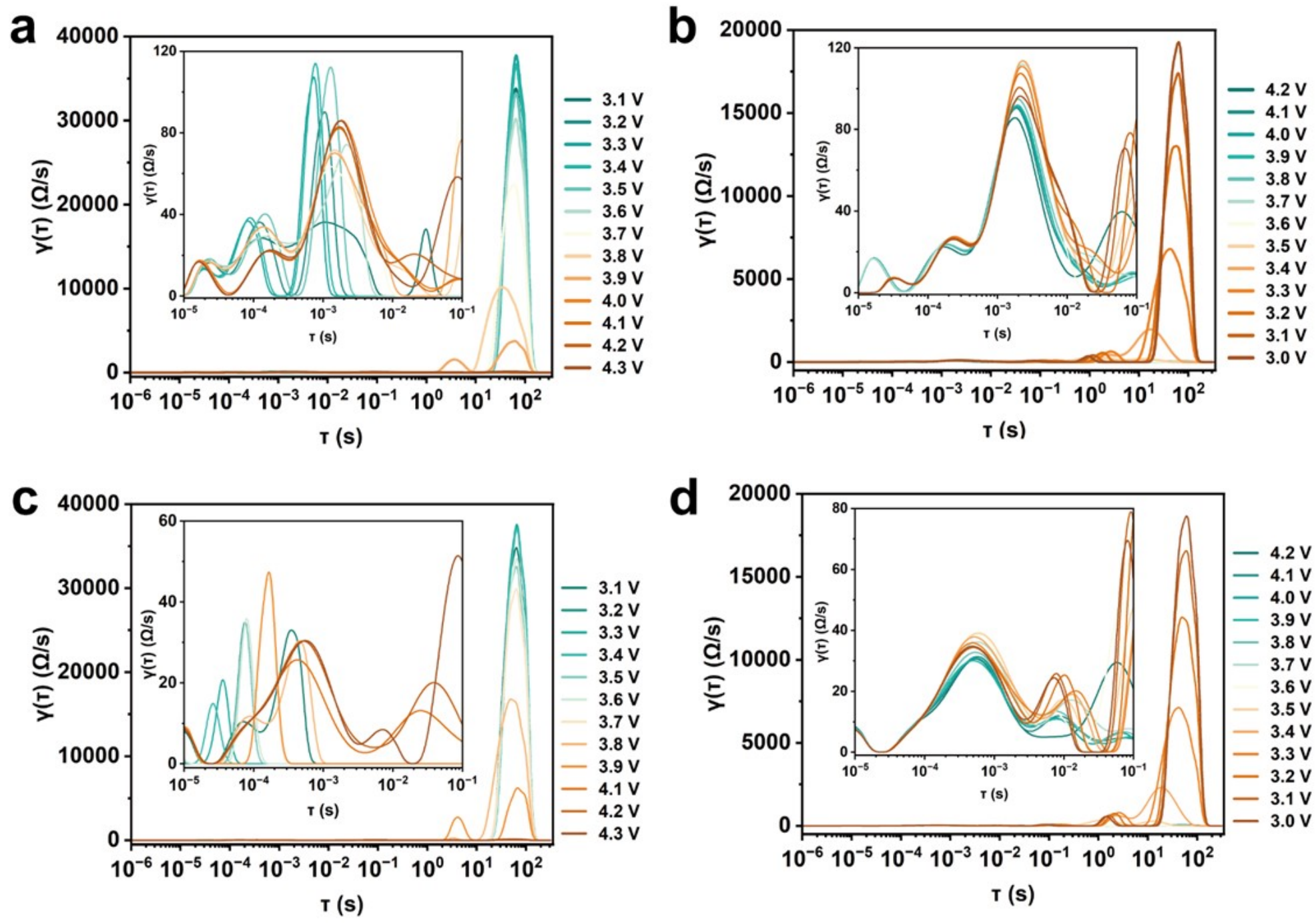

Figure S32. In-situ DRT data of Li||NCM811 cells with (a, b) BE and (c, d) BE-TFMB in (a, c) charge status and (b, d) discharge status.

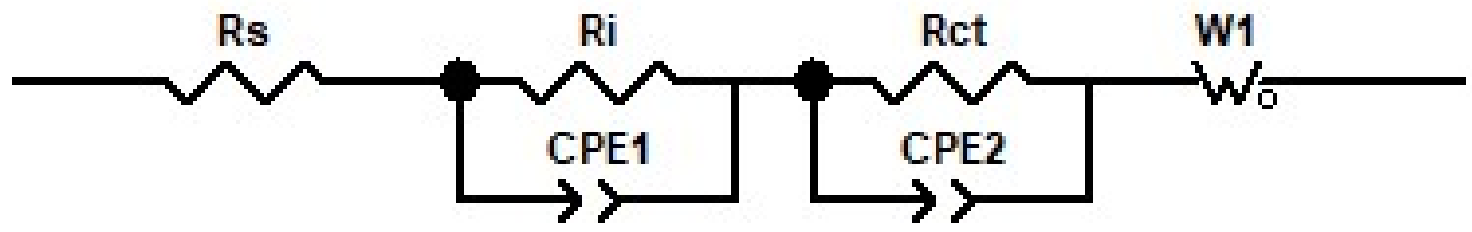

Figure S33. The corresponding equivalent circuit for EIS fitting of Li||NCM811 cells.

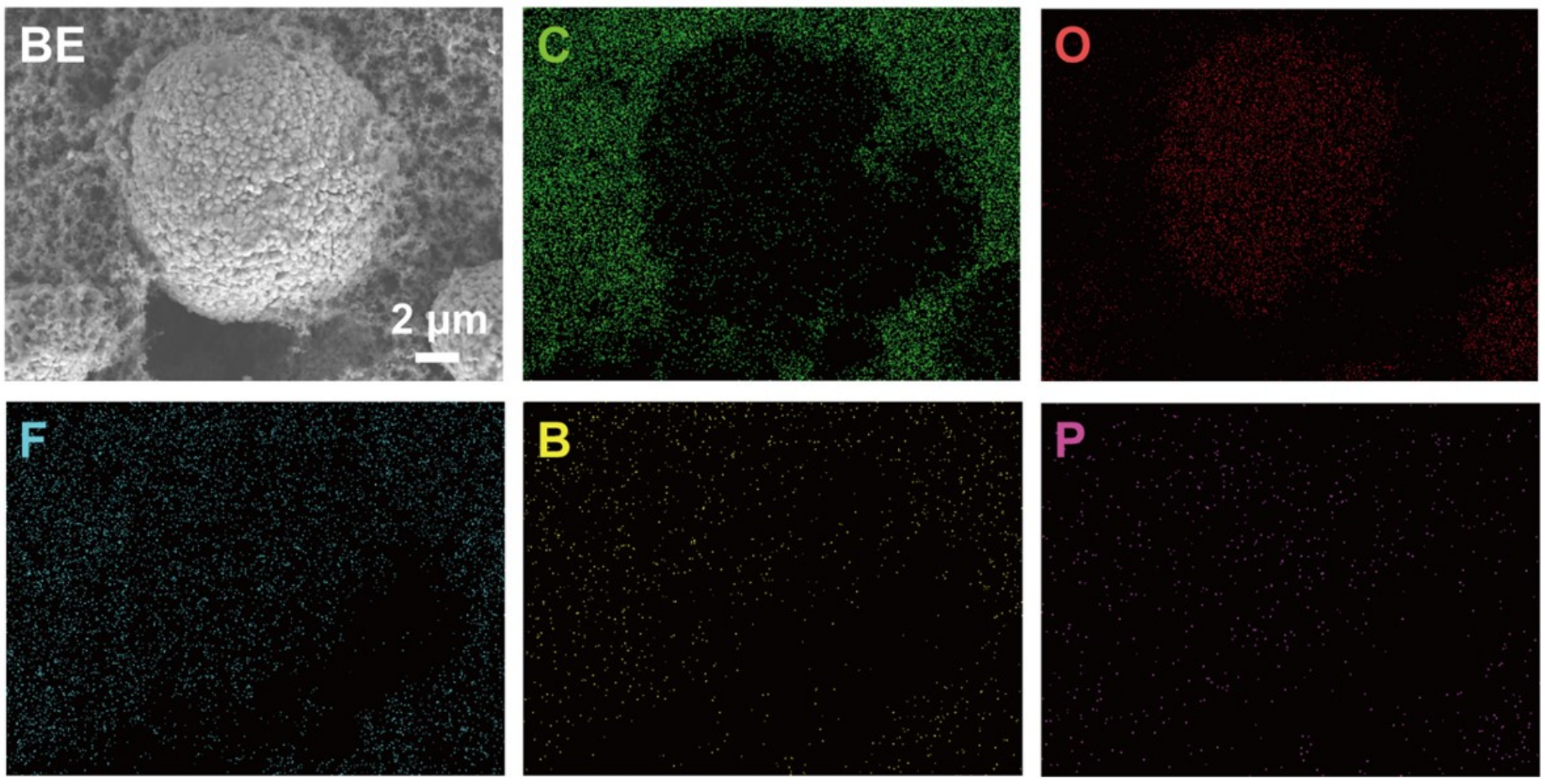

Figure S34. SEM images and corresponding EDS mapping of the cycled NCM811 cathode in BE.

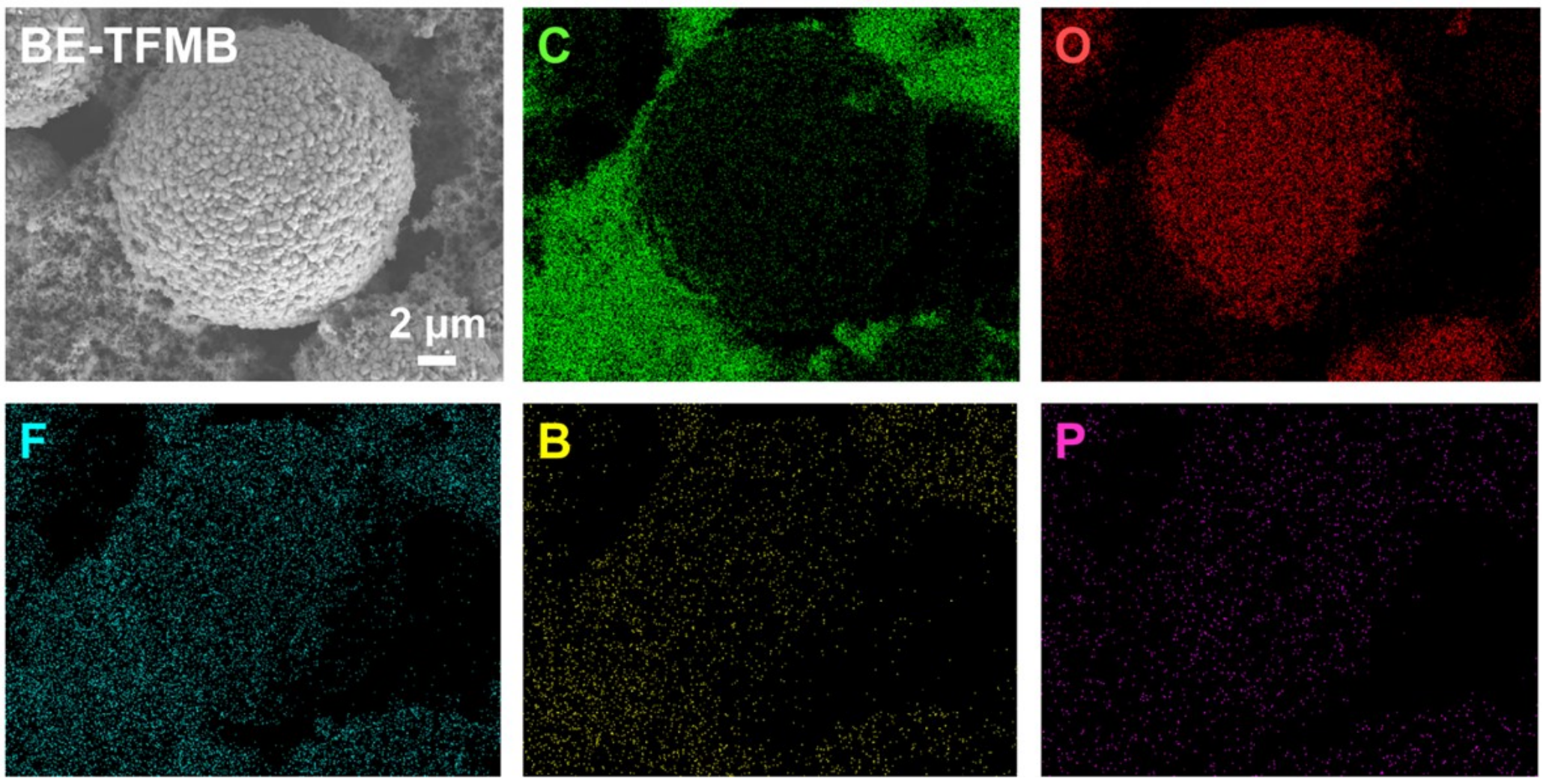

Figure S35. SEM images and corresponding EDS mapping of the cycled NCM811 cathode in BE-TFMB.

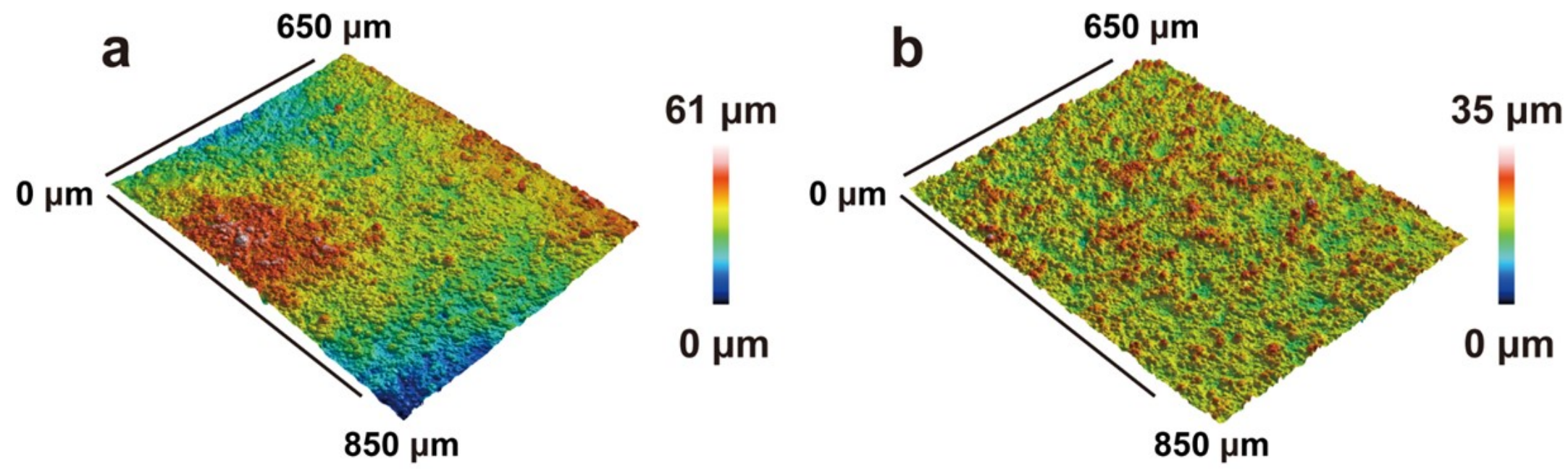

Figure S36. CLSM optical images of the cycled NCM811 cathode in (a) BE and (b) BE-TFMB (50X).

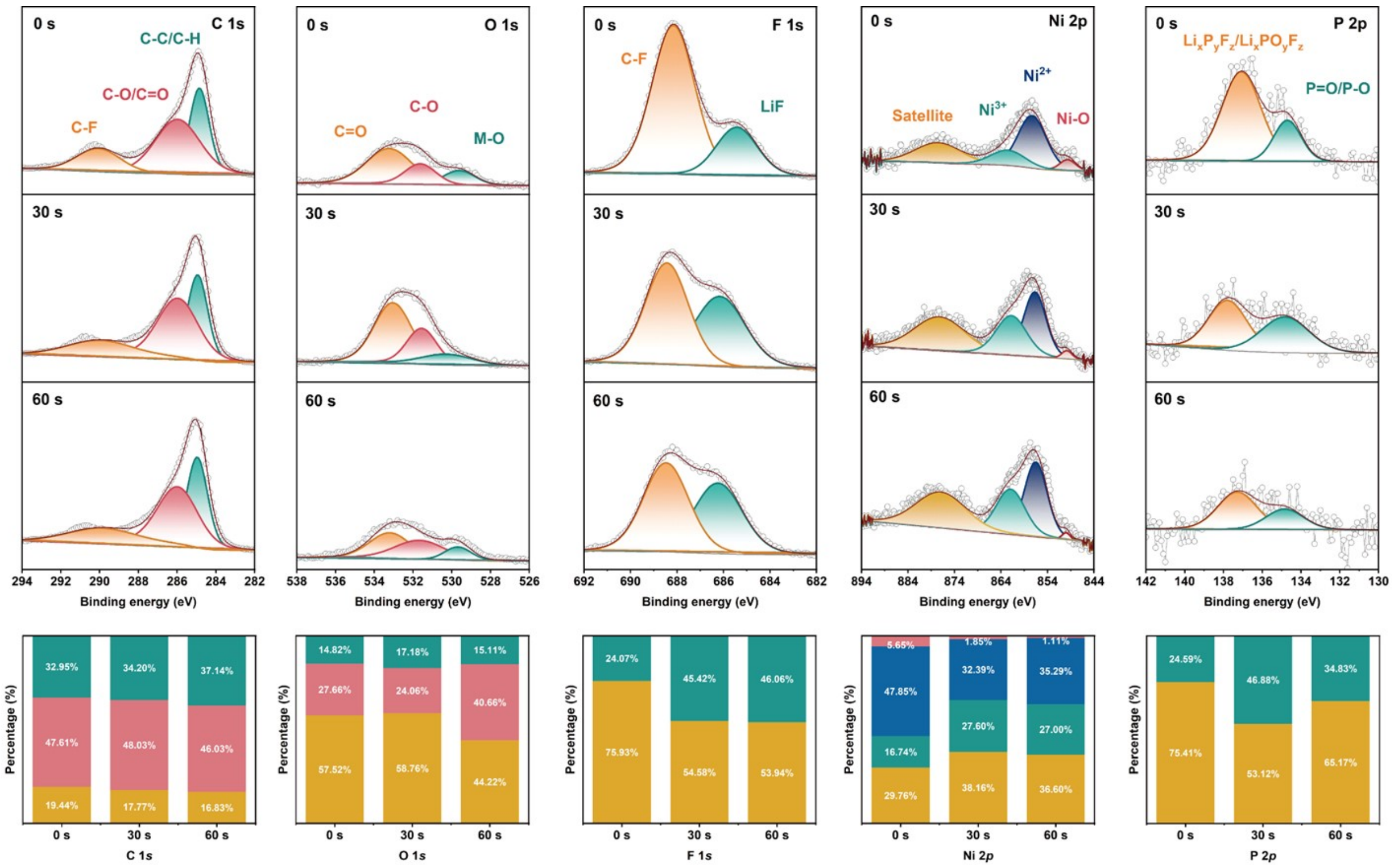

Figure S37. High-resolution XPS spectra of C 1*s*, O 1*s*, F 1*s*, Li 1*s*, P 2*p* and their corresponding component proportion (same color) for the CEI formed in BE.

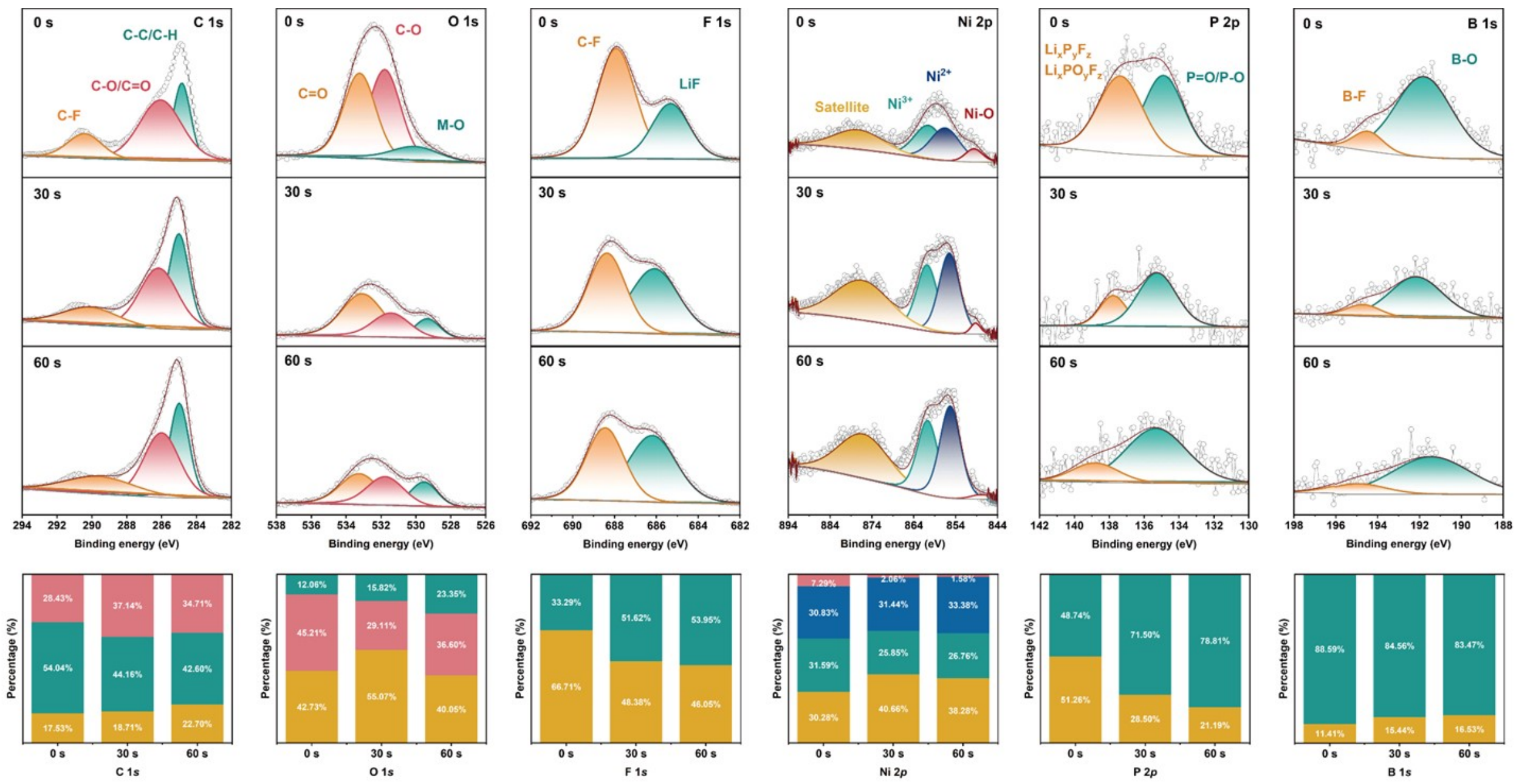

Figure S38. High-resolution XPS spectra of C 1*s*, O 1*s*, F 1*s*, Li 1*s*, P 2*p*, B 1*s* and their corresponding component proportion (same color) for the CEI formed in BE-TFMB.

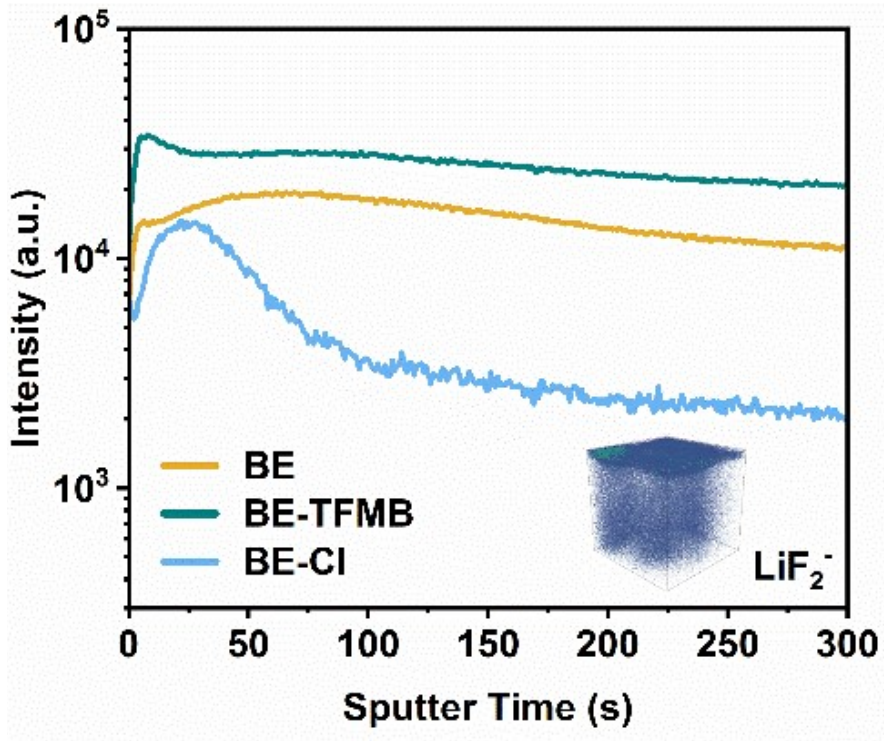

Figure S39. 3D TOF-SIMS mappings and the corresponding intensity sputter profiles of the LiF_2^- species of the SEI in BE-Cl.

To ensure a single source of LiF_2^-, we will seek an alternative to $LiPF_6$ in BE-TFMB. Common Li salts such as LiTFSI and LiFSI contain F and thus cannot serve as a non-F reference. LiBOB is limited by its inferior solubility in carbonate-based solvents and low ionic conductivity, while $LiNO_3$ also exhibits poor solubility in carbonate-based solvents and is commonly used in ether-based electrolytes. Furthermore, LiCl is soluble only in aqueous solutions and its poor dissolution in organic systems further limits its potential as a control Li salt. Therefore, considering that $LiClO_4$ is F-free, has good solubility, and exhibits high ionic conductivity, we selected it as an alternative Li salt

As shown in **Figure S39**, the TOF-SIMS images show that the LiF_2^- signal from the SEI in BE-Cl tends to stabilize after an etching time exceeding 100 s. This result confirms that the $–CF_3$ groups in the additive can indeed participate in the formation of the interphases, which corresponds to their high electronegativity and low LUMO energies.

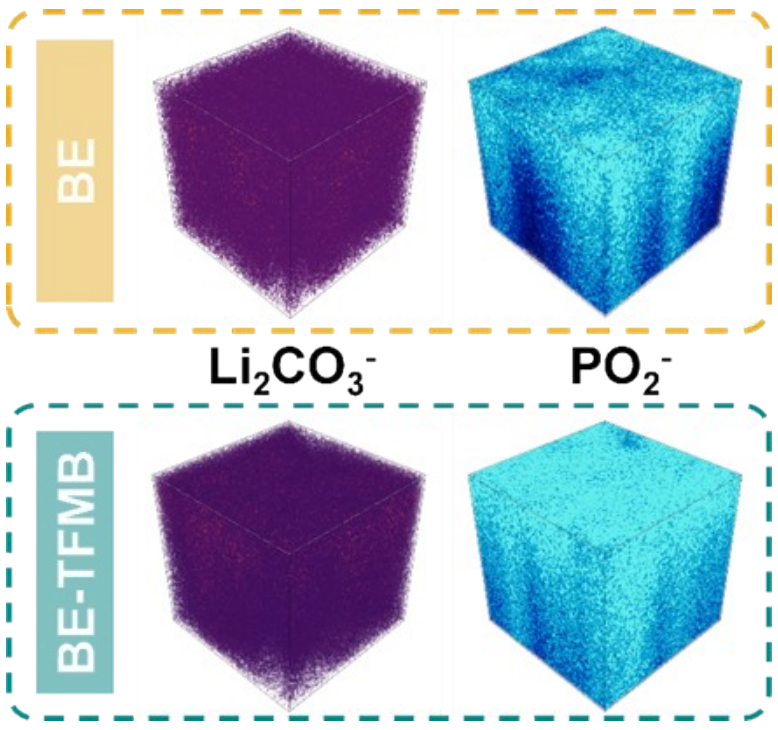

Figure S40. 3D TOF-SIMS mappings of the $Li_2CO_3^-$ and PO_2^- species of the CEI in BE and BE-TFMB.

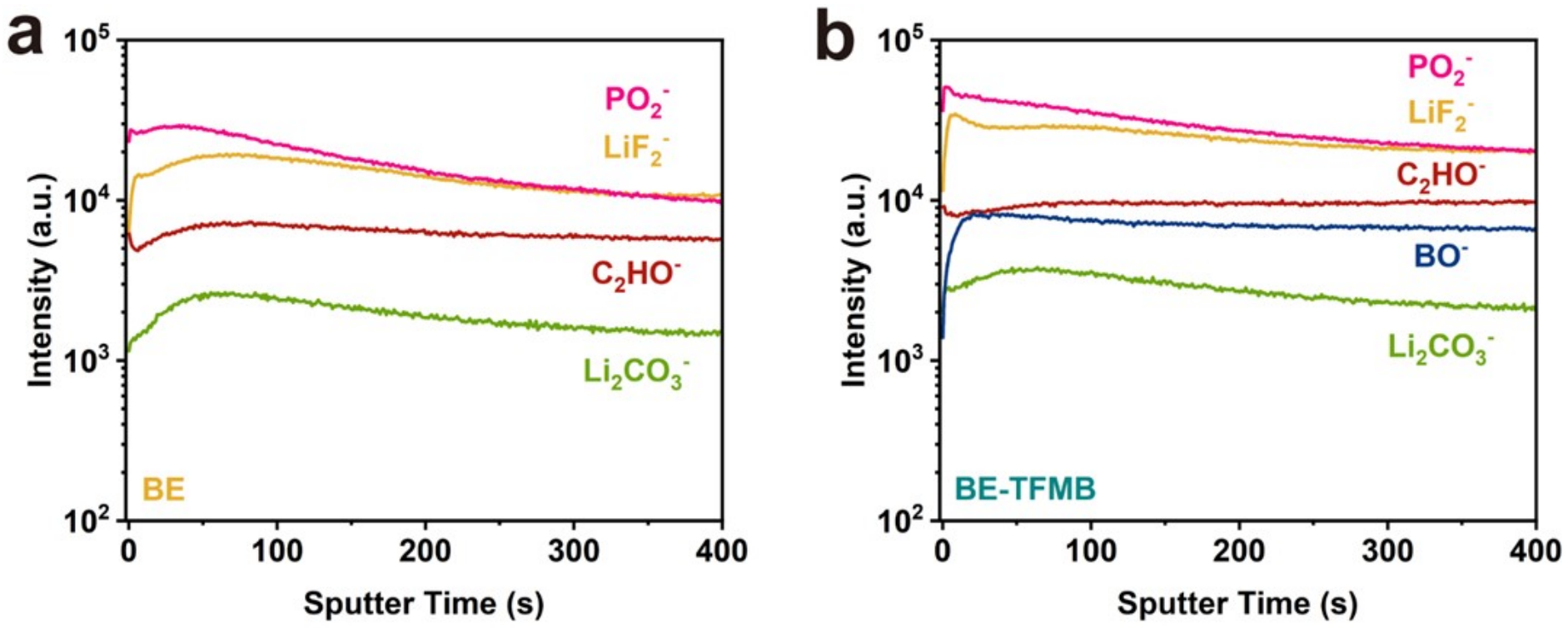

Figure S41. The corresponding intensity sputter profiles of CEI formed in (a) BE and (b) BE-TFMB.

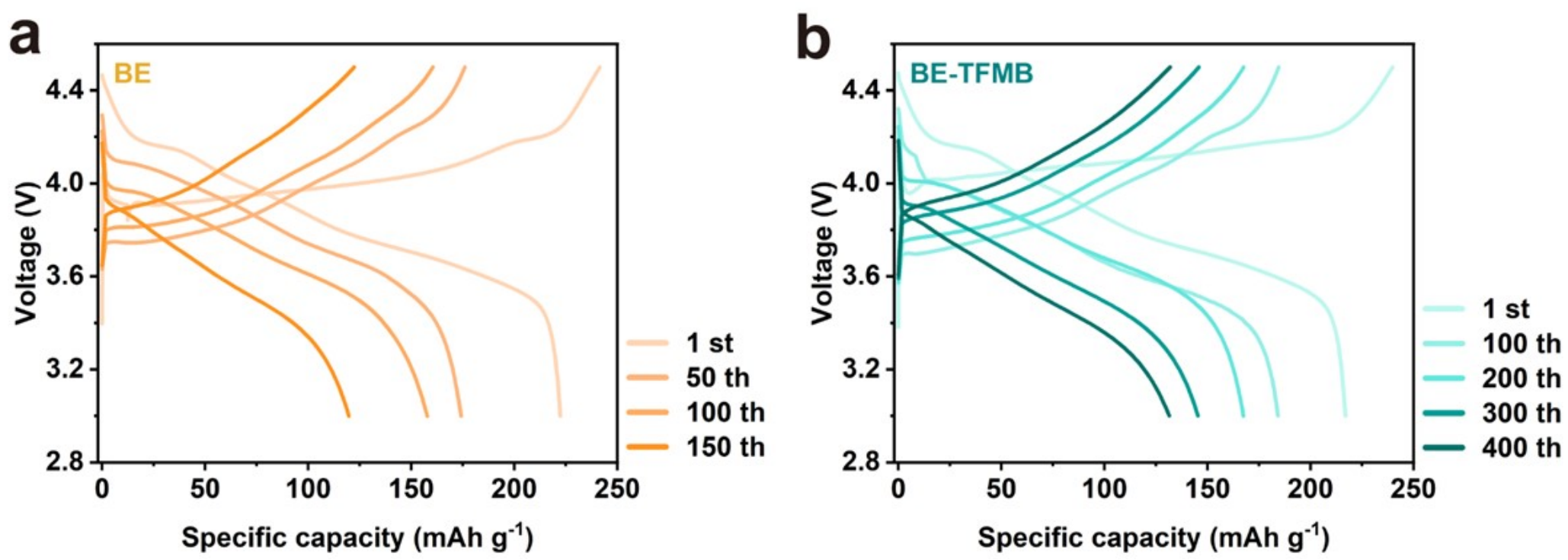

Figure S42. Charge/discharge curves of Li||NCM811 cells using (a) BE and (b) BE-TFMB at a high cutoff voltage of 4.5 V.

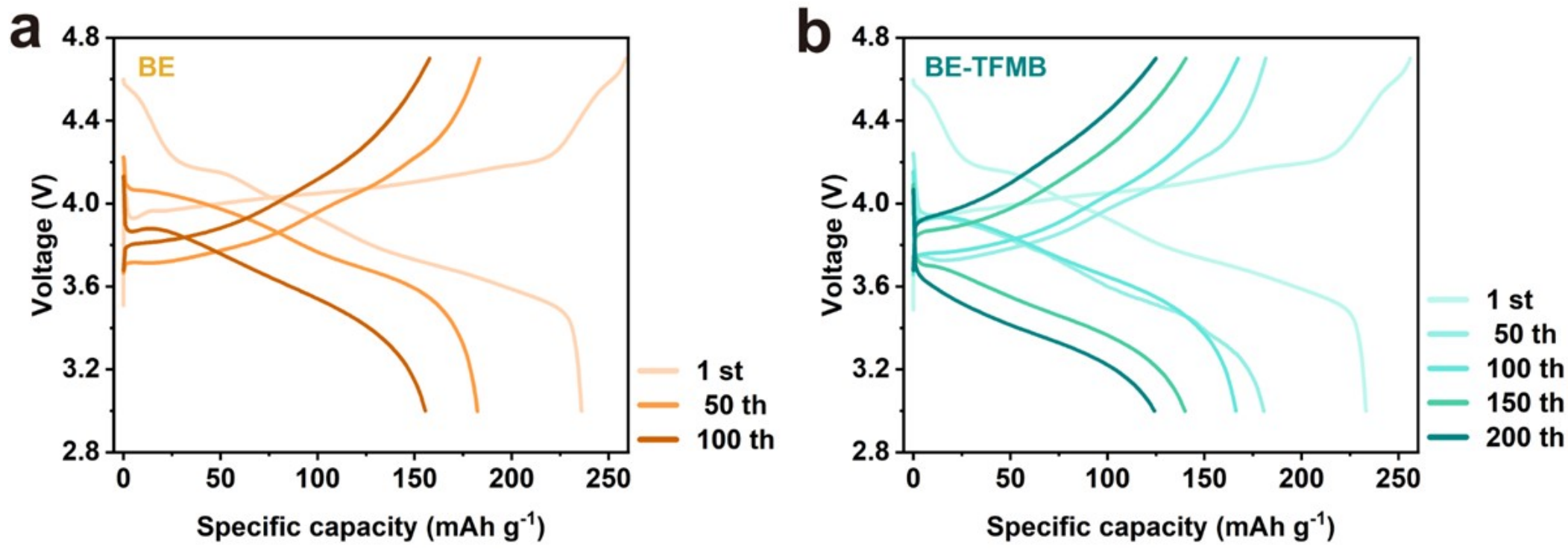

Figure S43. Charge/discharge curves of Li||NCM811 cells using (a) BE and (b) BE-TFMB at a high cutoff voltage of 4.7 V.

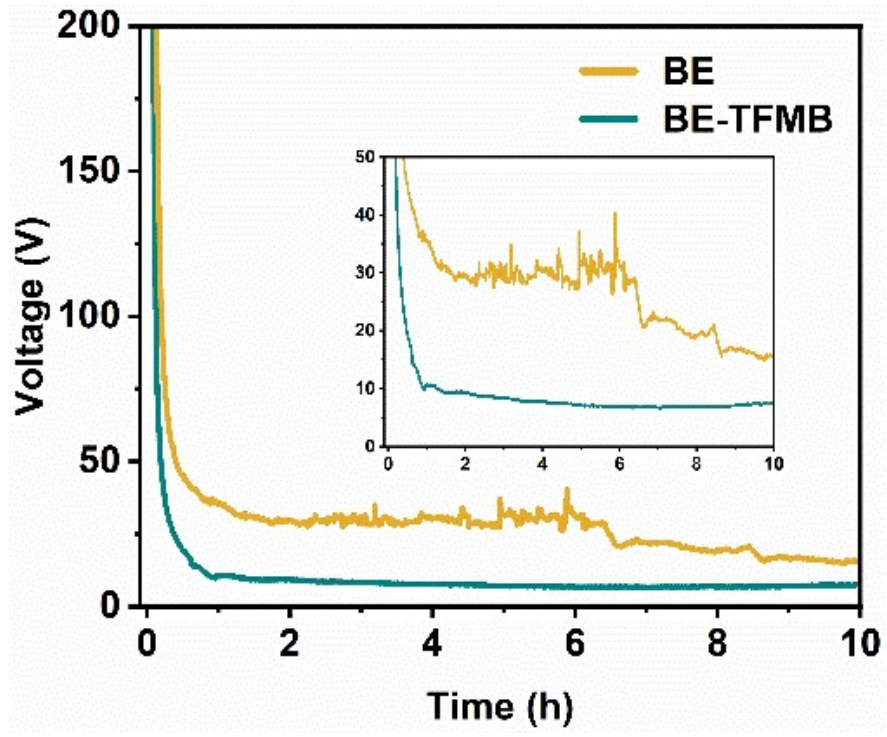

Figure S44. Leaking current curves of Li||NCM811 cells at 4.5 V and 50 °C.

First, electrochemical testing methods were used to investigate the stability of the electrolyte and interphases under high-temperature and high-voltage conditions. As shown in **Figure S44**, the high-voltage stability of the electrolytes was further assessed by aggressive floating tests. The Li||NCM811 cells were charged at constant voltage at 4.5 V for 10 h following an initial formation cycle. As expected, due to the high reactivity of carbonates in BE under high voltage, intense side reactions caused high leaking currents. In contrast, no significant leaking currents were observed in the BE-TFMB system, indicating suppressed electrolyte decomposition and stable interphases (*Angew. Chem. Int. Ed.* **2025**, 64, e20251599).

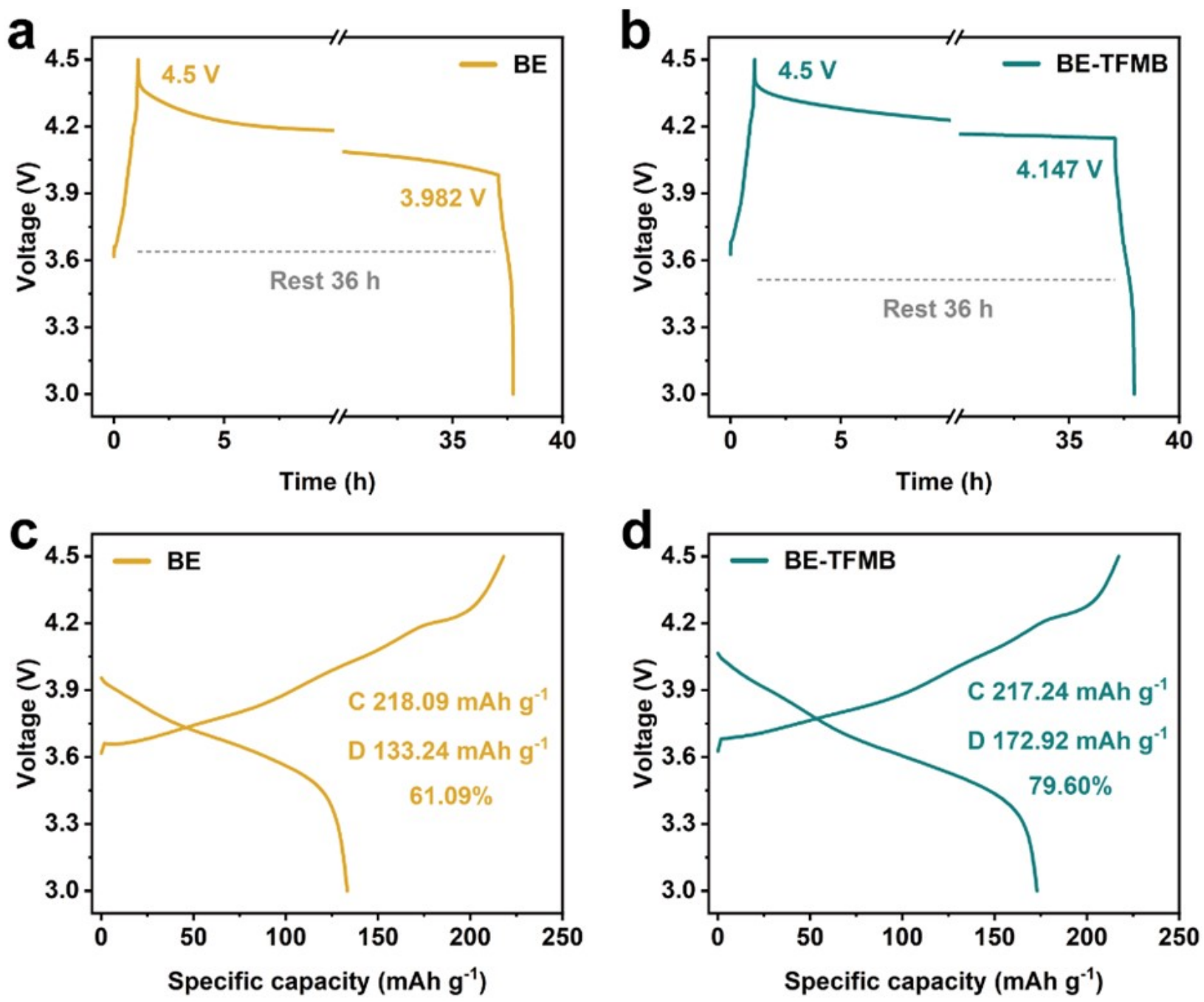

Figure S45. (a, b) Self-discharge curves and (c, d) corresponding charge/discharge curves of Li||NCM811 cells using (a, c) BE and (b, d) BE-TFMB at 4.5 V and 50 °C.

Self-discharge tests shown in **Figure S45** further validate the high-voltage durability of the BE-TFMB-derived interphases. After being charged to 4.5 V and storing for 36 h, Li||NCM811 cells using BE-TFMB maintained an open-circuit voltage of 4.147 V and released 79.60% of their initial charged capacity. However, BE-based cells delivered only 61.09% of their initial capacity, indicating more serious side reactions at high voltages (*J. Energy Chem.* **2026**, 116, 706–715; *Adv. Energy Mater.* **2026**, 16, e70894). These data are corroborated by the excellent overlap of successive dQ/dV plots, implying the effective mitigation of structural degradation in BE-TFMB-based cells. As depicted in **Figure 3i**, cells using BE-TFMB demonstrate distinct phase transition peaks with a smaller potential difference (*Cell Rep. Phys. Sci.* **2025**, 6, 102597).

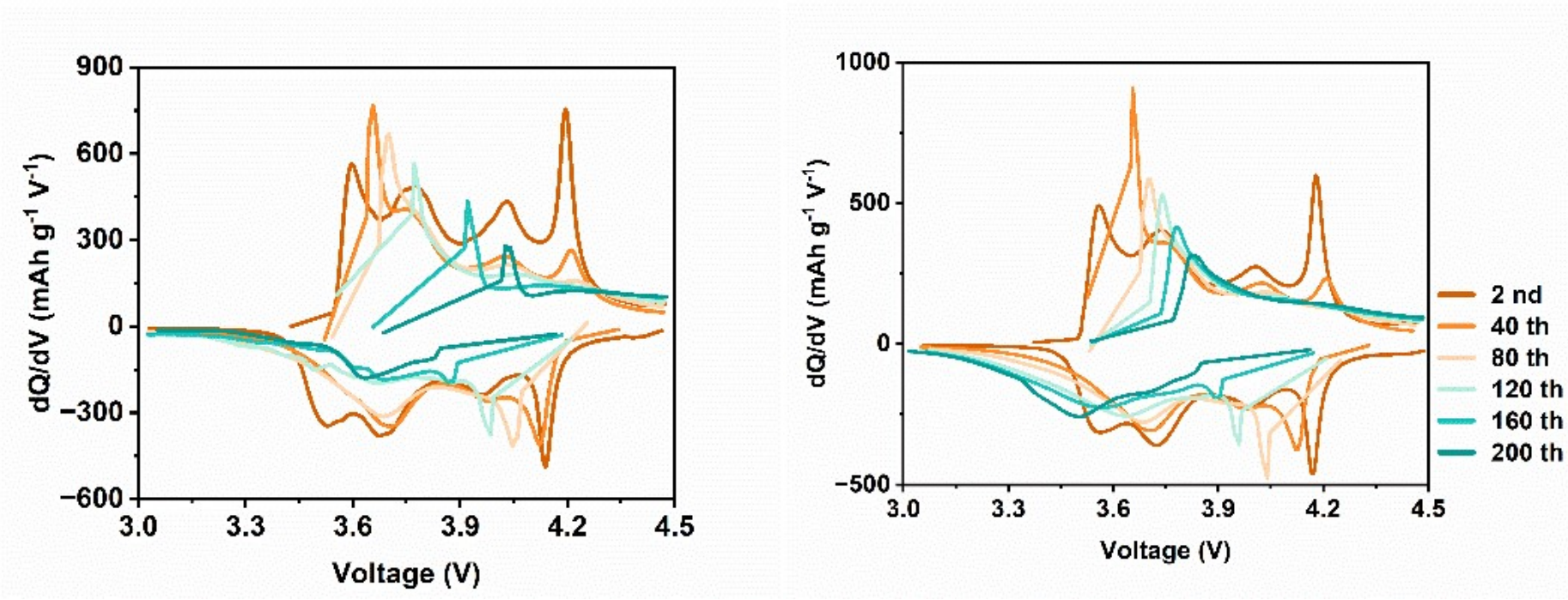

Figure S46. dQ/dV curves of Li||NCM811 cells containing (a) BE and (b) BE-TFMB at 4.5 V and 50 °C.

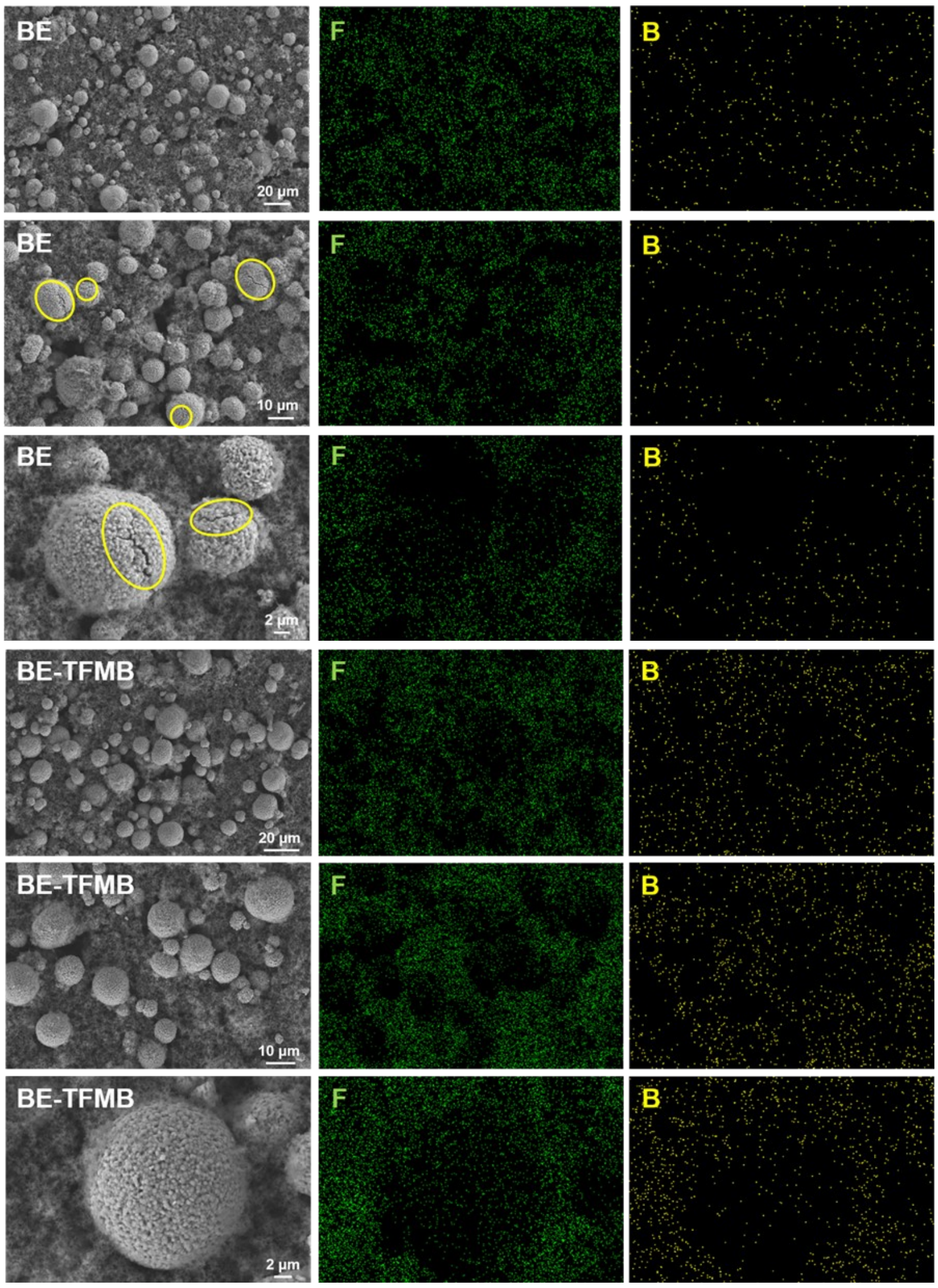

Figure S47. SEM images and corresponding EDS mapping of the cycled NCM811 cathode in BE and BE-TFMB.

Subsequently, SEM analysis was performed to investigate the effects of high-temperature and high-voltage conditions on the morphology and structure of the NCM811 cathode and Li anode. As shown in **Figure S47**, the cathode cycled in BE-TFMB was more compact and smoother (*ACS Nano* **2025**, 19, 33202−33211). In contrast, the cathode cycled in BE showed severely cracked particles, covered by uneven deposits. Furthermore, the corresponding elemental mapping images showed a uniform distribution of B and F signals on the cathode surface derived from BE-TFMB, further confirming that the interphases formed by TFMB are more conducive to cathode structural integrity, thereby ensuring the stable operation of Li||NCM811 cells under extreme conditions.

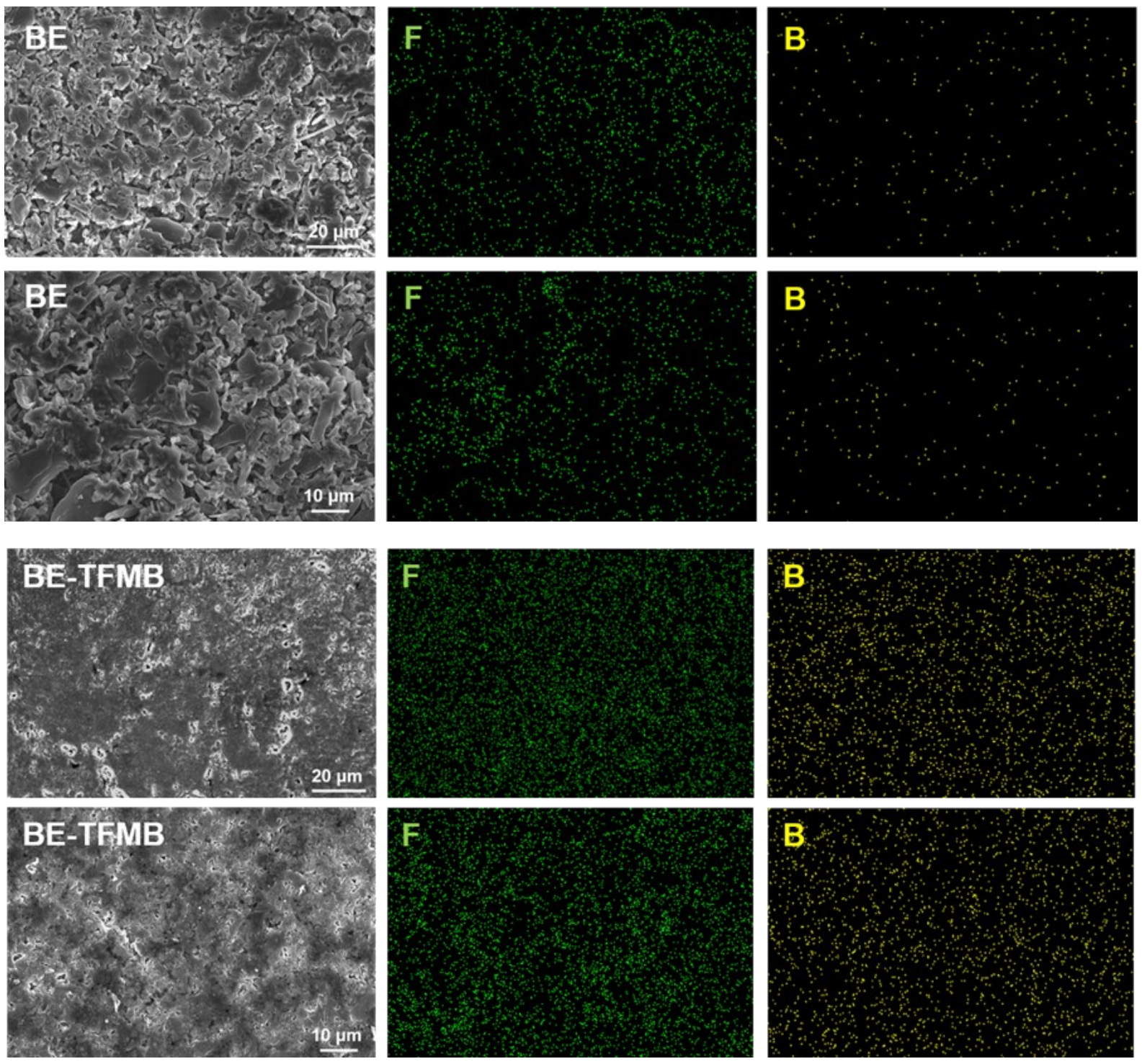

Figure S48. SEM images and corresponding EDS mapping of the cycled Li anode in BE and BE-TFMB.

Similarly, the surface of the Li anode in BE exhibited extensive dendrite growth after cycling, which may have resulted from the continuous fragmentation and reconstruction of unstable interphases, as well as intense electrolyte reactions (*Nano Lett*. **2025**, 25, 7762−7769). In contrast, the anode surface derived from BE-TFMB exhibited a smooth and dense morphology, free of dendrites (**Figure S48**).

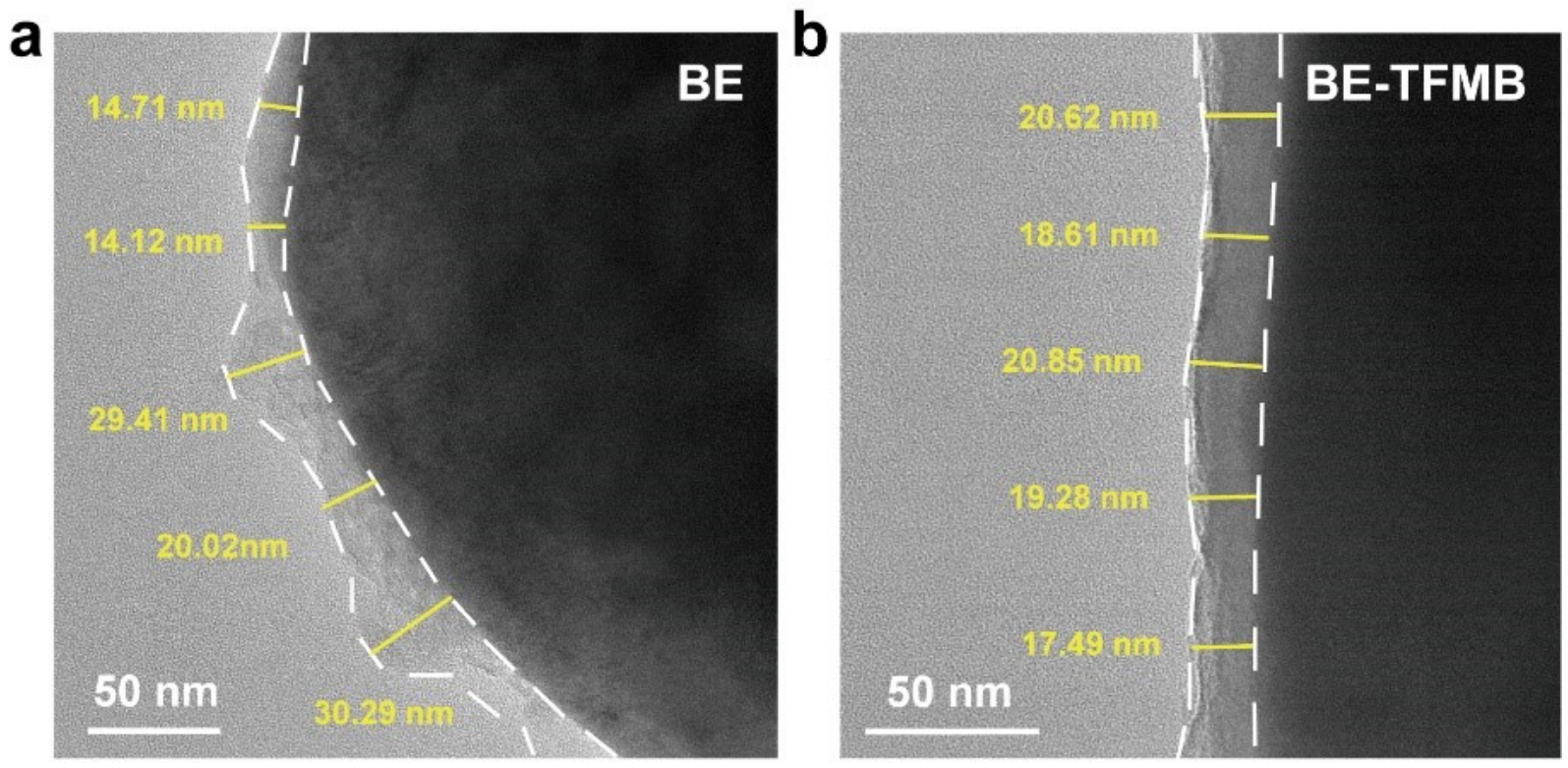

Figure S49. TEM images of the cycled NCM811 cathode in (a) BE and (b) BE-TFMB.

Furthermore, the corresponding TEM images show that the CEI in BE-TFMB is relatively dense and uniform in thickness (~20 nm). In contrast, the CEI in the BE system exhibits a non-uniform thickness, suggesting intense electrolyte side reactions and continuous fragmentation and reconstruction of the CEI (**Figure S49**).

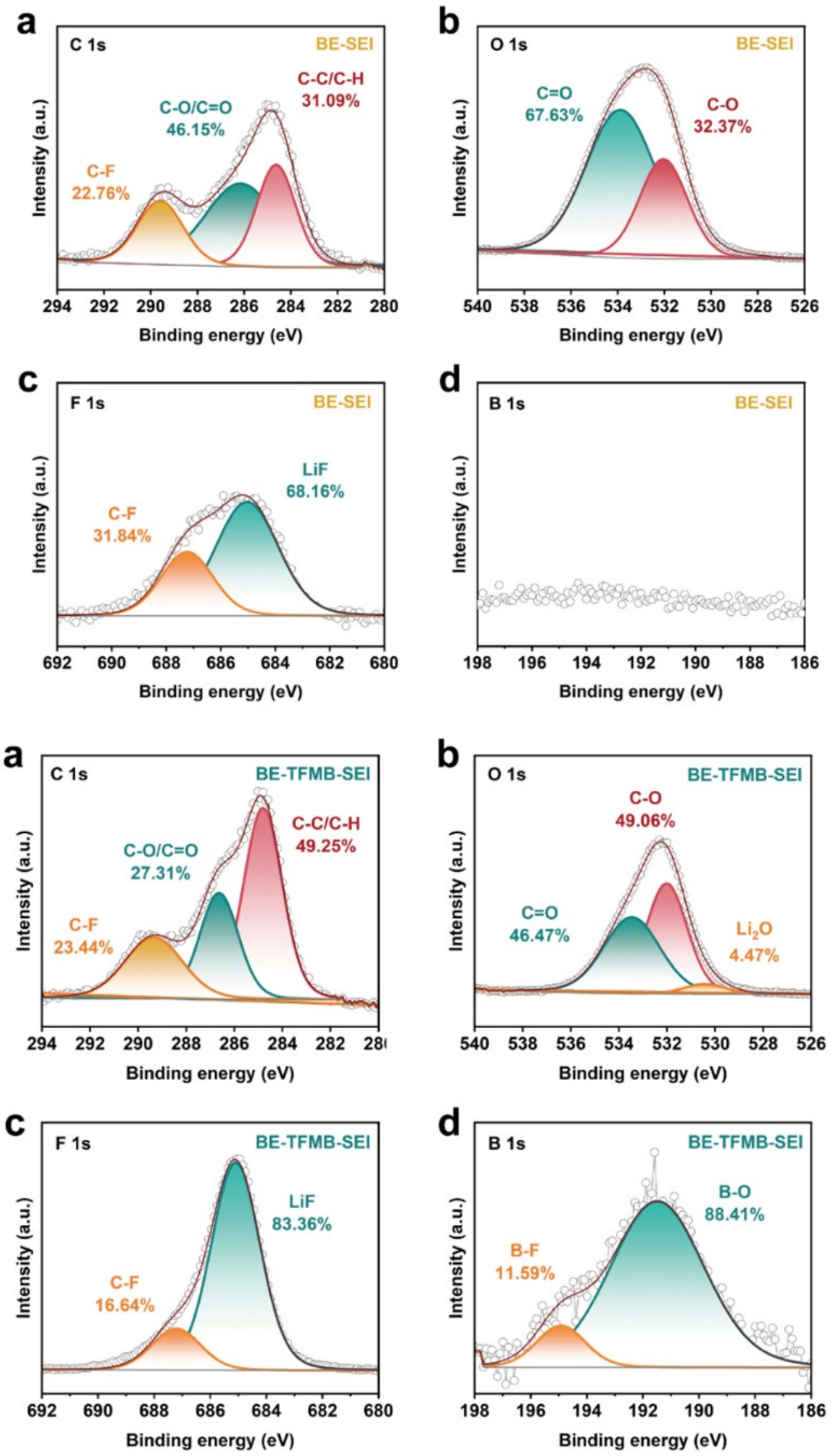

Figure S50. XPS spectra of (a) C 1s, (b) O 1s, (c) F 1s and (d) B 1s for cycled Li anode in BE and BE-TFMB.

The effects of extreme operating conditions on the composition of interphases were further investigated. The SEI formed by BE exhibited a high intensity of C=O/C-O signals, corresponding to violent side reactions of solvent molecules under harsh conditions. Importantly, the high LiF content in the F 1s spectra confirmed the formation of an inorganic-rich SEI in BE-TFMB, which inhibits further electrolyte penetration and uncontrolled side reactions (**Figure S50**). Moreover, the synergistic effect of B-F and other inorganic substances helps further enhance the mechanical stability of the interphases, while the abundant inorganic grain boundaries also facilitate Li^+ transport (*Nat. Commun.* **2023**, 14, 1082).

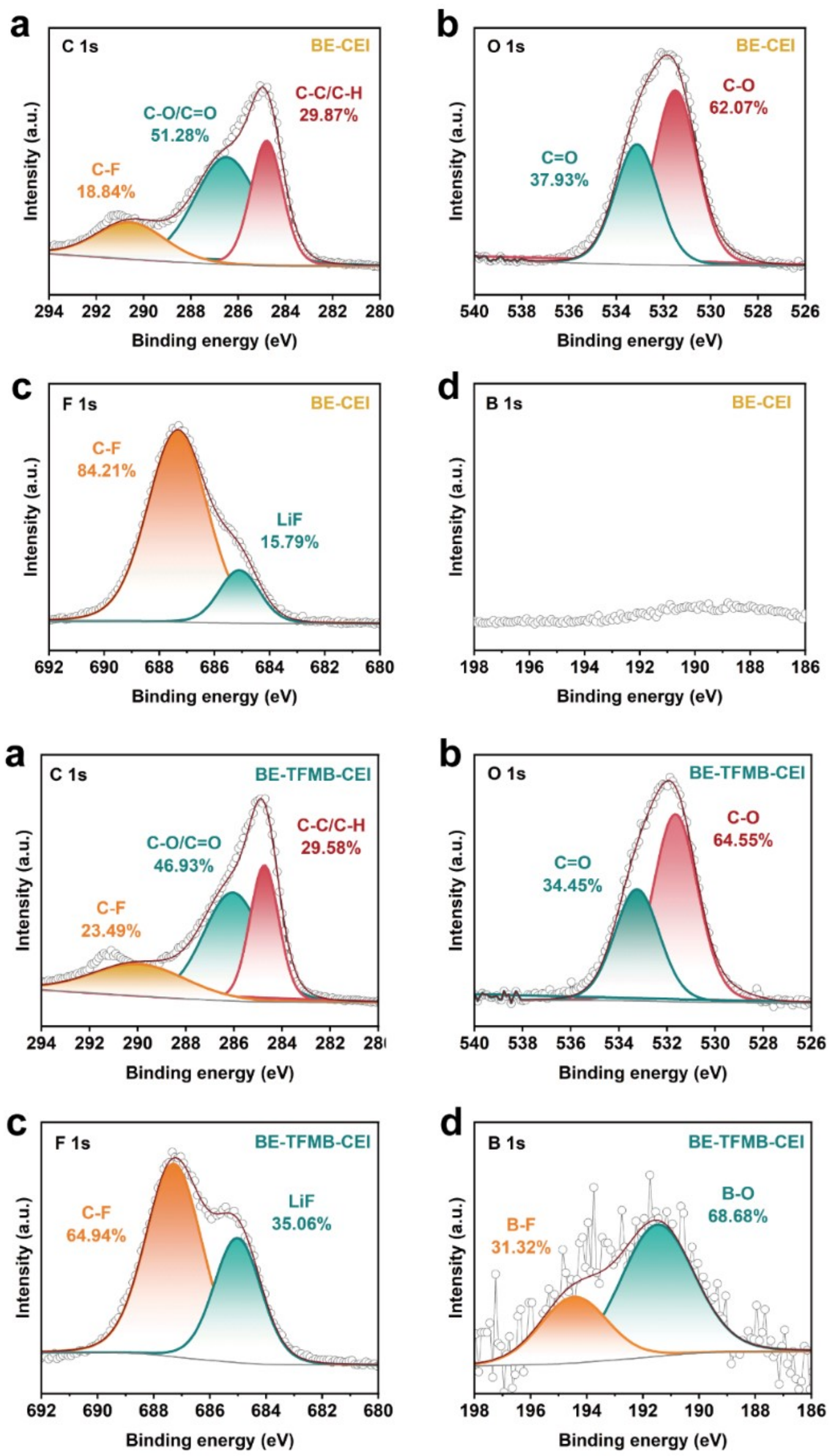

Figure S51. XPS spectra of (a) C 1s, (b) O 1s, (c) F 1s and (d) B 1s for cycled NCM811 cathode in BE and BE-TFMB.

As displayed in **Figure S51**, it is worth noting that the CEI derived from different electrolytes exhibits similar characteristics, further demonstrating that BE-TFMB can form uniform, compact, and inorganic-rich interphases.

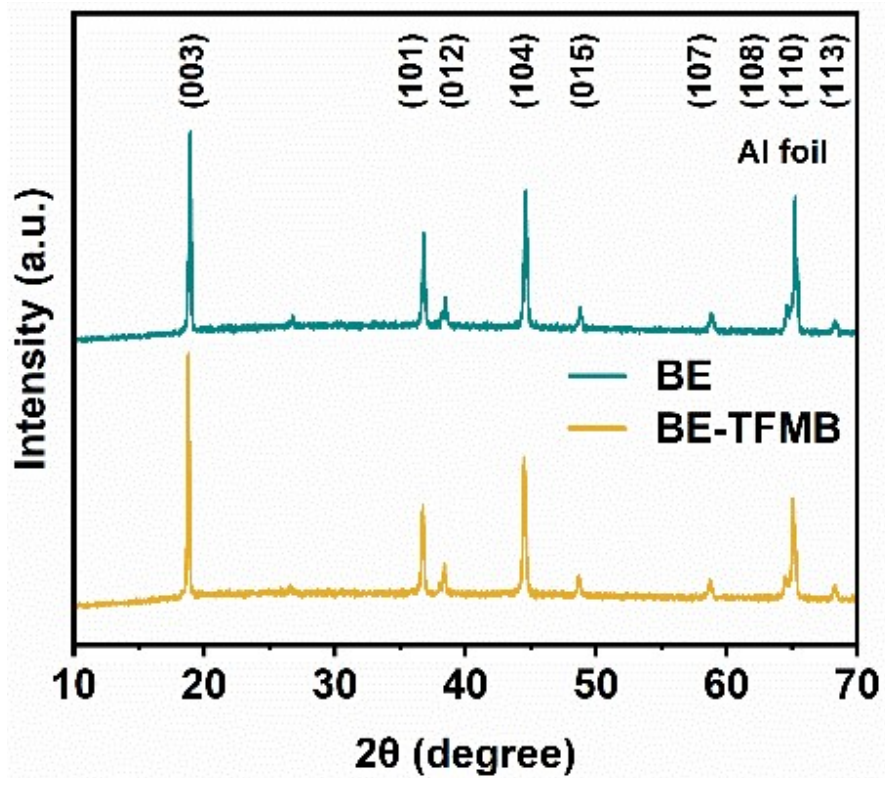

Figure S52. XRD patterns of cathodes after cycling at 4.5 V and 50 °C.

XRD testing was performed on the NCM811 cathode after cycling under severe conditions to determine the evolution of the cathode structure. As shown in **Figure S52**, all peak intensities in BE-TFMB are higher than those in BE. The higher I(003)/I(104) value of the NCM811 cathode in BE-TFMB verifies a lower Li/Ni mixing degree and is highly consistent with dQ/dV results (*ACS Nano* **2024**, 18, 25096−25106).

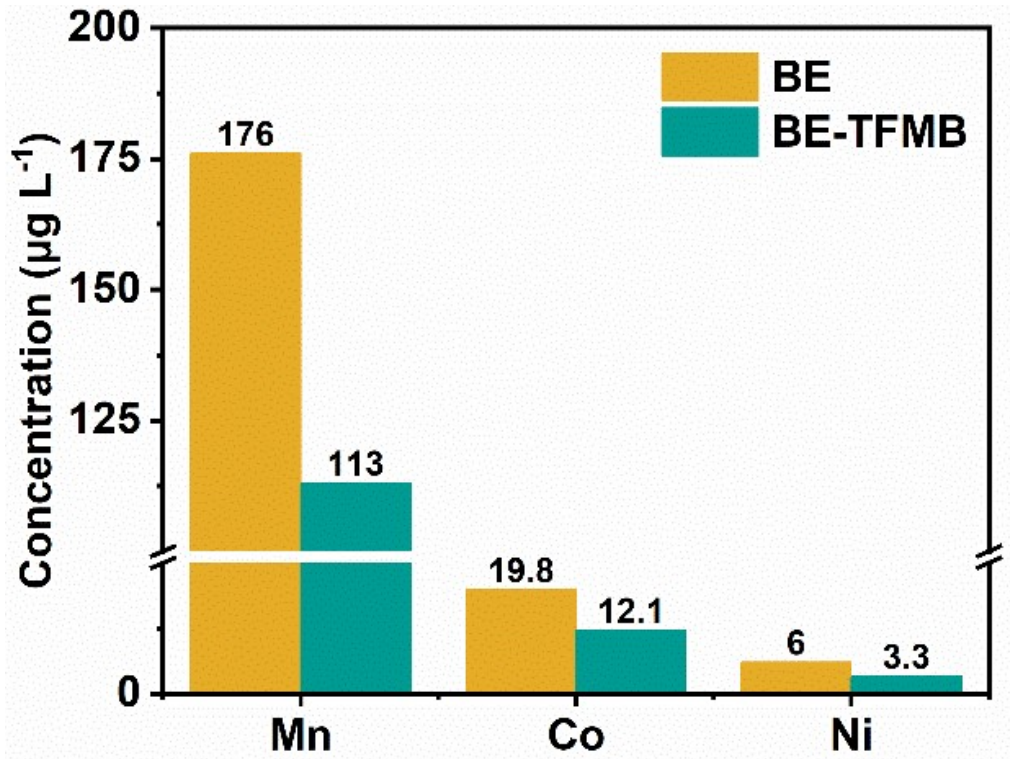

Figure S53. The concentrations of TMIs of Li anode after cycling at 4.5 V and 50 °C.

Consistent with this result, the ICP test results revealed that the surface of the Li anode in BE showed higher Ni, Co and Mn contents, which validates the superiority of a robust CEI in suppressing the dissolution of transition metal ions (**Figure S53**).

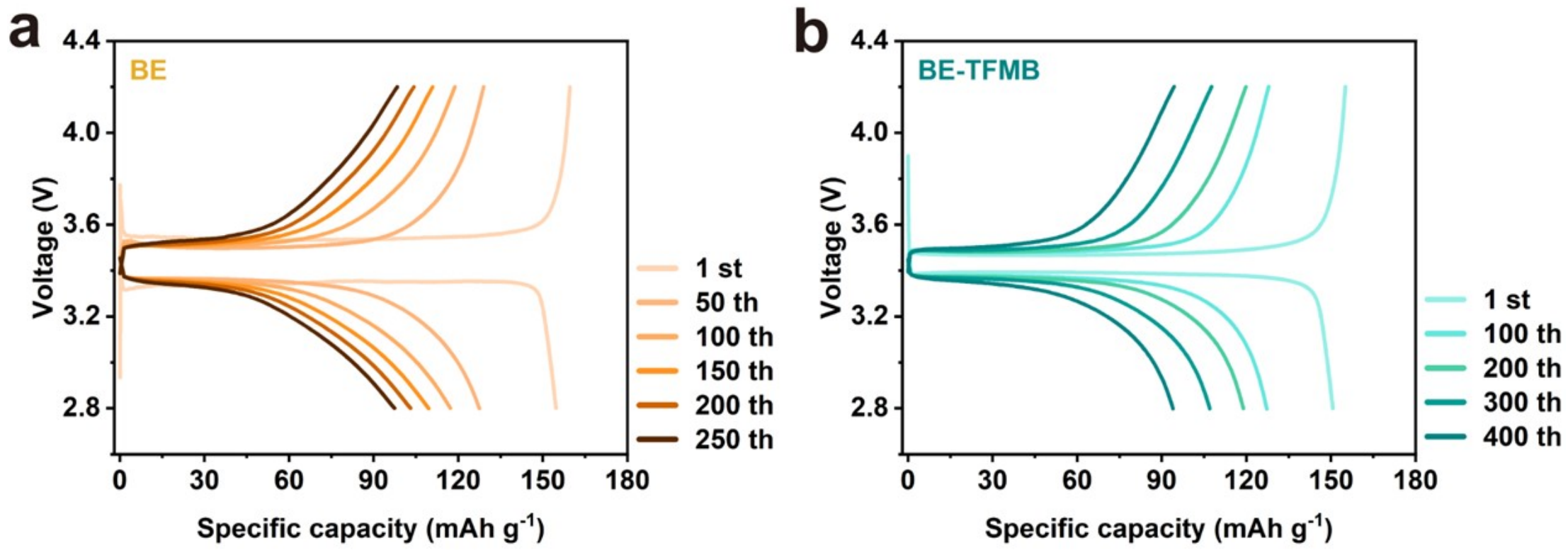

Figure S54. Charge/discharge curves of Li||LFP cells using (a) BE and (b) BE-TFMB.

Table S1. HF content in two electrolytes at different storage times (60 °C).

**Storage Time (days)**	**HF content (mol L^{-1})**	
	BE	BE-TFMB
4	40.39	0
8	282.71	60.58
12	424.06	100.97

Table S2. The conductivity values of BE and BE-TFMB at different temperatures.

Temperature (°C)		0	10	20	30	40	50
σ (mS cm^{-1})	BE	3.78	6.21	7.9	8.08	10.42	12.19
	BE-TFMB	6.56	8.57	8.88	9.45	13.25	14.31

Table S3. The R_i and R_{ct} values of Li||NCM811 cells with different electrolytes at different cycles.

Cycles number	Resistance (ohm)	Electrolyte	
		BE	BE-TFMB
1st	R_i	9.42	13.15
	R_{ct}	227.21	190.45
50th	R_i	15.69	16.60
	R_{ct}	83.76	69.23
150th	R_i	24.37	12.08
	R_{ct}	68.01	59.81

Table S4. Comparison of this work with reported electrolytes operating at high voltage condition.

Electrolyte	Maximum cut-off voltage	Highest operation temperature	Cycle number at 4.5 V	Rate at 4.5V	Capacity retention at 4.5 V	Ref.
**1.0 wt% 4-trifluoromethylbenzeneboronic acid neopentyl glycol ester 1.0 M $LiPF_6$ in ethylene carbonate:diethyl carbonate = 1:1 (v:v)**	**4.7 V**	**50 °C**	**300**	**1 C**	**75.25%**	**This work**
0.75 wt% 2,4,6-tris(3,4,5-trifluorophenyl)boroxin 0.7 M $LiBF_4$, 0.3 M LiDFOB Sulfone/1,2-diethoxyethane = 4:1 (v:v)	4.5 V	80 °C	350	0.5 C	80.70%	4
poly(ethylene glycol) diacrylate:2,2,2-trifluoroethyl methacrylate:tris(2-hydroxy ethyl) isocyanuratetriacrylate = 5:5:3 (w:w:w) 0.152 g $LiPF_6$, 0.029 g trimethyl borate, 0.023 g 1,2-bis(2-cya-noethoxy)ethane 0.5 mL EC:DEC = 1:1 (v:v)	4.5 V	30 °C	300	0.5 C	70.00%	5
glycol diglycidyl ether:tetrahydrofuran = 1:20 (w:w) 1 M LiFSI, 0.75 M LiDFOB	4.5 V	55 °C	150	1 C	85.00%	6
1.0 M LiTFSI, 0.5 M $LiPF_6$ Fluoroethylene carbonate:ethyl 2-fluoropropionate = 2:1 (v:v)	4.6 V	30 °C	300	1 C	82.51%	7
1.0 M $LiPF_6$, 0.1 M $LiBF_4$ methyl butyrate:fluoroethylene carbonate = 1:1 (v:v)"	4.5 V	30 °C	150	0.5 C	88.21%	8
1.0 M $LiPF_6$,1.0 wt% trimethoxyboroxine ethylene carbonate:diethyl carbonate = 3:7 (w:w)	4.7 V	25 °C	200	0.5 C	82.30%	9

References

[1] Designing C-Fe-O bonded MIL-88B (Fe)/jasmine petal-derived-carbon composite biosensor for the simultaneous detection of dopamine and uric acid. *Chemical Engineering Journal* **2021**, 404, 126570.

[2] Highly stable lithium metal batteries enabled by regulating the solvation of lithium ions in nonaqueous electrolytes. *Angewandte Chemie International Edition* **2018**, 57, 5301-5305.

[3] Ultrarobust, tough and highly stretchable self-healing materials based on cartilage-inspired noncovalent assembly nanostructure. *Nature communications* **2021**, 12, 1291.

[4] Electrolyte sustaining 4.5 V Li||NCM811 batteries cycled at 80 °C. *Advanced Energy Materials* **2026**, DOI:10.1002/aenm.70894 10.1002/aenm.70894.

[5] Molecular-level polymer design and interface engineering enable 4.5 V high-voltage Li metal batteries. *Energy Storage Materials* **2025,** 79, 104327.

[6] Trilogy design of cross-linked polyether electrolytes for high-voltage and wide-temperature lithium metal batteries. *eScience Energy* **2026,** 2, 100025.

[7] Enhancing lithium metal battery performance with a perfluorinated bisalt electrolyte achieving high-voltage stability up to 4.8 V. *Energy Storage Materials* **2025,** 75, 104048.

[8] Synergistic regulation of competitive interactions in solvation chemistry for high-voltage and low-temperature NCM811/Li batteries. *Energy Storage Materials* **2026,** 84, 104812.

[9] Construction of ionic conductive electrode/electrolyte interphases via Li^+ coordination regulator for 4.7 V Li/ $LiNi_{0.9}Co_{0.05}Mn_{0.05}O_2$ batteries. *Advanced Functional Materials* **2025,** 35, 2507638.
